# Ghosts of symbionts past: the hidden history of the dynamic association between filarial nematodes and their *Wolbachia* endosymbionts

**DOI:** 10.1093/g3journal/jkaf226

**Published:** 2025-10-01

**Authors:** Emmelien Vancaester, Guy R Oldrieve, Alex Reid, Georgios Koutsovoulos, Dominik R Laetsch, Benjamin L Makepeace, Vincent Tanya, Sven Poppert, Jürgen Krücken, Adrian Wolstenholme, Mark Blaxter

**Affiliations:** Tree of Life, Wellcome Sanger Institute, Hinxton CB10 1SA, United Kingdom; School of Biological Sciences, The University of Edinburgh, Edinburgh EH9 3TF, United Kingdom; School of Biological Sciences, The University of Edinburgh, Edinburgh EH9 3TF, United Kingdom; School of Biological Sciences, The University of Edinburgh, Edinburgh EH9 3TF, United Kingdom; School of Biological Sciences, The University of Edinburgh, Edinburgh EH9 3TF, United Kingdom; Institute of Infection, Veterinary & Ecological Sciences, The University of Liverpool, Liverpool L3 5RF, United Kingdom; Institut de Recherche Agricole Pour le Développement, Regional Centre of Wakwa, Ngaoundéré, Adamawa Region BP65, Cameroon; Bernhard Nocht Institute, Bernhard-Nocht-Straße 74, Hamburg 20359, Germany; Institute for Parasitology and Tropical Veterinary Medicine, Freie Universität Berlin, Berlin 14163, Germany; Veterinary Centre for Resistance Research, Freie Universität Berlin, Berlin 14163, Germany; Dept of Infectious Diseases, University of Georgia, 501 D W Brooks Drive, Athens, GA 30602, United States; Tree of Life, Wellcome Sanger Institute, Hinxton CB10 1SA, United Kingdom; School of Biological Sciences, The University of Edinburgh, Edinburgh EH9 3TF, United Kingdom

**Keywords:** horizontal gene transfer, *Wolbachia*, filarial nematode, phylogeny, symbiosis

## Abstract

Many, but not all, parasitic filarial nematodes (Onchocercidae) carry intracellular, maternally transmitted, alphaproteobacterial *Wolbachia* symbionts. The association between filarial nematodes and *Wolbachia* is often portrayed as mutualist, where the nematode is reliant on *Wolbachia* for an essential but unknown service. *Wolbachia* are targets for antifilarial chemotherapeutic interventions for human disease. *Wolbachia* of Onchocercidae derive from four of the major supergroups (C, D, F, and J) defined within the genus. We explored the evolutionary history of the filarial nematode-*Wolbachia* symbiosis in 22 nematode species, 16 of which have current *Wolbachia* infections, by screening the nematode nuclear genome sequences for nuclear *Wolbachia* transfers, fragments of the *Wolbachia* genome that have been inserted into the nuclear genome. We identified *Wolbachia* insertions in 5 of the 6 species that have no current *Wolbachia* infection, showing they have previously had and have now lost *Wolbachia* infections. In currently infected species, we found a diversity of origins of the insertions, including many cases where they derived from a different supergroup to the current live infection. Mapping the origins of the insertions onto the filarial nematode phylogeny we derive a complex model of evolution of *Wolbachia* symbiosis. The history of association between *Wolbachia* and onchocercid nematodes includes not only cospeciation, as would be expected from a mutualist symbiosis, but also loss (in the 5 *Wolbachia*-free species), frequent symbiont replacement, and dual infection. This dynamic pattern is challenging to models that assume host–symbiont mutualism.

## Graphical Abstract

**Graphical Abstract:**
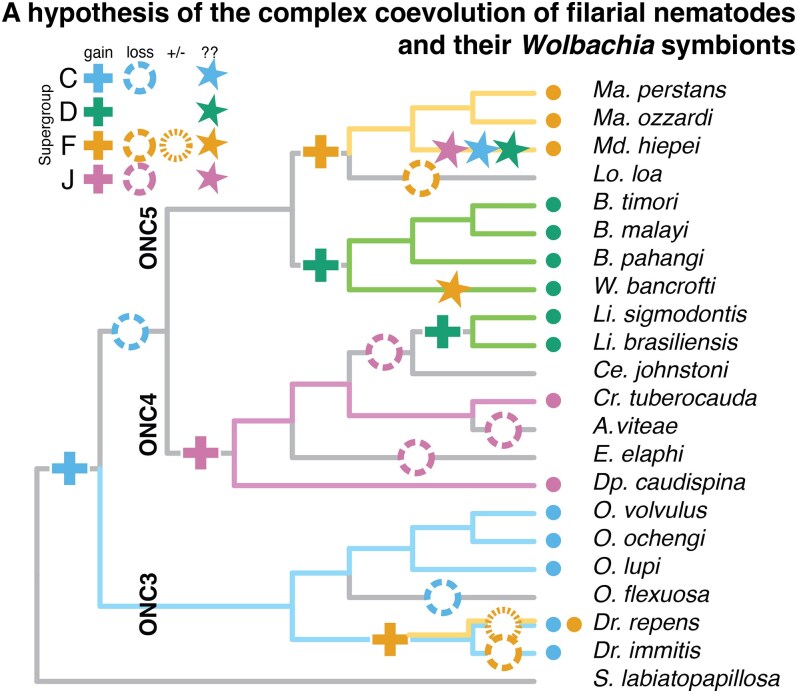

## Introduction

*Wolbachia* are alphaproteobacteria belonging to the Rickettsiales, a group characterized by an intracellular lifestyle and wide range of phenotypic effects on their eukaryotic hosts (Werren 1997; Lo et al. 2007). *Wolbachia* is a maternally transmitted endosymbiont of terrestrial arthropods and parasitic nematodes (Lefoulon et al. 2020a). In most arthropods *Wolbachia* endosymbionts are nonessential, and hosts can be cured of infection by treatment with antibiotics. Arthropod *Wolbachia* are characterized as reproductive parasites, as they generally promote their own transmission by reproductive manipulation, whereby female hosts carrying infections have higher Darwinian fitness than uninfected females (Werren 1997). In several insects, it has been shown that *Wolbachia* infection can also be beneficial by promoting host resistance to viral or protozoan parasite infection (Hedges et al. 2008; Teixeira et al. 2008). *Wolbachia* may thus play a role in disease epidemiology (Caragata et al. 2016; Schinkel et al. 2024). Some symbioses have a nutritional basis. For example, the *Wolbachia* of hematophagous bed bugs provide B vitamins to their hosts (Hickin et al. 2022). *Wolbachia* is classified into supergroups based on analysis of molecular markers (Lo et al. 2002, 2007; Gerth et al. 2014). Supergroups A and B are the most common and are restricted to terrestrial arthropods (largely in the Insecta) (Gerth et al. 2014). Most other supergroups also infect terrestrial arthropods but often have restricted host ranges. For example, supergroups I and V are found in fleas (Beliavskaia et al. 2023), supergroup P in quill mites (Glowska et al. 2015), and supergroup S in pseudoscorpions (Lefoulon et al. 2020b). *Wolbachia* have been described from plant- and animal-parasitic nematodes. Some *Pratylenchus* plant-parasitic nematodes are infected with supergroup L *Wolbachia* (Brown et al. 2018; Wasala et al. 2019). The tylenchoid nematode *Howardula* sp., which infects sphaerocerid flies, was shown to contain *Wolbachia* with an unusually small genome, assigned to supergroup W (Dudzic et al. 2022). Most *Wolbachia* infections have been described in the family Onchocercidae or filarial nematodes. Filarial nematodes are parasites of vertebrates, including humans, and many have been shown to carry *Wolbachia* (Bouchery et al. 2013; Lefoulon et al. 2016). The filarial nematode *Wolbachia* are from supergroups C, D, F, and J (Lefoulon et al. 2016; Sinha et al. 2023b). Interestingly, *Wolbachia* supergroup F also occurs in arthropods (Lo et al. 2002).

*Wolbachia* infection of onchocercine nematodes is usually portrayed as more mutualist than parasitic (Fenn and Blaxter 2004). The distinction is important for treatment strategies for human parasites. Experimental treatment of *Wolbachia*-infected species with tetracycline antibiotics eliminates the bacteria but also harms the nematode, leading to infertility and death, while uninfected nematode species are unaffected (Hoerauf et al. 1999). Tetracycline has been shown to be effective in suppressing patent infection and in promoting adult nematode clearance (Taylor et al. 2005; Ehrens et al. 2022). Close examination of the transmission biology of *Wolbachia (Landmann*  et al*. 2012)* and the impact of deoxycycline treatment identifies death of *Wolbachia* and apoptosis of germline cells in female nematodes as the likely causative process in the efficacy of antibiotic treatment (Landmann et al. 2011).

While the responses to antibiotic treatment suggest a dependent relationship between nematode and *Wolbachia*, these observations do not prove mutualism (Fenn and Blaxter 2004). Strict mutualism should be reflected in close cospeciation between host and bacterium and positive evidence of metabolic dependency, as is observed in the aphid-*Buchnera* system (Bennett and Moran 2015). In insect *Wolbachia* there is little evidence for close host-*Wolbachia* coevolution, though there is preferential association of supergroup A with dipteran and hymenopteran hosts and supergroup B with lepidopteran and hemipteran hosts (Vancaester and Blaxter 2023). Early analyses suggested extensive cophylogeny between *Wolbachia* in supergroups C and D with their nematode hosts (Bandi et al. 1998), but subsequent wider analyses identified uninfected species nested within infected clades and breaks in cophylogeny, suggesting loss and replacement may be common (Lefoulon et al. 2016). It has not been possible to identify evidence of biochemical essentiality of filarial *Wolbachia*, though supplementation of host Fe scavenging pathways has been identified as a possible *Wolbachia* contribution (Wu et al. 2013). If *Wolbachia* are not beneficial mutualists, why is the infection so common and apparently universal in infected species (Fenn and Blaxter 2004)? In arthropods, *Wolbachia* are reproductive manipulators, through mechanisms that include killing of embryos of infected mothers when mated with infected fathers and killing of male embryos, mediated by diverse toxin–antitoxin systems that interact with host chromatin (Landmann et al. 2009; LePage et al. 2017). If the filarial *Wolbachia* were similarly reproductive manipulators, the observed association could be a form of ransom or addictive parasitism (Sullivan 2017).

Lateral transfers of *Wolbachia* DNA into the host nuclear genome (nuclear *Wolbachia* transfers, or NUWTs) have been identified frequently in filarial nematodes (Fenn et al. 2006; Dunning-Hotopp et al. 2007; McNulty et al. 2012; Ioannidis et al. 2013; Sinha et al. 2023a) and in arthropods (Kondo et al. 2002; Dunning-Hotopp et al. 2007; Klasson et al. 2009, 2014; Doudoumis et al. 2012; Funkhouser-Jones et al. 2015). Insertions have been observed in the genomes of individuals not currently infected in both nematodes (Koutsovoulos et al. 2014) and arthropods (Queffelec et al. 2022). These “fossil” NUWTs provide evidence of past infection in the lineage leading to the sequenced individual. Alternate models where the NUWTs are not records of past infection in the lineage of their host but rather have been horizontally transferred from other lineages (either by introgression from sister species or from horizontal transfer from unrelated species) are unlikely. Filarial nematodes are male–female species, and there is no literature suggesting any particular propensity to hybridize, and no evidence for ancient hybridization has been presented based on molecular phylogenetic incongruences. Acquisition of NUWTs as part of nonintrogression horizontal gene transfer is also vanishingly unlikely, requiring an extremely unlikely event to particularly include a minor component of a putative source genome. NUWT insertions range in length from a few hundred base pairs to near-full *Wolbachia* genomes (Dunning-Hotopp et al. 2007; Klasson et al. 2014; Tvedte et al. 2022). There is no strong evidence that insertions are functional, and most display evidence of disabling mutations and pseudogenization (Blaxter 2007).

To explore the phylogenetic history of the dynamics of host–symbiont interaction, we developed a toolkit to robustly identify NUWTs in host genomes. Here, we use this “fossil record” of NUWTs to explore the dynamics of *Wolbachia* association with these nematode hosts. We identify a surprisingly mobile symbiome, with multiple instances of endosymbiont loss and endosymbiont replacement.

## Materials and methods

### Sequencing and assembly of *Setaria labiatopapillosa* and *Acanthocheilonema viteae* genomes

A single cryopreserved specimen of *S. labiatopapillosa* was supplied by co-author Vincent Tanya from the zebu (*Bos indicus*) tissue obtained from Ngaoundéré abattoir, Adamawa Region, Cameroon. After pulverization of the specimen, DNA was extracted using a standard proteinase K digestion, followed by phenol-chloroform extraction and precipitation using isopropanol. DNA was pelleted by centrifugation and suspended in extraction buffer (EB; 10 mM Tris.HCl ph 8). An *A. viteae* DNA sample was provided by Kenneth Pfarr from the University of Bonn in Germany, from a laboratory strain maintained in mongolian jirds but originally isolated from a Lybian jird (*Meriones libycus*) in 1951 (Baltazard et al. 1952). RNA was extracted from nematodes using a Trizol extraction (see dx.doi.org/10.17504/protocols.io.kqdg3wje7v25/v1).

One genomic library of approximately 300 bp insert size was prepared for each species by Edinburgh Genomics and sequenced (paired end, 100 bases) on Illumina HiSeq 2500. Transcriptomic data from total mRNA from whole adults for each species were generated on Illumina using poly(A)-selected TruSeq RNA libraries. No other steps for elimination of ribosomal RNA were performed. Sequence fastq files were submitted to and are available freely from the Short Read Archive under bioprojects PRJEB7555 (*S. labiatopapillosa*) and PRJEB1697 (*A. viteae*). The quality of Illumina reads was checked with FASTQC (http://www.bioinformatics.babraham.ac.uk/projects/fastqc). Error correction was carried out using BLESS (Heo et al. 2014). A preliminary assembly was screened for potential contaminating mammalian host sequences with BlobToolKit (Challis et al. 2020). Production assemblies were carried out using SPAdes (Bankevich et al. 2012) (version 3.12.0). The assemblies were scaffolded with SCUBAT2 (https://github.com/GDKO/SCUBAT2) using cognate RNAseq data. Contigs less than 500 bp were discarded. The assembly is available in INSDC as accession GCA_966190365.

### Identification and assembly of 2 strains of *Wolbachia* from *Dirofilaria repens*

We sequenced a DNA extract from a *Dr. repens* adult nematode from an experimentally infected cat (infected with a canine isolate from Italy described in Yilmaz et al. (2016) (Yilmaz et al. 2016); the ethics statement concerning the sampling of this nematode is available therein). A genomic library of approximately 300 bp insert size was prepared by Edinburgh Genomics and sequenced (paired end, 100 bases) on Illumina HiSeq 2500. Low-quality bases and adapter sequences were removed using fastp (Chen et al. 2018) (version 0.12.3) (cut_by_quality, cut_window_size 4, cut_mean_quality 20). A preliminary assembly was generated using SPAdes (version 3.12.0). BlobToolKit analysis (version 1.0) revealed the presence of 3.04 Mb of scaffolds that could be assigned to *Wolbachia* based on sequence identity. These scaffolds were grouped into 2 clusters with distinct GC content and read coverage (Supplementary Fig. 1). Reads were mapped to both bins and the 2 genomes assembled using megahit (version 1.2.9) (Li et al. 2015). The resulting assemblies are summarized in Supplementary Supplemental Table 1. The assemblies have been submitted under BioProject PRJEB95945 as GCA_966211175.

### Data collation

We collated the genome data from 22 species of filarial nematode (Table 1; Supplementary Supplemental Table 1). Note that we used the published genome assembly of *Dr. repens* (GCA_008729115.1; Cafarelli et al. 2019) as it had much greater contiguity and higher completeness than either of the 2 short-read assemblies we produced in assembling the *Wolbachia* genomes for this species. *Wolbachia* genome sequences were downloaded from NCBI Genomes and complemented with the assembled *Dr. repens Wolbachia* genomes (Supplementary Table 2). *Wolbachia* is robustly placed within the Rickettsiales in Alphaproteobacteria, and previous work has identified species in *Anaplasma*, *Ehrlichia,* and the newly described Candidatus *Mesenet* as its closest relatives. We added the genomes of 2 *Mesenet* isolates (*Mesenet* endosymbiont of *Phosphuga atrata*, GCA_964020175.1, and *Mesenet* endosymbiont of *Agriotes lineatus*, GCA_964019585.1), 2 *Ehrlichia* species (*Ehrlichia chaffeensis*, CP000236, and *Ehrlichia ruminantium*, CR925677) and 2 *Anaplasma* species (*Anaplasma centrale*, CP001759, and *Anaplasma marginale*, CP001079) as the outgroup. The dataset of 1,444 *Wolbachia* genomes is largely composed of genomes from supergroups A and B, with good representation of filarial-nematode-infecting strains. Supergroup C *Wolbachia* were from the following hosts: *Dr. immitis* (Lefoulon et al. 2020c), *O. gibsoni* (Roberts 2023), *O. gutturosa* (Scholz et al. 2020), *O. ochengi* (Darby et al. 2014), and *O. volvulus* (GCA_000530755.1). Supergroup D *Wolbachia* were included for the following hosts, *B. malayi* (Lefoulon et al. 2019), *B. pahangi* (Lebov et al. 2020), *Li. brasiliensis* (Lefoulon et al. 2020c), *Li. sigmodontis* (Stevens et al. 2024), and *W. bancrofti* (Chung et al. 2017). Supergroup F *Wolbachia* were included from nematode hosts *Ma. perstans* (Chung et al. 2017), *Ma. ozzardi* (Sinha et al. 2023b), and *Md. hiepei* (Lefoulon et al. 2020c), and from the arthropod hosts *Cimex lectularius* (Nikoh et al. 2014; Lefoulon et al. 2020c), *Ctenocephalides felis* (Beliavskaia et al. 2023), *Melophagus ovinus* (Sinha et al. 2023b), *Menacanthus eurysternus* (Mahmood et al. 2023), *Osmia caerulescens* (Sinha et al. 2023b), and *Penenirmus auritus* (Mahmood et al. 2023). Supergroup J *Wolbachia* from *Cr. tuberocauda* (Lefoulon et al. 2019), *Dp. caudispina* (Lefoulon et al. 2019), and *Dp. gracile* (Costa et al. 2023) were included.

**Table 1.** Nematode genome data.

Species	Sequencing center	Assembly version	BUSCO Nematoda_odb11		span	contig N50	Reference
			Complete	Duplicated			
*A. viteae*	University of Edinburgh	GCA_900537255.1	88.8	1.7	77350906	25808	This study
*Brugia malayi*	WormBase Parasite/EBI	GCF_000002995.4	98.2	0.5	87155713	14214749	Tracey et al. (2020)
*Brugia pahangi*	University of Maryland	GCA_012070555.1	97.5	2.4	96392917	10892846	Mattick et al. (2020)
*Brugia timori*	Wellcome Sanger Institute	GCA_900618025.1	56.8	0.5	64930714	2306	International Helminth Genomes Consortium (2019)
*Cercopithifilaria (Ce.) johnstoni*	Wellcome Sanger Institute	GCA_916381525.1	95.5	0.2	76938708	99003	McCann et al. (2021)
*Cruorifilaria (Cr.) tuberocauda*	New England Biolabs	GCA_013365365.1	96.1	0.4	75516636	105487	Lefoulon et al. (2020c)
*Dipetalonema (Dp.) caudispina*	New England Biolabs	GCA_013365325.1	97.8	0.2	81585392	132317	Lefoulon et al. (2020c)
*Dirofilaria (Dr.) immitis*	CIBIO-InBIO	GCA_024305405.1	94.1	0.4	86813779	4253153	Gomes-de-Sá et al. (2022)
*Dirofilaria (Dr.) repens*	ETH/UZH	GCA_008729115.1	94.3	3.9	99578628	584065	Cafarelli et al. (2019)
*Elaeophora elaphii*	Wellcome Sanger Institute	GCA_000499685.1	87.2	0.3	82568297	99433	International Helminth Genomes Consortium (2019)
*Litomosoides (Li.) brasiliensis*	New England Biolabs	GCA_013365375.1	95.1	0.2	65202511	147890	Lefoulon et al. (2020c)
*Litomosoides (Li.) sigmodontis*	Wellcome Sanger Institute	GCA_963070105.1	95.5	0.3	65877608	10903439	Stevens et al. (2024)
*Loa (Lo.) loa*	Broad Institute	GCF_000183805.2	97.4	0.1	91365832	174388	Desjardins et al. (2013)
*Madathamugadia (Md.) hiepei*	New England Biolabs	GCA_013365335.1	88.3	10.1	77701753	17407	Lefoulon et al. (2020c)
*Mansonella (Ma.) ozzardi*	New England Biolabs	GCA_029876185.1	93.8	0.8	76054666	285814	Sinha et al. (2023a)
*Mansonella (Ma.) perstans*	Institute of Tropical Medicine, Tuebingen, Germany	GCA_947561605.2	94.7	0.4	79599925	173569	Rodi et al. (2023)
*Onchocerca flexuosa*	McDonnell Genome Institute	GCA_002249935.1	71.8	0.6	67740367	540294	McNulty et al. (2013)
*Onchocerca lupi*	Northern Arizona University	GCA_028564675.1	95.6	0.6	92491485	96493	Roe et al. (2022)
*Onchocerca ochengi*	Wellcome Sanger Institute	GCA_000950515.2	85.1	0.6	91660559	16199	International Helminth Genomes Consortium (2019)
*Onchocerca volvulus*	Wellcome Sanger Institute	GCA_000499405.2	97.9	0.6	96340582	25485961	Cotton et al. (2017)
*S. labiatopapillosa*	University of Edinburgh	GCA_966190365	97.1	0.3	82384673	129940	This study
*Wuchereria bancrofti*	Case Western Reserve University	GCA_005281725.1	97.9	1	88416250	12368652	Small et al. (2019)

The quality of the *Wolbachia* genomes was assessed using CheckM (Parks et al. 2015) (version 1.2.2). We deployed dRep dereplicate (Olm et al. 2017) (version 3.4.3) to reduce the redundancy in the dataset, yielding a representative dataset of 165 genomes (see Supplementary Table 3). A database of these 165 *Wolbachia* genomes was built with kraken (Wood et al. 2019) (version 2.0.7), and all nematode nuclear genomes were screened for similarity to *Wolbachia* in BlobTools using BLASTn search versus the NCBI nt nucleotide database. All flagged contigs and scaffolds were manually assessed with NCBI BLAST + (Altschul et al. 1990) to detect all those having high similarity (>99%) to *Wolbachia* over the full length of the contig. We decided to remove these sequences from the nuclear assemblies as they most likely derived from living *Wolbachia* rather than nuclear insertions. A list of discarded sequences can be found in Supplementary Table 3.

### Nematode phylogenetic analysis

BUSCO (Manni et al. 2021) (version 5.4.7) was run on the genomes of 22 filarial nematodes to detect conserved single-copy genes using the nematode gene set (Nematoda_odb10). Retaining only those genes present across all genomes yielded a set of 670 protein-coding genes. These protein sequences were aligned using MAFFT (Katoh and Standley 2013) (version 7.490) in automatic mode. These alignments (a total of 409,399 positions) were analyzed with IQ-TREE (Minh et al. 2020) (version 2.1.4) using protein model GTR20 + G4 with 1,000 bootstraps. The phylogeny was rooted with *S. labiatopapillosa*.

### *Wolbachia* phylogenetic analysis

We repredicted proteomes for the 165 selected *Wolbachia* genomes with prokka (Seemann 2014) (version 1.14.6), which uses prodigal (Hyatt et al. 2010) (version 2.6.3) for gene finding. While this automated procedure may introduce errors, especially in prediction of proteins from pseudogenes, prodigal is a robust and accurate gene finder and the kinds of errors it may introduce were unlikely to affect later stages of our analyses. In particular, strain-specific mispredictions will be classified as strain-unique singletons and excluded from subsequent analyses as uninformative. Orthologs were predicted across these proteomes using OrthoFinder (Emms and Kelly 2015) (version 2.4.0). Orthologous families were reduced to only retain dRep-selected genomes. We selected the 696 clusters where at least 75% of the 165 *Wolbachia* strains were single-copy. The protein alignments for these genes were aligned with MAFFT (version 7.490) and trimmed of ambiguous regions using trimAl (Capella-Gutierrez et al. 2009) (version 1.4; -gt 0.25 -st 0.001). The trimmed alignments were concatenated into an alignment of 216,966 amino acid positions and analyzed with IQ-TREE (version 2.2.5) using protein model GTR20 + G4 with 1000 bootstraps. Additionally, a maximum likelihood phylogenetic tree for each of the 696 genes was inferred with IQ-TREE (version 2.1.4; iqtree -s {alignment} -nt {threads}). Coalescent analysis of these gene trees was performed using ASTRAL (Mirarab et al. 2014) (version 5.7.4). The gene-wise coalescent tree was compared to the concatenated sequence tree and no differences in tree topology were observed.

### Construction of hidden Markov models and screening of nuclear genomes for NUWTs

Protein sequences of the 2,647 orthologous groups that had 3 or more members across more than 3 *Wolbachia* strains were aligned using MAFFT (version 7.490). The untrimmed MAFFT protein alignment for each cluster was transformed to a nucleotide alignment of the source *Wolbachia* genomic sequences using tranalign from EMBOSS (Rice et al. 2000) (version 6.6). Each alignment was used to derive a nucleotide hidden Markov model (HMM) in hmmbuild from the hmmer suite (Eddy 2011) (version 3.3.1). The set of 2,647 HMMs was screened against each of the cleaned target nematode genomes using nhmmscan. The tabular output format of nhmmscan was parsed using a python script (SelectMatches.py) to identify putative NUWTs with E-values less than 1e-30. Matches were found for 44.5% (1,179) of the HMMs. We recognize that the nematode genome assemblies have been generated from different data types, are of different qualities (from highly fragmented to chromosomally scaffolded), and have undergone different contamination purging processes and thus that the NUWT repertoire of the lower assembly quality genomes may be incomplete. As noted above, for the highly fragmented assemblies, we removed from the NUWT catalogue any small contigs that had similarity >99% over the full length to the cognate *Wolbachia* genomes.

The coordinates of the matches were used to direct extraction of the corresponding span of nucleotide sequence from the nuclear genomes (FetchRegionSeq.py). These nucleotide sequences were profile aligned to the cognate tranalign alignments using MAFFT. Where the resulting alignment contained more than fifty supergroup A or supergroup B *Wolbachia* sequences, only dRep selected genomes were retained for these supergroups (using python script Reduce_alignment.py). The alignment was used to position the putative NUWTs within the diversity of the sequences from living *Wolbachia* using IQ-TREE (version 2.1.4) with a substitution model search (-m MFP) (Kalyaanamoorthy et al. 2017).

To identify the likely supergroup-of-origin of the NUWTs, the 1,179 output trees were traversed with a python script (Reroot_rename_tree.py) that uses the ete package from etetoolkit (Huerta-Cepas et al. 2016) (version 3.1.2). Trees were first rooted, either using outgroup sequences or, if these were not present, by midpoint rooting. All *Wolbachia* sequences were renamed according to their supergroup classification and nodes that had a uniform classification were collapsed. The placement of all putative NUWTs in their trees was assessed, and NUWTs were classified according to the supergroup of their closest sister branch.

NUWT locations were converted to bed files, and NUWTs were clustered into regions using bedtools (Quinlan and Hall 2010) (version 2.29.0) if the next NUWT was located within 2.5 kb and there were no intervening coding genes.

### Nematode gene and repeat annotation

For all 22 nematode genomes, we performed de novo repeat identification using EarlGrey (Baril et al. 2024) (version 3.0). We provided the curated repeat libraries to RepeatMasker (https://www.repeatmasker.org/; version 4.1.5) to soft-mask transposable elements. Protein-coding annotation for the filarial nematodes was taken from the following sources: Wormbase Parasite (Howe et al. 2017) for *A. viteae*, *B. pahangi*, *B. timori*, *Ce. johnstoni*, *Dr. immitis*, *E. elaphii*, *Lo. loa, O. flexuosa*, *O. ochengi*, and *W. bancrofti*; a public Braker annotation (Sinha et al. 2023a) for *Cr. tuberocauda*, *Dp. caudispina*, *Li. brasiliensis*, *Md. Hiepei*, and *Ma. Ozzardi*; and an Augustus annotation (Sinha et al. 2023a) for *M. perstans*. Liftoff (Shumate and Salzberg 2021) (version 1.6.3) was used for *A. viteae*, *B. pahangi*, *Ma. perstans*, and *W. bancrofti* to transfer the annotation to the genome version used in this study. For the four species without public annotation; *Dr. repens*, *Li. sigmodontis*, *O. lupi*, and *S. labiatopapillosa*, BRAKER (Gabriel et al. 2024) (version 3) was run on soft-masked genomes using only protein alignments from related species as evidence by using nematoda OrthoDB (Kuznetsov et al. 2023) (version 11), which is comprised of 147,547 protein sequences.

Enrichment for overlap of NUWTs with different repeat categories was calculated using regioneR (Gel et al. 2016) (version 3.20), which performs a permutation test (10,000 times) while maintaining chromosome structure. Results were afterward corrected for multiple testing with Benjamini–Hochberg.

## Results

### Nematode genome phylogeny

**Fig. 1.**
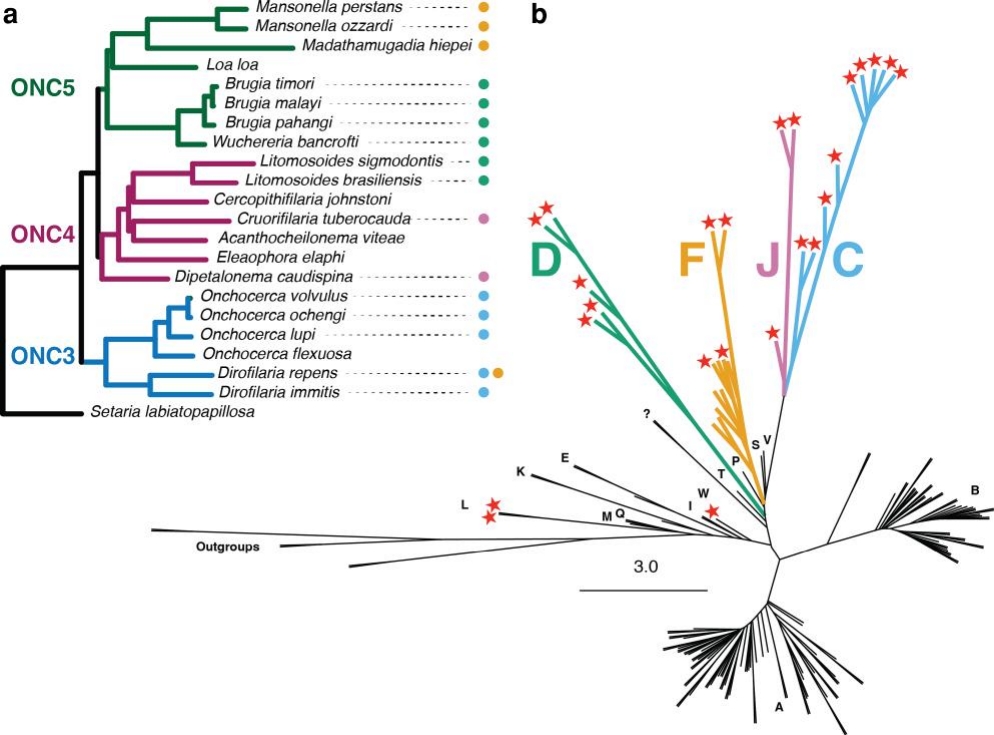
Genome phylogeny of filarial nematodes and *Wolbachia*. a) Phylogeny of 22 filarial nematodes (Onchocercidae) based on a concatenated alignment of 670 nuclear-encoded, orthologous proteins, analyzed with IQ-TREE. All nodes gained maximal support (ML bootstrap = 100%). The Onchocercinae subtaxa defined by LeFoulon et al. (Lefoulon et al. 2015) are indicated (ONC3, ONC4, ONC5). For each nematode species, current infection with *Wolbachia* is shown by a colored dot, and the supergroup allocation of the endosymbiont is indicated by colors as in part B. Scale bar is amino acid substitutions per site. b) Unrooted phylogeny of 167 *Wolbachia* genomes based on analysis of 696 orthologous proteins with IQ-TREE. Letters A-W indicate assigned supergroups. Stars indicate *Wolbachia* from nematode hosts, colored as in part A. Red stars indicate *Wolbachia* from nematodes not figured in part A. Support for deep branching between supergroups, and within supergroups C, D, F, and J, is near-maximal. The scale bar is nucleotide substitutions per site.

We collated genome data from 22 onchocercid nematode species (Table 1; Supplementary Table 1). We present new whole genome assemblies for *S. labiatopapillosa*, a filarial parasite of cattle that is an outgroup to the other species sampled, and *A. viteae*, a rodent parasite. We also generated additional data for *Dirofilaria (Dr.) repens*. We annotated the genomes and identified 670 single-copy orthologues. Using these we re-estimated the relationships of the Onchocercidae. The phylogeny is fully resolved with maximal support for every node (Fig. 1a). Rooting the phylogeny with *S. labiatopapillosa*, we recapitulate the phylogeny produced by Lefoulon et al. (Lefoulon et al. 2015) based on 7 loci. We included representatives of four of the 5 clades of Onchocercidae analyzed by Lefoulon et al., lacking only a representative of their ONC1 group, which arises basally to *S. labiatopapillosa*. *Elaeophora elaphi* was not included in the analyses of Lefoulon et al., but its position, as sister to *A. viteae* and *Litomosoides (Li.) sigmodontis*, is as expected (Hernández Rodríguez et al. 1986).

### *Wolbachia* genomes

We used BlobToolKit (Challis et al. 2020) to identify *Wolbachia* symbiont genomes in newly sequenced nematode genomes. No symbiont genomes were detected in *S. labiatopapillosa* or *A. viteae*. In whole genome data from *Dr. repens*, we identified the presence of 2 distinct *Wolbachia*. One *Wolbachia* was from the expected supergroup C. The other, larger genome had distinct coverage and GC content and was placed in supergroup F (Fig. 1b; Supplementary Fig. 1). Both *Wolbachia* assemblies were deemed to be of high quality based on the Rickettsiales dataset within CheckM, at 98.1% complete (supergroup C genome) and 97.6% complete (supergroup F genome).

We collated genome data from 1,444 isolates of *Wolbachia* (Supplementary Table 2), from 242 host species, including *Wolbachia* genomes for all the *Wolbachia*-infected nematodes analyzed in this study (apart from *Brugia timori* and *Onchocerca lupi*, where *Wolbachia* assemblies were not available). To avoid potential biases due to different annotation protocols for published *Wolbachia* genomes, we repredicted proteomes for all strains with prokka/prodigal (Seemann 2014). Using orthoFinder (Emms and Kelly 2015), we generated an orthology clustering for the 1,444 newly predicted proteomes from 16 different supergroups (A-F, I-M, P, S, T, V, W) and 7 outgroup species. A large fraction of the orthogroups was singletons (6,659 proteins) or strain-specific orthogroups (144 orthogroups).

The majority (97.5%) of the 1,667,683 proteins were clustered into 1,386 multi-genome orthogroups. Only 432 orthogroups had members from all 16 *Wolbachia* supergroups, but 725 had representatives from all of the well-represented supergroups A-F. A large set of 2,386 orthogroups was found to be specific to a *Wolbachia* supergroup. Most of these were specific to supergroup D (841), followed by A (519), C (316), B (265), K (197), F (88), M (52), E (44), V (28), I (20), L (9), and J (7). Two hundred families were only present in supergroups A and B but were missing in all others and are mainly composed of transposases.

Rarefaction analyses, using only genomes with high completeness, showed major differences between *Wolbachia* supergroups in terms of their predicted size of their pan-proteome, the total protein diversity likely to be present in all strains of each supergroup. For *Wolbachia* as a whole, the rarefaction curve levels off after addition of the proteomes of about 30 strains, suggesting that the proteome diversity of *Wolbachia* likely includes 3,000 orthogroups (Supplementary Fig. 3). Within supergroups A and B, diversity appeared to asymptote at approximately 2,200 orthogroups after the addition of around 30 proteomes (Supplementary Fig. 3a). The C, D and F pan-proteomes did not reach asymptote, likely because too few proteomes were available, but indicated pan-proteome sizes of 1,300 orthogroups in supergroup F, 1,370 orthogroups in supergroup C and 1,290 orthogroups in supergroup D (Supplementary Fig. 3b).

To reduce redundancy across the *Wolbachia* genomes, a set of 165 strains retaining maximal sequence diversity in the dataset was selected using the dRep toolkit (Olm et al. 2017) (Supplementary Table 2). We derived a robust *Wolbachia* phylogeny using a concatenated protein supermatrix derived from 696 one-to-one orthologues present in at least 75% of the selected strains (Fig. 1b). This phylogeny supported the distinctiveness of the different supergroups. Between-strain divergences within supergroups A and B were relatively small compared to the divergence observed within supergroups C, D, J, and, to a lesser degree, F. The supergroup topology was similar to previously described phylogenies. The nematode-infecting *Wolbachia* were interspersed with minor arthropod-infecting clades. The nematode-infecting supergroups C and J were sisters. Supergroup F is sister to a clade comprising supergroups C, J, S, and V, and supergroup D is sister to a clade of C, J, V, S, P, and S *Wolbachia*. This structure reflects that described previously (Lefoulon et al. 2020c; Dudzic et al. 2022; Vancaester and Blaxter 2023). The supergroup F *Wolbachia* infecting *Dr. repens* had a slightly unexpected position within the supergroup, as it clustered closest to genomes of the insect-infecting supergroup F *Wolbachia* rather than other nematode-infecting supergroup F genomes (Fig. 1b). A closely related supergroup F strain in the nematode *Cercopithifilaria (Ce.) japonica* was recovered among *Wolbachia* infecting the bed bug *Cimex lectularius* and termites from the genus *Coptotermes* and *Odontotermes* (Lefoulon et al. 2016; Sinha et al. 2023b).

### NUWTs in 22 filarial nematode genomes

NUWTs are an instructive marker of historical association between a nuclear genome and *Wolbachia* (Koutsovoulos et al. 2014). Even if *Wolbachia* infection has been cleared, NUWTs can persist as genomic fossils in the nuclear genome. As the vast majority of NUWTs are nonfunctional in their new host genome (Blaxter 2007), they evolve neutrally with no constraint on small insertions and deletions and thus can be hard to detect by simple sequence similarity search. We used our *Wolbachia* protein orthogroups to develop tools to identify NUWTs in the filarial nematode nuclear genomes. Briefly, we used the orthoFinder protein clustering to identify families with more than 3 members. For each family we aligned the proteins, back-translated these to generate a nucleotide alignment and turned this alignment into a HMM. We used the HMM library to screen the nematode genome sequences and collected all hits with likelihoods less than 1e-30. These hits were aligned to the nucleotide alignments of the HMMs, and phylogenetic trees inferred for each gene family and its putative NUWTs. The phylogenetic trees were then screened to remove hits that likely corresponded to resident nematode genes (revealed by subtrees containing all or most nematode species placed distinctly from any *Wolbachia* sequence). The other hits, which we accept as NUWTs, were classified to likely supergroup of origin based on their phylogenetic placement (see Supplementary Figs. 4–8).

For each of the 2,647 *Wolbachia* ortholog clusters containing more than 3 members across 3 different strains, we generated a protein alignment (Katoh and Standley 2013) and back-translated each alignment into the original DNA sequences (Rice et al. 2000). While direct sequence- or sequence profile-based searches are sensitive to changes in frame that result from insertion and deletion mutations, HMMs are able to identify similar sequences even in the presence of insertions and deletions (Durbin et al. 1998). We derived a HMM from each DNA alignment and used these HMMs to screen the nematode nuclear genomes for NUWTs (Eddy 2011). Almost half of the HMMs (1,179, 46%) identified putative NUWTs in the filarial genomes at an E-value cutoff of 1e-30. Using IQ-TREE (Minh et al. 2020), we generated phylogenetic trees of the *Wolbachia* genes and putative NUWT sequences from all 1,179 clusters that had putative NUWTs. These phylogenies were rooted and then parsed to discriminate between true NUWTs, with unequivocal signal of similarity to *Wolbachia* genes, and nematode genomic fragments identified due to a lack of specificity in the HMMs.

We identified a total of 7,906 initial matches grouped in just over 5,000 regions with between 12 matches in 12 regions (*S. labiatopapillosa*) and 818 matches in 653 regions (*Onchocerca volvulus*) in each nematode genome (see Supplementary Table 4). For most matches, the filarial nematode nuclear genome-derived sequences nested within the diversity of sequences from *Wolbachia* (see Supplementary Figs. 4–8 for examples of these phylogenies). The 12 HMM hits to 7 orthologous families identified in *S. labiatopapillosa* all had higher sequence similarity to sequences from the other filarial species analyzed and to other nonfilarial nematode species than to *Wolbachia* sequences and thus likely derive from false-positive, off-target matches to nematode nuclear sequences. We interpret these data as indicating that *S. labiatopapillosa* has no evidence of current or ancient infection with *Wolbachia* (Lefoulon et al. 2015, 2016).

This low level of false positives gives us confidence in interpreting NUWTs identified in the nuclear genomes of other *Wolbachia*-negative filaria. It may be that our ability to identify NUWTs is negatively impacted by low genome quality or over-purging of fragments that have similarity to *Wolbachia*. However, we found similar patterns of NUWT abundance in closely related species despite the differences in assembly technologies and contiguity. The impact of NUWTs not observed because of technical issues will be minimal, and at worst we will have missed true associations rather than inferred false associations.

### Congruence and incongruence in the origins of NUWTs

Placing the supergroup origin of live *Wolbachia* infections on the nematode phylogeny, it is evident that the current pattern of presence across the filaria requires multiple infections with symbionts, including at least 2 distinct instances of infection with supergroup J and supergroup D strains and 5 independent losses of infection (Fig. 1a). Exploration of the distribution of NUWTs indicates that the hidden history of association between *Wolbachia* and filaria is even more complex (Fig. 3).

**Fig. 2.**
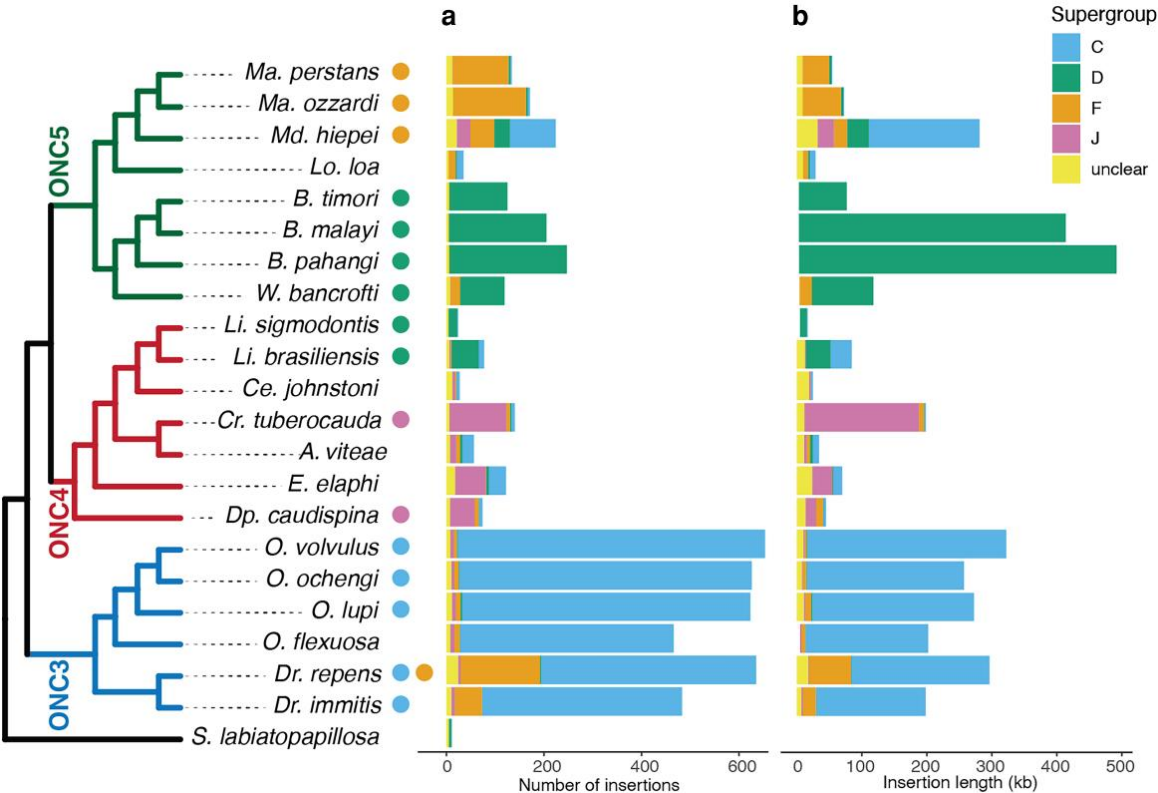
NUWTs in filarial nematode genomes. a) For each nematode species tested, the stacked histogram shows the classification by supergroup of origin of the NUWTs identified by HMM searching. NUWTs that had no clear phylogenetic association with any sequence from live *Wolbachia* are labeled “unclear.” Data are available in Supplementary Table 3. b) The sum of the lengths of the *Wolbachia* insertions for each species, color coded by supergroup of origin. The cladogram to the left shows the relationships of the nematodes (from Fig. 1a), and the colored dots show the current infections of each species.

Several patterns were evident in the frequency and origins of NUWTs in onchocercid genomes. Firstly, nematodes infected with supergroup C *Wolbachia* had more NUWTs than those infected with supergroup D (Fig. 2). In *Dirofilaria* and *Onchocerca* species carrying live supergroup C *Wolbachia* (i.e. *Dr. immitis, Dr. repens, O. volvulus*, *O. ochengi*, and *O. lupi*), we found between 483 and 653 NUWT regions, while in *Brugia* species, *W. bancrofti,* and *Litomosoides* species infected with supergroup D *Wolbachia*, there were between 24 and 247. However, the inserted regions in *Brugia* species were on average larger. NUWTs of D origin in *Brugia* species were on average 2,038 bp, while NUWTs of C origin in *Onchocerca* species were on average 434 bp. The total inserted NUWT length was more than 400 kb in *Brugia* species, but only 188 to 306 kb in *Onchocerca* species (Fig. 2b). The longest NUWT region detected, in *Brugia malayi*, was 29 kb.

The NUWTs found in a species with a live *Wolbachia* infection were largely identified as having come from the same supergroup as the live infection. For example, 96% of the NUWTs in *O. volvulus* were derived from supergroup C *Wolbachia*. The majority of the remainder was assigned to supergroups F and J (1% each). Whether these non-C classifications are real or due to unavoidable phylogenetic noise, perhaps due to short sequences evolving neutrally, is unclear. This pattern was especially strong in *Brugia* species, with 95% to 98% of NUWTs deriving from supergroup D *Wolbachia*, and the remainder largely unclassified. NUWTs derived from supergroup C genomes were observed in all filarial nematodes except for the *Brugia* and *Wuchereria* clade.

The *Wolbachia*-free nematodes *A. viteae, Ce. johnstoni, E. elaphi*, *Lo. loa*, and *O. flexuosa* all carried NUWTs, attesting to their having once been infected. NUWTs have been described previously in *O. flexuosa* (Fenn et al. 2006; McNulty et al. 2013) and *Lo. loa* (Desjardins et al. 2013). These filarial nematode species that have lost their *Wolbachia* infections (i.e. are aposymbiotic) mostly had fewer insertions with lower span than related species that have retained *Wolbachia* infection. We identified only 466 NUWTs in *O. flexuosa* compared to the >600 found in the other *Onchocerca* spp. As the ancestor of *O. flexuosa* that eliminated its *Wolbachia* infection might be expected to have had the same number of NUWTs as other *Onchocerca* do today, this suggests that *O. flexuosa* has been losing NUWTs since it became aposymbiotic or that other *Onchocerca* species have had longer to accumulate NUWTs. The *Wolbachia*-free *E. elaphi* had more NUWT regions (122) than the closely related, *Dipetalonema (Dp.) caudispina*, which is infected by a supergroup J *Wolbachia* (74). *A. viteae, Ce. Johnstoni*, and *Lo. loa* had only 56, 27, and 35 NUWTs, respectively. If NUWTs are lost and gained in a clock-like fashion, this implies that *A. viteae*, *Ce. johnstoni*, and *Lo. loa* lost their *Wolbachia* earlier than did *E. elaphi*. The NUWTs carried by *Wolbachia*-free nematodes were frequently derived from different supergroups than currently found infecting their closest relatives. For example, *Lo. loa* is sister to the supergroup F *Wolbachia*-infected *Mansonella-Madathaumugadia* clade in ONC5 but carries both supergroup F and supergroup C NUWTs. Similarly, *A. viteae* and *E. elaphi* carry supergroup C-derived NUWTs as well as the supergroup J NUWTs expected from supergroup J infection of their phylogenetic neighbors *Dp. caudispina* and *Cr. tubercauda*.

### Dynamics of NUWT evolution show *Wolbachia* superinfection and replacement

In *Dr. repens*, there were 635 NUWT regions, 69% from C and 26% from F sources. Given the identification of a supergroup F *Wolbachia* in one of the 2 isolates sequenced, we interpret this difference in frequency as having arisen from the continuous presence of C *Wolbachia* in the species, and lower rates or duration of infection with F *Wolbachia*. In *Dr. immitis*, while most (85%) of the 483 NUWTs were derived from supergroup C *Wolbachia*, 58 (12%) were derived from supergroup F. For example, *Dr. immitis* carried 2 C-derived and 1 F-derived NUWTs derived from horizontal transfer of orthogroup OG0000236 (dihydrolipoyl dehydrogenase) genes, which also identified *Onchocerca* C-derived and *Md. hiepei* and *Dp. caudispinia* J-derived NUWTs (Supplementary Fig. 4). This suggests that *Dr. immitis*, or perhaps the common ancestor of the genus *Dirofilaria*, was once infected by a supergroup F strain or that the species is currently variably infected by an F strain that has not yet been sampled. In this context, the F-like NUWTs in *O. volvulus* are intriguing as they may point to ancient or current rare infection with an F supergroup *Wolbachia*. We did not find any F supergroup NUWT regions that were likely to be orthologous between *Onchocerca* and *Dirofilaria*.

We found evidence for repeated *Wolbachia* replacement in the genome of *Md. hiepei.* Significant fractions of *Md. heipei* NUWTs were classified as deriving from all of the filarial nematode-infecting supergroups (42% from supergroup C, 22% from F, 14% from D and 12.5% from J). Fifty-eight out of the 349 orthogroups that had *Md. heipei* NUWTs contained multiple sequences from *Md. heipei* that likely originated from different *Wolbachia* supergroups. Eight contained NUWTs assigned to 3 different supergroups, e.g. OG0000277 (peptide deformylase; Supplementary Fig. 5).

We identified many cases of sets of NUWTs that clustered in phylogenetic trees and recapitulated the phylogenies of their hosts. Most of these groups of related NUWTs were found in the four closely related *Onchocerca* species or the 3 *Brugia* species. Some supergroup C-derived NUWT groups included all *Onchocerca* members, suggesting they were inserted in the last common ancestor of the *Onchocerca* clade (e.g. OG0000301; Supplementary Fig. 6). Similar patterns were observed for many D supergroup NUWTs in *Brugia* species (e.g. OG0000453; Supplementary Fig. 7). As previously noted in the strongyloidean nematode *Dictyocaulus viviparus*, NUWTs can be duplicated within their host genomes (Koutsovoulos et al. 2014). We identified several NUWT groups that had multiple copies in their host genomes, particularly in the genomes of *Brugia* spp. (e.g. ATP-dependent zinc metalloprotease FtsH OG0000206; Supplementary Fig. 8).

We explored whether NUWT integration in the genome is biased toward regions enriched in certain features. We overlaid repeat annotation with NUWT integration sites to detect enrichment toward certain repetitive classes and detected strong enrichment for the long terminal repeats from the family Pao. This was significant in all *Brugia* and *Dirofilaria* genomes, *O. volvulus*, *O. flexuosa*, *A. viteae*, *Cr. tuberocauda*, *W. bancrofti*, *Dp. Caudispina*, and *E. elaphi*. Furthermore, we found a strong regional association between helitron density and NUWT integration specific to the genus *Onchocerca*, *O. volvulus*, *O. flexuosa*, *O. lupi*, and *O. ochengi.* No association with repeats was identified in *Md. hiepei*, the 2 *Mansonella* species, *Ce. johnstoni*, *O. ochengi*, and *O. lupi*.

## Discussion

We used nucleotide HMM built from genes present in living *Wolbachia* to detect fossils of *Wolbachia* sequence inserted into the genomes of filarial nematodes. This NUWT identification pipeline appears to be an honest estimator of association between nematode nuclear genomes and *Wolbachia* symbionts. The pipeline will not detect NUWTs that derive from *Wolbachia* genes that were singletons in our orthology clustering or from sequences that are not part of protein-coding genes. It will also fail to distinguish *Wolbachia*-derived sequence from other nematode noncoding sequence when sufficient time and thus mutational change has happened. The pipeline also relies on the assumption that the HMM are specific to *Wolbachia* genes and that spurious matches to nuclear genome sequences are not inflating the count of hits. Based on its phylogenetic position, *S. labiatopapillosa* is believed not to have had an association with *Wolbachia* (Lefoulon et al. 2015, 2016). The HMM hits identified in *S. labiatopapillosa* were short and grouped with other likely nematode nuclear genome-resident sequences. We thus believe that the method generates few false positives. Most of the nematode genomes are incomplete, and the missing data could include *Wolbachia* insertions. Overall, our method conservatively underestimates the number of true NUWTs. Analysis of the pattern of likely origin of the NUWTs across the filarial nematode phylogeny suggests that *Wolbachia* infection in this group is characterized by persistent infection modified by frequent loss and replacement, a pattern at odds with stable, mutualist models.

The lack of NUWTs in *S. labiatopapillosa* is in keeping with the model of LeFoulon et al. (Lefoulon et al. 2015, 2016) where the relationship between *Wolbachia* and filarial nematodes was first established in the ancestor of groups ONC3 (*Dirofilaria* and *Onchocerca*), ONC4 (*Dipetalonema*, *Acanthocheilonema*, *Cruorifilaria*, *Cercopithifilaria*, and *Litomosoides*) and ONC5 (*Wuchereria*, *Brugia*, *Loa*, *Madathamugadia*, and *Mansonella*) (Fig. 1a). This parasitic group has been estimated to have diverged at the start of the Paleogene around 60 million years ago, a period of rapid mammal diversification (Qing et al. 2025).

**Fig. 3.**
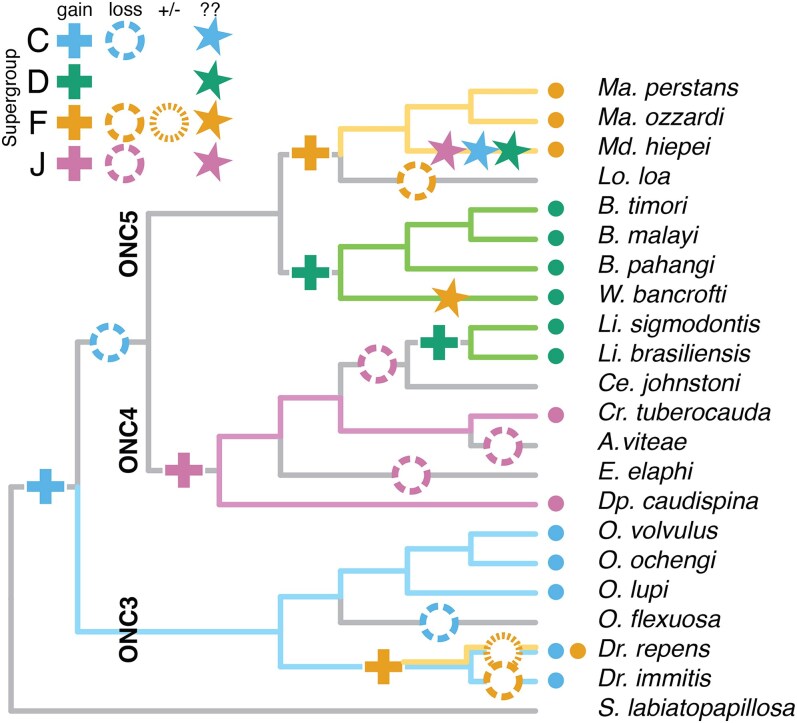
Origins of NUWTs in symbiotic and aposymbiotic filarial nematodes. Hypothesis of *Wolbachia* association through filarial nematode evolution. The cladogram shows the relationships of the nematodes (from Fig. 1a), and the colored dots show the current infections of each species. Plus signs indicate acquisition of *Wolbachia*, with the supergroup indicated by color. “X” in a coarse-dashed circles indicates loss. The variably present supergroup F *Wolbachia* in *D. repens* is shown by “±” in a fine-dashed circle. “?” indicates species with an unexpected frequency of NUWTs from *Wolbachia* supergroups that may indicate hit-and-run associations.

The subsequent history of *Wolbachia* infection in filarial nematodes is complex, and here we propose a model for this evolving association (Fig. 3). Almost all of the analyzed genomes, even those from species that lack current live infection with *Wolbachia*, have supergroup C NUWTs. This suggests that the original infection was with a supergroup C (or C-like) *Wolbachia*, which left NUWTs in the genomes of all descendant species (Fig. 3). All analyzed species in ONC3 that have a live infection with a supergroup C *Wolbachia* have a high level of supergroup C NUWTs. *O. flexuosa* has secondarily lost its *Wolbachia* infection (McNulty et al. 2012) and has a lower load of supergroup C insertions than its close sister species, possibly because of ongoing stochastic loss and/or sequence drift. Many NUWTs in the genomes of *Onchocerca* species were orthologous (Supplementary Fig. 6) and must have originated from integration in the genome of a common ancestor.

All genomes of nematode group ONC3 have NUWTs derived from supergroup F *Wolbachia*. Both *Dirofilaria* genomes have an elevated number of insertions of supergroup F-derived NUWTs (12 to 25% of all NUWTs). We found that *Dr. repens* can be simultaneously coinfected with supergroup C and supergroup F *Wolbachia*. While coinfection, especially involving supergroup A and B strains, is quite common across arthropod hosts (Ellegaard et al. 2013; Funkhouser-Jones et al. 2015; da Silva et al. 2024), this is to the best of our knowledge the first demonstration of dual infection in nematodes. The potential for dual infection is supported by the demonstration, based on PCR amplicons of bacterial small subunit RNA loci (16S) in specimens of the related species *Dirofilaria hongkongensis*, of supergroup C and supergroup F *Wolbachia* infection (Sarasombath et al. 2024). We suggest that the dual infection observed in *Dr. repens* is likely to have been present in the common ancestor of *Dr. immitis*, *Dr. repens*, and *Dr. hongkongensis*, and this was the source of the frequent supergroup F NUWTs in *Dr. immitis*. Supergroup F infection has either been secondarily lost in *Dr. immitis* or is present in an as-yet-unsampled subset of individuals of this species (Fig. 3).

NUWTs assigned to a supergroup F source were identified in nearly all genomes, with only *E. elaphi* and *Li. sigmodontis* having none. While it is possible that assignment of some NUWTs to supergroup F is the result of noise in the phylogenetic reconstructions, in many cases the allocation of the NUWT to supergroup F was strongly supported. Supergroup F *Wolbachia* are remarkable in being the only supergroup known to infect both arthropods and nematodes, and phylogenetic analysis of F genomes has shown that there are 2 distinct lineages of nematode-infecting F *Wolbachia* (Sinha et al. 2023b). The newly sequenced F genome from *Dr. repens* clustered together with the supergroup F genomes from *Mansonella*, suggesting limited cophylogeny with filarial nematodes. It is striking that NUWTs in the strongyle nematode *Dictyocaulus viviparus* were attributable to a supergroup F *Wolbachia*, despite no occurrence of a live infection in this or any other strongyle nematode being identified to date (Koutsovoulos et al. 2014). The lungworms (Strongyloidea) are not closely related to filarial nematodes and are estimated to have diverged 300 to 400 million years ago (Qing et al. 2025). If the NUWTs in *D. viviparus* are the result of a “hit-and-run” infection of limited phylogenetic perdurance, the scattering of supergroup F NUWTs across the filaria could also result from similar brief “hit-and-run” infections with supergroup F *Wolbachia*.

NUWTs derived from supergroup D were common in *Brugia* and *W. bancrofti* in ONC5 and in *Litomosoides* species in ONC4. This pattern suggests 2 independent acquisitions of the supergroup D *Wolbachia*. The supergroup D NUWTs in *Brugia* were the longest detected in this analysis, with both the longest NUWT and the longest average NUWT size (Fig. 2b). These may reflect a burst of recent insertions that have not yet been fragmented by neutral genomic processes. The other clade in ONC5, containing *Mansonella* species, *Md. Heipei*, and *Lo. loa*, has been infected by supergroup F *Wolbachia*. *Md. heipei* is currently infected by a supergroup F *Wolbachia* but carries significant numbers of NUWTs for supergroups C (42% of NUWTs), D (14%), F (22%), and J (12%). This atypical NUWT distribution (Fig. 2) suggests that this species or its ancestors has experienced multiple “hit-and-run” infections in its relatively recent evolutionary history.

The phylogenetic relationships of nematodes in ONC4 suggest that an ancestral species was infected with supergroup J *Wolbachia*, retained in *Cr. tubercauda* and *Dp. caudispina*. This *Wolbachia* has been lost in *A. viteae* and *E. elaphi* and has subsequently been replaced by a supergroup D *Wolbachia* in the *Litomosoides* species. In both *Li. sigmodontis* and *Li. Brasiliensis*, we found NUWTs derived from supergroup C *Wolbachia* (2 and 11 regions, respectively), and in *Li. brasiliensis* these accounted for about one-third of the span of the NUWTs identified. These supergroup C-derived NUWTs may point to historical infection by a C *Wolbachia* in these species or a *Litomosoides* ancestor.

NUWT analysis allows us to assess the wider evolutionary history of interaction between *Wolbachia* and its hosts. It was originally proposed that *Wolbachia* show strong cophylogeny with their filarial nematode hosts with exclusively vertical transmission (Bandi et al. 1998). This pattern contrasted with the biology of supergroups A and B where host-switching events among arthropod hosts are rampant (Vancaester and Blaxter 2023). We find that cophylogeny is in fact relatively limited in these nematode symbionts and that instead events of host switching, where an incoming strain replaces a resident one, and perhaps frequent hit-and-run and dual infections, generate a very complex set of associations. The dynamic nature of the association between *Wolbachia* and filarial nematodes we infer here implies that there is unlikely to be a single, mutualist explanation for the association (Fenn and Blaxter 2004), and it remains open whether the associations are supportive (through metabolic supplementation or through manipulation of vector or mammalian host immunity), parasitic (for example, reproductive manipulation as observed in A and B supergroup *Wolbachia* in arthropods), or “ransom” or “addiction” based (where loss of live *Wolbachia* in germline cells results in toxin-mediated necrosis (Foray et al. 2018; Mills et al. 2023), and thus impacts host fitness (Sullivan 2017)). The frequent presence of *Wolbachia* in filarial nematodes and the lack of infection in most other nematode groups may be due to chance, a particular genetic susceptibility to infection in filaria, or these parasites' intimate relationship with *Wolbachia*-infected arthropods. This susceptibility may in turn have led to filarial nematodes being a fertile evolutionary playground for competing *Wolbachia* supergroup lineages.

We discovered horizontal transfers of DNA between symbionts and their hosts through a library of nucleotide HMMs representing symbiont genes that were able to identify transferred fragments, even if they had been subject to substitution, insertion, and deletion. This HMM approach is equally applicable to finding NUWTs in other host groups and, given the building of HMM libraries for genes from representative symbiont genomes, insertions derived from other prokaryotic and eukaryotic symbiont taxa. We expect that, as in this nematode-*Wolbachia* example, hidden histories of symbiosis will emerge from deployment of such tools.

## Supplementary Material

jkaf226_Supplementary_Data

## Data Availability

New nematode genome sequences and raw data are available in INSDC under BioProjects PRJEB7555 (*S. labiatopapillosa*) and PRJEB1697 (*A. viteae*) and new *Wolbachia* genome sequences are available in INSDC under BioProject PRJEB95945. A list of *Wolbachia* genome sequences analyzed is available in Supplementary Tables 2 and 3. The Supplementary Data, Figures, and Tables have been uploaded to Zenodo under doi 10.5281/zenodo.16833941. Supplemental material available at G3 online.

## References

[jkaf226-B1] Altschul SF, Gish W, Miller W, Myers EW, Lipman DJ. 1990. Basic local alignment search tool. J Mol Biol. 215:403–410. 10.1016/S0022-2836(05)80360-2. 2231712

[jkaf226-B2] Baltazard M, Chabaud AG, Minou A. 1952. Evolutionary cycle of a filarial parasite of the jird. Comp Rendus Seances Acad Sci Paris. 234:2115–2117. 12979339

[jkaf226-B3] Bandi C, Anderson T, Genchi C, Blaxter ML. 1998. Phylogeny of Wolbachia in filarial nematodes. Proc R Soc Lond B Biol Sci. 265:2407–2413. 10.1098/rspb.1998.0591. PMC1689538 9921679

[jkaf226-B4] Bankevich A et al 2012. SPAdes: a new genome assembly algorithm and its applications to single-cell sequencing. J Comput Biol. 19:455–477. 10.1089/cmb.2012.0021. 22506599 PMC3342519

[jkaf226-B5] Baril T, Galbraith J, Hayward A. 2024. Earl grey: a fully automated user-friendly transposable element annotation and analysis pipeline. Mol Biol Evol. 41:msae068. 10.1093/molbev/msae068. 38577785 PMC11003543

[jkaf226-B6] Beliavskaia A et al 2023. Metagenomics of culture isolates and insect tissue illuminate the evolution of, and symbionts in spp. Fleas. Microb Genom. 9:mgen001045. 10.1099/mgen.0.001045. 37399133 PMC10438800

[jkaf226-B7] Bennett GM, Moran NA. 2015. Heritable symbiosis: the advantages and perils of an evolutionary rabbit hole. Proc Natl Acad Sci U S A. 112:10169–10176. 10.1073/pnas.1421388112. 25713367 PMC4547261

[jkaf226-B8] Blaxter M . 2007. Symbiont genes in host genomes: fragments with a future? Cell Host Microbe. 2:211–213. 10.1016/j.chom.2007.09.008. 18005738

[jkaf226-B9] Bouchery T, Lefoulon E, Karadjian G, Nieguitsila A, Martin C. 2013. The symbiotic role of Wolbachia in onchocercidae and its impact on filariasis. Clin Microbiol Infect. 19:131–140. 10.1111/1469-0691.12069. 23398406

[jkaf226-B10] Brown AMV et al 2018. Comparative genomics of Wolbachia-Cardinium dual endosymbiosis in a plant-parasitic nematode. Front Microbiol. 9:2482. 10.3389/fmicb.2018.02482. 30459726 PMC6232779

[jkaf226-B11] Cafarelli C, Russo G, Mathis A, Silaghi C. 2019. De novo genome sequencing and comparative stage-specific transcriptomic analysis of dirofilaria repens. Int J Parasitol. 49:911–919. 10.1016/j.ijpara.2019.04.008. 31557466

[jkaf226-B12] Capella-Gutierrez S, Silla-Martinez JM, Gabaldon T. 2009. Trimal: a tool for automated alignment trimming in large-scale phylogenetic analyses. Bioinformatics. 25:1972–1973. 10.1093/bioinformatics/btp348. 19505945 PMC2712344

[jkaf226-B13] Caragata EP, Dutra HLC, Moreira LA. 2016. Exploiting intimate relationships: controlling mosquito-transmitted disease with Wolbachia. Trends Parasitol. 32:207–218. 10.1016/j.pt.2015.10.011. 26776329

[jkaf226-B14] Challis R, Richards E, Rajan J, Cochrane G, Blaxter M. 2020. BlobToolKit—interactive quality assessment of genome assemblies. G3 (Bethesda). 10:1361–1374. 10.1534/g3.119.400908. 32071071 PMC7144090

[jkaf226-B15] Chen S, Zhou Y, Chen Y, Gu J. 2018. Fastp: an ultra-fast all-in-one FASTQ preprocessor. Bioinformatics. 34:i884–i890. 10.1093/bioinformatics/bty560. 30423086 PMC6129281

[jkaf226-B16] Chung M, Small ST, Serre D, Zimmerman PA, Dunning Hotopp JC. 2017. Draft genome sequence of the Wolbachia endosymbiont of wuchereria bancrofti wWb. Pathog Dis. 75:ftx115. 10.1093/femspd/ftx115. 29099918 PMC5827699

[jkaf226-B17] Costa CHA et al 2023. Ribosomal, mitochondrial and bacterial (Wolbachia) reference sequences for dipetalonema gracile. Acta Amazon. 53:130–140. 10.1590/1809-4392202201741.

[jkaf226-B18] Cotton JA et al 2017. The genome of onchocerca volvulus, agent of river blindness. Nat Microbiol. 2:16216. 10.1038/nmicrobiol.2016.216. PMC5310847 27869790

[jkaf226-B19] Darby AC et al 2014. Integrated transcriptomic and proteomic analysis of the global response of *Wolbachia* to doxycycline-induced stress. ISME J. 8:925–937. 10.1038/ismej.2013.192. 24152719 PMC3960535

[jkaf226-B20] da Silva LMI et al 2024. Sequencing and analysis of strains from A and B supergroups detected in sylvatic mosquitoes from Brazil. Microorganisms. 12:2206. 10.3390/microorganisms12112206. 39597595 PMC11596719

[jkaf226-B21] Desjardins CA et al 2013. Genomics of loa loa, a Wolbachia-free filarial parasite of humans. Nat Genet. 45:495–500. 10.1038/ng.2585. 23525074 PMC4238225

[jkaf226-B22] Doudoumis V et al 2012. Detection and characterization of Wolbachia infections in laboratory and natural populations of different species of tsetse flies (genus glossina). BMC Microbiol. 12:S3. 10.1186/1471-2180-12-S1-S3. 22376025 PMC3287514

[jkaf226-B23] Dudzic JP, Curtis CI, Gowen BE, Perlman SJ. 2022. A highly divergent *Wolbachia* with a tiny genome in an insect-parasitic tylenchid nematode. Proc Biol Sci. 289:20221518. 10.1098/rspb.2022.1518. 36168763 PMC9515626

[jkaf226-B24] Dunning-Hotopp JC et al 2007. Widespread lateral gene transfer from intracellular bacteria to multicellular eukaryotes. Science. 317:1753–1756. 10.1126/science.1142490. 17761848

[jkaf226-B25] Durbin R, Eddy S, Krogh A, Mitchison G. 1998. Biological sequence analysis. Probabilistic models of proteins and nucleic acids. Cambridge Univerity Press.

[jkaf226-B26] Eddy SR . 2011. Accelerated profile HMM searches. PLoS Comput Biol. 7:e1002195. 10.1371/journal.pcbi.1002195. 22039361 PMC3197634

[jkaf226-B27] Ehrens A, Hoerauf A, Hübner MP. 2022. Current perspective of new anti-wolbachial and direct-acting macrofilaricidal drugs as treatment strategies for human filariasis. GMS Infect Dis. 10:Doc02. 10.3205/id000079. 35463816 PMC9006451

[jkaf226-B28] Ellegaard KM, Klasson L, Näslund K, Bourtzis K, Andersson SGE. 2013. Comparative genomics of Wolbachia and the bacterial species concept. PLoS Genet. 9:e1003381. 10.1371/journal.pgen.1003381. 23593012 PMC3616963

[jkaf226-B29] Emms DM, Kelly S. 2015. OrthoFinder: solving fundamental biases in whole genome comparisons dramatically improves orthogroup inference accuracy. Genome Biol. 16:157. 10.1186/s13059-015-0721-2. 26243257 PMC4531804

[jkaf226-B30] Fenn K et al 2006. Phylogenetic relationships of the Wolbachia of nematodes and arthropods. PLoS Pathog. 2:e94. 10.1371/journal.ppat.0020094. 17040125 PMC1599763

[jkaf226-B31] Fenn K, Blaxter M. 2004. Are filarial nematode Wolbachia obligate mutualist symbionts? Trends Ecol Evol. 19:163–166. 10.1016/j.tree.2004.01.002. 16701248

[jkaf226-B32] Foray V, Pérez-Jiménez MM, Fattouh N, Landmann F. 2018. Wolbachia control stem cell behavior and stimulate germline proliferation in filarial Nematodes. Dev Cell. 45:198–211.e3. 10.1016/j.devcel.2018.03.017. 29689195

[jkaf226-B33] Funkhouser-Jones LJ et al 2015. *Wolbachia* co-infection in a hybrid zone: discovery of horizontal gene transfers from two *Wolbachia* supergroups into an animal genome. PeerJ. 3:e1479. 10.7717/peerj.1479. 26664808 PMC4675112

[jkaf226-B34] Gabriel L et al 2024. BRAKER3: fully automated genome annotation using RNA-Seq and protein evidence with GeneMark-ETP, AUGUSTUS, and TSEBRA. Genome Res. 34:769–777. 10.1101/gr.278090.123. 38866550 PMC11216308

[jkaf226-B35] Gel B et al 2016. Regioner: an R/bioconductor package for the association analysis of genomic regions based on permutation tests. Bioinformatics. 32:289–291. 10.1093/bioinformatics/btv562. 26424858 PMC4708104

[jkaf226-B36] Gerth M, Gansauge M-T, Weigert A, Bleidorn C. 2014. Phylogenomic analyses uncover origin and spread of the Wolbachia pandemic. Nat Commun. 5:5117. 10.1038/ncomms6117. 25283608

[jkaf226-B37] Glowska E, Dragun-Damian A, Dabert M, Gerth M. 2015. New Wolbachia supergroups detected in quill mites (acari: syringophilidae). Infect Genet Evol. 30:140–146. 10.1016/j.meegid.2014.12.019. 25541519

[jkaf226-B38] Gomes-de-Sá S et al 2022. De Novo assembly of the dirofilaria immitis genome by long-read nanopore-based sequencing technology on an adult worm from a canine cardiopulmonary dirofilariosis case. Animals (Basel). 12:1342. 10.3390/ani12111342. 35681811 PMC9179477

[jkaf226-B39] Hedges LM, Brownlie JC, O’Neill SL, Johnson KN. 2008. *Wolbachia* and virus protection in insects. Science. 322:702. 10.1126/science.1162418. 18974344

[jkaf226-B40] Heo Y, Wu X-L, Chen D, Ma J, Hwu W-M. 2014. BLESS: bloom filter-based error correction solution for high-throughput sequencing reads. Bioinformatics. 30:1354–1362. 10.1093/bioinformatics/btu030. 24451628 PMC6365934

[jkaf226-B41] Hernández Rodríguez S, Martínez Gómez F, Gutiérrez Palomino P. 1986. *Elaeophora elaphi* n. sp. (filarioidea: onchocercidae) parasite of the red deer *(Cervus elaphus)*. with a key of species of the genus *Elaeophora*. Ann Parasitol Hum Comp. 61:457–463. 10.1051/parasite/1986614457. 3813427

[jkaf226-B42] Hickin ML, Kakumanu ML, Schal C. 2022. Effects of Wolbachia elimination and B-vitamin supplementation on bed bug development and reproduction. Sci Rep. 12:10270. 10.1038/s41598-022-14505-2. 35715692 PMC9205976

[jkaf226-B43] Hoerauf A et al 1999. Tetracycline therapy targets intracellular bacteria in the filarial nematode litomosoides sigmodontis and results in filarial infertility. J Clin Invest. 103:11–18. 10.1172/JCI4768. 9884329 PMC407866

[jkaf226-B44] Howe KL, Bolt BJ, Shafie M, Kersey P, Berriman M. 2017. WormBase ParaSite—a comprehensive resource for helminth genomics. Mol Biochem Parasitol. 215:2–10. 10.1016/j.molbiopara.2016.11.005. 27899279 PMC5486357

[jkaf226-B45] Huerta-Cepas J, Serra F, Bork P. 2016. ETE 3: reconstruction, analysis, and visualization of phylogenomic data. Mol Biol Evol. 33:1635–1638. 10.1093/molbev/msw046. 26921390 PMC4868116

[jkaf226-B46] Hyatt D et al 2010. Prodigal: prokaryotic gene recognition and translation initiation site identification. BMC Bioinformatics. 11:119. 10.1186/1471-2105-11-119. 20211023 PMC2848648

[jkaf226-B47] International Helminth Genomes Consortium . 2019. Comparative genomics of the major parasitic worms. Nat Genet. 51:163–174. 10.1038/s41588-018-0262-1. 30397333 PMC6349046

[jkaf226-B48] Ioannidis P et al 2013. Extensively duplicated and transcriptionally active recent lateral gene transfer from a bacterial Wolbachia endosymbiont to its host filarial nematode brugia malayi. BMC Genomics. 14:639. 10.1186/1471-2164-14-639. 24053607 PMC3849323

[jkaf226-B49] Kalyaanamoorthy S, Minh BQ, Wong TKF, von Haeseler A, Jermiin LS. 2017. ModelFinder: fast model selection for accurate phylogenetic estimates. Nat Methods. 14:587–589. 10.1038/nmeth.4285. 28481363 PMC5453245

[jkaf226-B50] Katoh K, Standley DM. 2013. MAFFT multiple sequence alignment software version 7: improvements in performance and usability. Mol Biol Evol. 30:772–780. 10.1093/molbev/mst010. 23329690 PMC3603318

[jkaf226-B51] Klasson L et al 2014. Extensive duplication of the Wolbachia DNA in chromosome four of Drosophila ananassae. BMC Genomics. 15:1–17. 10.1186/1471-2164-15-1097. 25496002 PMC4299567

[jkaf226-B52] Klasson L, Kambris Z, Cook PE, Walker T, Sinkins SP. 2009. Horizontal gene transfer between Wolbachia and the mosquito aedes aegypti. BMC Genomics. 10:33. 10.1186/1471-2164-10-33. 19154594 PMC2647948

[jkaf226-B53] Kondo N, Nikoh N, Ijichi N, Shimada M, Fukatsu T. 2002. Genome fragment of *Wolbachia* endosymbiont transferred to X chromosome of host insect. Proc Natl Acad Sci U S A. 99:14280–14285. 10.1073/pnas.222228199. 12386340 PMC137875

[jkaf226-B54] Koutsovoulos G, Makepeace B, Tanya VN, Blaxter M. 2014. Palaeosymbiosis revealed by genomic fossils of Wolbachia in a strongyloidean nematode. PLoS Genet. 10:e1004397. 10.1371/journal.pgen.1004397. 24901418 PMC4046930

[jkaf226-B55] Kuznetsov D et al 2023. OrthoDB v11: annotation of orthologs in the widest sampling of organismal diversity. Nucleic Acids Res. 51:D445–D451. 10.1093/nar/gkac998. 36350662 PMC9825584

[jkaf226-B56] Landmann F et al 2012. Both asymmetric mitotic segregation and cell-to-cell invasion are required for stable germline transmission of *Wolbachia* in filarial nematodes. Biol Open. 1:536–547. 10.1242/bio.2012737. 23213446 PMC3509449

[jkaf226-B57] Landmann F, Orsi GA, Loppin B, Sullivan W. 2009. Wolbachia-mediated cytoplasmic incompatibility is associated with impaired histone deposition in the male pronucleus. PLoS Pathog. 5:e1000343. 10.1371/journal.ppat.1000343. 19300496 PMC2652114

[jkaf226-B58] Landmann F, Voronin D, Sullivan W, Taylor MJ. 2011. Anti-filarial activity of antibiotic therapy is due to extensive apoptosis after Wolbachia depletion from filarial nematodes. PLoS Pathog. 7:e1002351. 10.1371/journal.ppat.1002351. 22072969 PMC3207916

[jkaf226-B59] Lebov JF et al 2020. Complete genome sequence of *w*Bp, the *Wolbachia* endosymbiont of brugia pahangi FR3. Microbiol Resour Announc. 9:e00480-20. 10.1128/MRA.00480-20. 32616636 PMC7330238

[jkaf226-B60] Lefoulon E et al 2015. Shaking the tree: multi-locus sequence typing usurps current onchocercid (filarial nematode) phylogeny. PLoS Negl Trop Dis. 9:e0004233. 10.1371/journal.pntd.0004233. 26588229 PMC4654488

[jkaf226-B61] Lefoulon E et al 2016. Breakdown of coevolution between symbiotic bacteria *Wolbachia* and their filarial hosts. PeerJ. 4:e1840. 10.7717/peerj.1840. 27069790 PMC4824920

[jkaf226-B62] Lefoulon E et al 2019. Large enriched fragment targeted sequencing (LEFT-SEQ) applied to capture of Wolbachia genomes. Sci Rep. 9:5939. 10.1038/s41598-019-42454-w. 30976027 PMC6459864

[jkaf226-B63] Lefoulon E et al 2020b. Pseudoscorpion Wolbachia symbionts: diversity and evidence for a new supergroup S. BMC Microbiol. 20:188. 10.1186/s12866-020-01863-y. 32605600 PMC7325362

[jkaf226-B64] Lefoulon E et al 2020c. Diminutive, degraded but dissimilar: genomes from filarial nematodes do not conform to a single paradigm. Microb Genom. 6:mgen000487. 10.1099/mgen.0.000487. 33295865 PMC8116671

[jkaf226-B65] Lefoulon E, Foster JM, Truchon A, Carlow CKS, Slatko BE. 2020a. The Wolbachia symbiont: here, there and everywhere. Results Probl Cell Differ. 69:423–451. 10.1007/978-3-030-51849-3_16. 33263882

[jkaf226-B66] LePage DP et al 2017. Prophage WO genes recapitulate and enhance Wolbachia-induced cytoplasmic incompatibility. Nature. 543:243–247. 10.1038/nature21391. 28241146 PMC5358093

[jkaf226-B67] Li D, Liu C-M, Luo R, Sadakane K, Lam T-W. 2015. MEGAHIT: an ultra-fast single-node solution for large and complex metagenomics assembly via succinct *de Bruijn* graph. Bioinformatics. 31:1674–1676. 10.1093/bioinformatics/btv033. 25609793

[jkaf226-B68] Lo N et al 2007. Taxonomic status of the intracellular bacterium Wolbachia pipientis. Int J Syst Evol Microbiol. 57:654–657. 10.1099/ijs.0.64515-0. 17329802

[jkaf226-B69] Lo N, Casiraghi M, Salati E, Bazzocchi C, Bandi C. 2002. How many Wolbachia supergroups exist? Mol Biol Evol. 19:341–346. 10.1093/oxfordjournals.molbev.a004087. 11861893

[jkaf226-B70] Mahmood S, Nováková E, Martinů J, Sychra O, Hypša V. 2023. Supergroup F Wolbachia with extremely reduced genome: transition to obligate insect symbionts. Microbiome. 11:22. 10.1186/s40168-023-01462-9. 36750860 PMC9903615

[jkaf226-B71] Manni M, Berkeley MR, Seppey M, Simão FA, Zdobnov EM. 2021. BUSCO update: novel and streamlined workflows along with broader and deeper phylogenetic coverage for scoring of eukaryotic, prokaryotic, and viral genomes. Mol Biol Evol. 38:4647–4654. 10.1093/molbev/msab199. 34320186 PMC8476166

[jkaf226-B72] Mattick J et al 2020. Nearly complete genome sequence of brugia pahangi FR3. Microbiol Resour Announc. 9:e00479-20. 10.1128/MRA.00479-20. 32616635 PMC7330237

[jkaf226-B73] McCann K, Grant W, Doyle SR. 2021. The genome sequence of the Australian filarial nematode, cercopithifilaria johnstoni. Wellcome Open Res. 6:259. 10.12688/wellcomeopenres.17258.2. 34796277 PMC8564745

[jkaf226-B74] McNulty SN et al 2012. Transcriptomic and proteomic analyses of a wolbachia-free filarial parasite provide evidence of trans-kingdom horizontal gene transfer. PLoS One. 7:e45777. 10.1371/journal.pone.0045777. 23049857 PMC3458923

[jkaf226-B75] McNulty SN et al 2013. Localization of Wolbachia-like gene transcripts and peptides in adult onchocerca flexuosa worms indicates tissue specific expression. Parasit Vectors. 6:2. 10.1186/1756-3305-6-2. 23281896 PMC3549793

[jkaf226-B76] Mills MK, McCabe LG, Rodrigue EM, Lechtreck KF, Starai VJ. 2023. Wbm0076, a candidate effector protein of the Wolbachia endosymbiont of brugia malayi, disrupts eukaryotic actin dynamics. PLoS Pathog. 19:e1010777. 10.1371/journal.ppat.1010777. 36800397 PMC9980815

[jkaf226-B77] Minh BQ et al 2020. IQ-TREE 2: new models and efficient methods for phylogenetic inference in the genomic era. Mol Biol Evol. 37:1530–1534. 10.1093/molbev/msaa015. 32011700 PMC7182206

[jkaf226-B78] Mirarab S et al 2014. ASTRAL: genome-scale coalescent-based species tree estimation. Bioinformatics. 30:i541–i548. 10.1093/bioinformatics/btu462. 25161245 PMC4147915

[jkaf226-B79] Nikoh N et al 2014. Evolutionary origin of insect-*Wolbachia* nutritional mutualism. Proc Natl Acad Sci U S A. 111:10257–10262. 10.1073/pnas.1409284111. 24982177 PMC4104916

[jkaf226-B80] Olm MR, Brown CT, Brooks B, Banfield JF. 2017. Drep: a tool for fast and accurate genomic comparisons that enables improved genome recovery from metagenomes through de-replication. ISME J. 11:2864–2868. 10.1038/ismej.2017.126. 28742071 PMC5702732

[jkaf226-B81] Parks DH, Imelfort M, Skennerton CT, Hugenholtz P, Tyson GW. 2015. Checkm: assessing the quality of microbial genomes recovered from isolates, single cells, and metagenomes. Genome Res. 25:1043–1055. 10.1101/gr.186072.114. 25977477 PMC4484387

[jkaf226-B82] Qing X et al 2025. Phylogenomic insights into the evolution and origin of Nematoda. Syst Biol. 74:349–358. 10.1093/sysbio/syae073. 39737664

[jkaf226-B83] Queffelec J, Postma A, Allison JD, Slippers B. 2022. Remnants of horizontal transfers of Wolbachia genes in a Wolbachia-free woodwasp. BMC Ecol Evol. 22:36. 10.1186/s12862-022-01995-x. 35346038 PMC8962096

[jkaf226-B84] Quinlan AR, Hall IM. 2010. BEDTools: a flexible suite of utilities for comparing genomic features. Bioinformatics. 26:841–842. 10.1093/bioinformatics/btq033. 20110278 PMC2832824

[jkaf226-B85] Rice P, Longden I, Bleasby A. 2000. EMBOSS: the European molecular biology open software suite. Trends Genet. 16:276–277. 10.1016/S0168-9525(00)02024-2. 10827456

[jkaf226-B86] Roberts HK . 2023. A friend or foe: genomic investigation of the nature of wolbachia endosymbiosis in filarial nematodes. PhD Thesis. LaTrobe University, Victoria, Australia. https://opal.latrobe.edu.au/ndownloader/files/41777070.

[jkaf226-B87] Rodi M et al 2023. Whole genome analysis of two sympatric human: and sp “DEUX”. Front Cell Infect Microbiol. 13:1159814. 10.3389/fcimb.2023.1159814. 37124042 PMC10145164

[jkaf226-B88] Roe CC et al 2022. LupiQuant: a real-time PCR based assay for determining host-to-parasite DNA ratios of onchocerca lupi and host Canis lupus from onchocercosis samples. PLoS One. 17:e0276916. 10.1371/journal.pone.0276916. 36409718 PMC9678315

[jkaf226-B89] Sarasombath PT et al 2024. Integrated histological and molecular analysis of filarial Species and associated Wolbachia endosymbionts in human filariasis cases presenting atypically in Thailand. Am J Trop Med Hyg. 111:829–840. 10.4269/ajtmh.24-0147. 39106844 PMC11448521

[jkaf226-B90] Schinkel M, Bousema T, van Rij RP. 2024. Tripartite interactions between viruses, parasites, and mosquitoes. Curr Opin Insect Sci. 64:101222. 10.1016/j.cois.2024.101222. 38908822

[jkaf226-B91] Scholz M et al 2020. Large scale genome reconstructions illuminate Wolbachia evolution. Nat Commun. 11:5235. 10.1038/s41467-020-19016-0. 33067437 PMC7568565

[jkaf226-B92] Seemann T . 2014. Prokka: rapid prokaryotic genome annotation. Bioinformatics. 30:2068–2069. 10.1093/bioinformatics/btu153. 24642063

[jkaf226-B93] Shumate A, Salzberg SL. 2021. Liftoff: accurate mapping of gene annotations. Bioinformatics. 37:1639–1643. 10.1093/bioinformatics/btaa1016. 33320174 PMC8289374

[jkaf226-B94] Sinha A et al 2023a. Genomes of the human filarial parasites mansonella perstans and mansonella ozzardi. Front Trop Dis. 4:1139343. 10.3389/fitd.2023.1139343.

[jkaf226-B95] Sinha A et al 2023b. Multiple lineages of nematode-*Wolbachia* symbiosis in supergroup F and convergent loss of bacterioferritin in filarial *Wolbachia*. Genome Biol Evol. 15:evad073. 10.1093/gbe/evad073. 37154102 PMC10195089

[jkaf226-B96] Small ST et al 2019. Human migration and the spread of the nematode parasite wuchereria bancrofti. Mol Biol Evol. 36:1931–1941. 10.1093/molbev/msz116. 31077328 PMC6735882

[jkaf226-B97] Stevens L et al 2024. The genome of litomosoides sigmodontis illuminates the origins of Y chromosomes in filarial nematodes. PLoS Genet. 20:e1011116. 10.1371/journal.pgen.1011116. 38227589 PMC10817185

[jkaf226-B98] Sullivan W . 2017. *Wolbachia*, bottled water, and the dark side of symbiosis. Mol Biol Cell. 28:2343–2346. 10.1091/mbc.E17-02-0132. 28855327 PMC5576898

[jkaf226-B99] Taylor MJ et al 2005. Macrofilaricidal activity after doxycycline treatment of wuchereria bancrofti: a double-blind, randomised placebo-controlled trial. Lancet. 365:2116–2121. 10.1016/S0140-6736(05)66591-9. 15964448

[jkaf226-B100] Teixeira L, Ferreira A, Ashburner M. 2008. The bacterial symbiont Wolbachia induces resistance to RNA viral infections in Drosophila melanogaster. PLoS Biol. 6:e1000002. 10.1371/journal.pbio.1000002. 19222304 PMC2605931

[jkaf226-B101] Tracey A et al 2020. Nearly complete genome sequence of brugia malayi strain FR3. Microbiol Resour Announc. 9:e00154-20. 10.1128/MRA.00154-20. 32527783 PMC7291094

[jkaf226-B102] Tvedte ES et al 2022. Accumulation of endosymbiont genomes in an insect autosome followed by endosymbiont replacement. Curr Biol. 32:2786–2795.e5. 10.1016/j.cub.2022.05.024. 35671755 PMC9311232

[jkaf226-B103] Vancaester E, Blaxter M. 2023. Phylogenomic analysis of Wolbachia genomes from the darwin tree of life biodiversity genomics project. PLoS Biol. 21:e3001972. 10.1371/journal.pbio.3001972. 36689552 PMC9894559

[jkaf226-B104] Wasala SK et al 2019. Variable abundance and distribution of Wolbachia and Cardinium endosymbionts in plant-parasitic nematode field populations. Front Microbiol. 10:964. 10.3389/fmicb.2019.00964. 31134014 PMC6513877

[jkaf226-B105] Werren JH . 1997. Biology of *Wolbachia*. Annu Rev Entomol. 42:587–609. 10.1146/annurev.ento.42.1.587. 15012323

[jkaf226-B106] Wood DE, Lu J, Langmead B. 2019. Improved metagenomic analysis with kraken 2. Genome Biol. 20:257. 10.1186/s13059-019-1891-0. 31779668 PMC6883579

[jkaf226-B107] Wu B et al 2013. Interdomain lateral gene transfer of an essential ferrochelatase gene in human parasitic nematodes. Proc Natl Acad Sci U S A. 110:7748–7753. 10.1073/pnas.1304049110. 23610429 PMC3651471

[jkaf226-B108] Yilmaz E et al 2016. The mitochondrial genomes of the zoonotic canine filarial parasites dirofilaria (nochtiella) repens and Candidatus dirofilaria (nochtiella) Honkongensis provide evidence for presence of cryptic Species. PLoS Negl Trop Dis. 10:e0005028. 10.1371/journal.pntd.0005028. 27727270 PMC5058507

