# Supplementary material for: Ghosts of symbionts past: the hidden history of the dynamic association between filarial nematodes and their Wolbachia endosymbionts
Source: G3 (Bethesda). 2025 Oct 1;15(12):jkaf226. doi: 10.1093/g3journal/jkaf226 (PMC12693587; doi:10.1093/g3journal/jkaf226)
Supplement: jkaf226_Supplementary_Data [file jkaf226_supplementary_data.zip › Supplemental_Material_G3-2025-405948.pdf]

Supplementary Text, Tables and Figures for

Ghosts of symbionts past: The hidden history of the dynamic association between filarial nematodes and their *Wolbachia* endosymbionts

Emmelien Vancaester[1§], Guy R. Oldrieve[2], Alex Reid[2], Georgios Koutsovoulos[2§], Dominik R. Laetsch[2§], Benjamin L. Makepeace[3], Vincent Tanya[4], Sven Poppert[5], Jürgen Krücken[6,7], Adrian Wolstenholme[8], Mark Blaxter[1,2*]

1 Tree of Life, Wellcome Sanger Institute, Hinxton, CB10 1SA, UK

2 School of Biological Sciences, The University of Edinburgh, Edinburgh, Scotland EH9 3TF

3 Institute of Infection, Veterinary & Ecological Sciences, The University of Liverpool, Liverpool L3 5RF, UK

4 Institut de Recherche Agricole pour le Développement, Regional Centre of Wakwa, Ngaoundéré, BP65 Adamawa Region, Cameroon

5 Bernhard Nocht Institute, Bernhard-Nocht-Straße 74, 20359 Hamburg , Germany.

6 Institute for Parasitology and Tropical Veterinary Medicine, Freie Universität Berlin, Berlin, Germany

7 Veterinary Centre for Resistance Research, Freie Universität Berlin, Berlin, Germany

8 Dept of Infectious Diseases, 501 D W Brooks Drive, University of Georgia, Athens, GA 30602 USA

[*] Corresponding author: mark.blaxter@sanger.ac.uk

§ current addresses:

Emmelien Vancaester:

Georgios Koutsovoulos: Institute of Computer Science (ICS), Foundation for Research and Technology - Hellas (FORTH), Nikolaou Plastira 100, Vassilika Vouton, GR-70013 Heraklion, Crete, Greece

Dominik R. Laetsch: FRAMIDOSUR S.L., Av. TT.OO. Neckermann 24, 35100 Maspalomas, Gran Canaria, Spain.

Keywords

filarial nematode, Wolbachia, horizontal gene transfer, symbiosis, genome, hidden Markov model

Author information

Name	*ORCID*	*email*
Emmelien Vancaester	0000-0002-9177-8808	ev3@sanger.ac.uk
Guy R. Oldrieve	0000-0003-1428-0608	guy.oldrieve@ed.ac.uk
Alex Reid		alexandra.reid@icloud.com
Georgios Koutsovoulos	0000-0003-3406-3715	gdkoutsovoulos@gmail.com
Dominik R. Laetsch	0000-0001-7887-0186	dominik.laetsch@gmail.com
Benjamin L. Makepeace	0000-0002-6100-6727	blm1@liverpool.ac.uk
Vincent Tanya	0000-0002-0409-0395	vntanya@yahoo.com
Sven Poppert	0000-0003-4873-249X	sven@poppert.eu
Jürgen Krücken	0000-0002-2842-8100	Juergen.Kruecken@fu-berlin.de
Adrian Wolstenholme	0000-0001-7989-3929	adrianw1906@gmail.com
Mark Blaxter	0000-0003-2861-949X	mark.blaxter@sanger.ac.uk

Author contributions

Name	Conceptualization	Data curation	Formal analysis	Funding acquisition	Investigation	Methodology	Project administration	Software	Resources	Supervision	Validation	Visualization	Writing – original draft	Writing – review & editing
Emmelien Vancaester	X	X	X		X	X		X			X	X	X	X
Guy R. Oldrieve			X		X									X
Alex Reid			X		X									X
Georgios Koutsovoulos			X											X
Dominik R. Laetsch			X					X						X
Benjamin L Makepeace									X					X
Vincent Tanya									X					X
Sven Poppert									X					X
Jürgen Krücken									X					X
Adrian Wolstenholme									X					X
Mark Blaxter	X	X		X	X	X	X			X	X	X	X	X

Supplementary Materials

Supplementary Materials **4**
Supplementary Text 5
Summary metrics of NUWTs found in the genomes of 21 filarial nematodes 5
Supplementary Tables [List] 6
Supplementary Figures [Legends] 7
Supplementary Tables 9
Table S1: Nematode genome data metrics 9
Table S2: Wolbachia genome data 10
Table S3: Dereplicated Wolbachia genomes 35
Table S4: Contigs removed from filarial nematode genomes as likely contaminants. 38
Table S5: Numbers and classification of NUWTs detected in each filarial nematode species genome 39
Table S6: Span of NUWTs detected in each filarial nematode species' genome. 40
Table S7: Average length of NUWTs 41
Table S8: NUWTs from different Wolbachia supergroups 42
Supplementary Figures 43
Figure S1: BlobTools plot of initial Dirofilaria repens assembly 43
Figure S2: Genome phylogeny of Wolbachia 47
Figure S3: Wolbachia proteome clustering 48
Figure S4: Phylogenetic tree of orthologous family OG0000236 and NUWTs 49
Figure S5: Phylogenetic tree of orthologous family OG0000277 and NUWTS 50
Figure S6: Phylogenetic tree of orthologous family OG0000301 and NUWTs 51
Figure S7: Phylogenetic tree of orthologous family OG0000453 and NUWTs 52
Figure S8: Phylogenetic tree of orthologous family OG0000206 and NUWTs 53

Supplementary Text

Summary metrics of NUWTs found in the genomes of 21 filarial nematodes

We screened the genomes of 22 nematode species for NUWTs and identified 21 species in which the NUWTs appeared to be insertions of *Wolbachia*-derived material (see Methods). Individual species had between 17 and 818 insertions (Supplementary Table S5), spanning between 12kb and 343 kb (Supplementary Table S6). The average size of the NUWT insertions was quite similar between nematode species, ranging from 300 to 600 bases. It was notable that the average span of supergroup F NUWTs in *Ce. johnstoni* and *Cr. tuberocauda* was over 1 kb (Supplementary Table S7). The majority of NUWTs came from supergroup C and supergroup D *Wolbachia* donors (Supplementary Table S8)

Supplementary Tables [List]

Supplementary Tables are available in the Excel spreadsheet in the Supplementary Data online.

Table S1: Nematode genome data metrics

Table S2: *Wolbachia* genome data

Table S3: Dereplicated *Wolbachia* genomes

Table S4: Contigs removed from filarial nematode genomes as likely contaminants.

Table S5: Numbers and classification of NUWTs detected in each filarial nematode species genome

Table S6: Span of NUWTs detected in each filarial nematode species' genome.

Table S7: Average length of NUWTs.

Table S8: NUWTs from different *Wolbachia* supergroups.

Supplementary Figures [Legends]

(All supplementary Figures are in one PDF)

Figure S1: BlobTools plot of initial *Dirofilaria repens* assembly

A BlobTools (v1) plot of the *Dr. repens* assembly showing (red) contigs assigned to Nematoda (i.e. the nematode host) and (green) contigs assigned to Proteobacteria (i.e. *Wolbachia*). The Protobacteria contigs (coloured green) form two clusters, one at ~300 fold coverage and 35% GC, and one at ~200 fold coverage and 28% GC. These correspond to the C supergroup (high coverage) and F supergroup (low coverage) *Wolbachia* coinfecting this sample.

Figure S2: Genome phylogeny of *Wolbachia*

A. Summary phylogeny of 1,444 *Wolbachia* genomes. The phylogeny was rooted with genomes from seven *Anaplasma*, *Ehrlichia* and *Mesenet* species. Supergroups are indicated with bold letters.

B. Unrooted phylogeny of 167 selected *Wolbachia* genomes (as in Figure 1B) with sources named. Supergroups are indicated with bold letters.

C. Rooted phylogeny of 167 selected *Wolbachia* genomes (as in Figure 1B) with sources named.

Figure S3: *Wolbachia* proteome clustering

A. Rarefaction curves describing the proteome diversity found across *Wolbachia* and within supergroup A and B. *Wolbachia* proteomes selected with dRep and deemed near-complete were added one by one and the number of clusters with >1 member tallied. The standard deviations are derived from 50,000 repetitions of the clustering with randomised addition order of genomes. The upper curves are for orthogroups with >1 member, while the lower curves indicate the number of singleton sequences.

B. Rarefaction curves describing the proteome diversity found within supergroup C, D and F. *Wolbachia* proteomes selected with dRep and deemed near-complete were added one by one and the number of clusters with >1 member tallied. The standard deviations are derived from 50,000 repetitions of the clustering with randomised addition order of genomes. The upper curves are for orthogroups with >1 member, while the lower curves indicate the number of singleton sequences.

Figure S4: Phylogenetic tree of orthologous family OG0000236 and NUWTs

Phylogenetic tree of dihydrolipoyl dehydrogenase (OG0000236), illustrating the phylogenetic placement of NUWTs deriving from both C and F *Wolbachia* in *Dirofilaria immitis* (highlighted in bold).

Sequences derived from living *Wolbachia* are indicated by the supergroup and host species nomenclature followed by the locus number (e.g. "E_wNcom_00841") or supergroup and GCA nomenclature followed by the locus number (e.g. "I_GCA012277295_00789"), while NUWTS are indicated by the nematode species abbreviation and their location (e.g. "Dimm_JAKNDB010000109.1_OG0000236_4295875-4296316" is from *D. immitis*, contig

JAKNDB010000109.1; it matches OG0000236 and is from bases 4295875-4296316 in the contig).

Figure S6: Phylogenetic tree of orthologous family OG0000301 and NUWTs

Phylogenetic tree of DNA-directed RNA polymerase subunit alpha (OG0000301) showing the phylogenetic placement of NUWTs from four *Onchocerca* species. Nomenclature as in Figure S4.

Figure S5: Phylogenetic tree of orthologous family OG0000277 and NUWTS

Phylogenetic tree of peptide deformylase (OG0000277) showing the phylogenetic placement of NUWTs from *Madathamugadia hiepeia*. The NUWTs likely derive from C, D and J *Wolbachia*. Nomenclature as in Figure S4.

Figure S7: Phylogenetic tree of orthologous family OG0000453 and NUWTs

Phylogenetic tree of signal peptidase I (OG0000453) showing the phylogenetic placement of NUWTs from three *Brugia* species. Nomenclature as in Figure S4.

Figure S8: Phylogenetic tree of orthologous family OG0000206 and NUWTs

Phylogenetic tree of ATP-dependent zinc metalloprotease FtsH (OG0000206), showing the phylogenetic placement of NUWTs from two *Brugia* species. Nomenclature as in Figure S4.

Supplementary Tables

Table S1: Nematode genome data metrics

Species	Sequencing centre	Assembly version	BUSCO (genome, nematoda_odb10)		span	contig N50
			complete	duplicated		
Acanthocheilonema viteae	University of Edinburgh	GCA_900537255.1	88.8	1.7	77350906	25808
Brugia malayi	WormBase Parasite/EBI	GCF_000002995.4	98.2	0.5	87155713	14214749
Brugia pahangi	University of Maryland	GCA_012070555.1	97.5	2.4	96392917	10892846
Brugia timori	Wellcome Sanger Institute	GCA_900618025.1	56.8	0.5	64930714	2306
Cercopithifilaria (Ce.) johnstoni	Wellcome Sanger Institute	GCA_916381525.1	95.5	0.2	76938708	99003
Cruorifilaria (Cr.) tuberocauda	New England Biolabs	GCA_013365365.1	96.1	0.4	75516636	105487
Dipetalonema (Dp.) caudispina	New England Biolabs	GCA_013365325.1	97.8	0.2	81585392	132317
Dirofilaria (Dr.) immitis	CIBIO-InBIO	GCA_024305405.1	94.1	0.4	86813779	4253153
Dirofilaria (Dr.) repens	ETH/UZH	GCA_008729115.1	94.3	3.9	99578628	584065
Elaeophora elaphii	Wellcome Sanger Institute	GCA_000499685.1	87.2	0.3	82568297	99433
Litomosoides (Li.) brasiliensis	New England Biolabs	GCA_013365375.1	95.1	0.2	65202511	147890
Litomosoides (Li.) sigmodontis	Wellcome Sanger Institute	GCA_963070105.1	95.5	0.3	65877608	10903439
Loa (Lo.) loa	Broad Institute	GCF_000183805.2	97.4	0.1	91365832	174388
Madathamugadia (Md.) hiepei	New England Biolabs	GCA_013365335.1	88.3	10.1	77701753	17407
Mansonella (Ma.) ozzardi	New England Biolabs	GCA_029876185.1	93.8	0.8	76054666	285814
Mansonella (Ma.) perstans	Institute of Tropical Medicine, Tuebingen, Germany	GCA_947561605.2	94.7	0.4	79599925	173569
Onchocerca flexuosa	McDonnell Genome Institute	GCA_002249935.1	71.8	0.6	67740367	540294
Onchocerca lupi	Northern Arizona University	GCA_028564675.1	95.6	0.6	92491485	96493
Onchocerca ochengi	Wellcome Sanger Institute	GCA_000950515.2	85.1	0.6	91660559	16199
Onchocerca volvulus	Wellcome Sanger Institute	GCA_000499405.2	97.9	0.6	96340582	25485961
Setaria labiatopapillosa	University of Edinburgh	[to be advised]	97.1	0.3	82384673	129940
Wuchereria bancrofti	Case Western Reserve University	GCA_005281725.1	97.9	1.0	88416250	12368652

Table S2: *Wolbachia* genome data

Assembly name (INSDC GCA accession or given name)	Supergroup	Host group	Host
GCA_947250015	A	Arthropod	*Acrocera orbiculus*
GCA_026768255	A	Arthropod	*Acromyrmex echinatior*
GCA_017896245	A	Arthropod	*Aedes aegypti*
GCA_017896265	A	Arthropod	*Aedes aegypti*
GCA_017896285	A	Arthropod	*Aedes aegypti*
GCA_017896305	A	Arthropod	*Aedes aegypti*
GCA_017896325	A	Arthropod	*Aedes aegypti*
GCA_017896345	A	Arthropod	*Aedes aegypti*
GCA_017896365	A	Arthropod	*Aedes aegypti*
GCA_021496155	A	Arthropod	*Aedes aegypti*
GCA_021496175	A	Arthropod	*Aedes aegypti*
GCA_021496195	A	Arthropod	*Aedes aegypti*
GCA_021496215	A	Arthropod	*Aedes aegypti*
GCA_021496235	A	Arthropod	*Aedes aegypti*
GCA_021497205	A	Arthropod	*Aedes aegypti*
GCA_021497225	A	Arthropod	*Aedes aegypti*
GCA_021497245	A	Arthropod	*Aedes aegypti*
GCA_022343845	A	Arthropod	*Aedes aegypti*
GCA_022343885	A	Arthropod	*Aedes aegypti*
GCA_022343905	A	Arthropod	*Aedes aegypti*
GCA_022343925	A	Arthropod	*Aedes aegypti*
GCA_024804185	A	Arthropod	*Aedes albopictus*
GCA_002379175	A	Arthropod	*Aedes albopictus*
GCA_002379155	A	Arthropod	*Aedes albopictus*
GCA_947250785	A	Arthropod	*Ancistrocerus nigricornis*
GCA_947251685	A	Arthropod	*Andrena dorsata*
GCA_947251765	A	Arthropod	*Andrena dorsata*
GCA_947250565	A	Arthropod	*Andrena haemorrhoa*
GCA_947179565	A	Arthropod	*Andrena hattorfiana*
GCA_947251545	A	Arthropod	*Anomoia purmunda*
GCA_947251795	A	Arthropod	*Anoplius nigerrimus*
GCA_014333535	A	Arthropod	*Anoplolepis gracilipes*
GCA_947251615	A	Arthropod	*Apoderus coryli*
GCA_902646855	A	Arthropod	*Apoidea*
GCA_902648465	A	Arthropod	*Apoidea sp.*
GCA_010820705	A	Arthropod	*Apterostigma dentigerum*
GCA_947251645	A	Arthropod	*Bibio marci*
GCA_947251895	A	Arthropod	*Bombylius major*
GCA_947251525	A	Arthropod	*Calamotropha paludella*
GCA_947251935	A	Arthropod	*Calamotropha paludella*
GCA_902646905	A	Arthropod	*Camponotus obliquus*
GCA_023052945	A	Arthropod	*Camponotus pennsylvanicus*
GCA_902713635	A	Arthropod	*Cardiocondyla obscurior*
GCA_902713645	A	Arthropod	*Cardiocondyla obscurior*
GCA_006542295	A	Arthropod	*Carposina sasakii*
GCA_018454455	A	Arthropod	*Ceratitis capitata*
GCA_017869155	A	Arthropod	*Ceratosolen solmsi*
GCA_947179435	A	Arthropod	*Cheilosia soror*
GCA_947250625	A	Arthropod	*Cheilosia soror*
GCA_947251955	A	Arthropod	*Coremacera marginata*
GCA_947179475	A	Arthropod	*Cydia splendana*
GCA_001648025	A	Arthropod	*Dactylopius coccus*
GCA_021609905	A	Arthropod	*Delia radicum*
GCA_902636385	A	Arthropod	*Diabrotica virgifera*
GCA_902636345	A	Arthropod	*Diabrotica virgifera virgifera*

GCA_902636365	A	Arthropod	*Diabrotica virgifera virgifera*
GCA_902636425	A	Arthropod	*Diabrotica virgifera virgifera*
GCA_902636475	A	Arthropod	*Diabrotica virgifera virgifera*
GCA_902636485	A	Arthropod	*Diabrotica virgifera virgifera*
GCA_902636495	A	Arthropod	*Diabrotica virgifera virgifera*
GCA_902636515	A	Arthropod	*Diabrotica virgifera virgifera*
GCA_902636525	A	Arthropod	*Diabrotica virgifera virgifera*
GCA_902636535	A	Arthropod	*Diabrotica virgifera virgifera*
GCA_902636545	A	Arthropod	*Diabrotica virgifera virgifera*
GCA_902636565	A	Arthropod	*Diabrotica virgifera virgifera*
GCA_902636585	A	Arthropod	*Diabrotica virgifera virgifera*
GCA_902636595	A	Arthropod	*Diabrotica virgifera virgifera*
GCA_902636605	A	Arthropod	*Diabrotica virgifera virgifera*
GCA_902636615	A	Arthropod	*Diabrotica virgifera virgifera*
GCA_902636635	A	Arthropod	*Diabrotica virgifera virgifera*
GCA_902636645	A	Arthropod	*Diabrotica virgifera virgifera*
GCA_902636655	A	Arthropod	*Diabrotica virgifera virgifera*
GCA_902636665	A	Arthropod	*Diabrotica virgifera virgifera*
GCA_902636675	A	Arthropod	*Diabrotica virgifera virgifera*
GCA_902636685	A	Arthropod	*Diabrotica virgifera virgifera*
GCA_902646925	A	Arthropod	*Diachasma alloeum*
GCA_902646845	A	Arthropod	*Diachasma alloeum*
GCA_902646875	A	Arthropod	*Diachasma alloeum*
GCA_902646885	A	Arthropod	*Diachasma alloeum*
GCA_902648475	A	Arthropod	*Diachasma alloeum*
GCA_008033215	A	Arthropod	*Drosophila ananassae*
GCA_000167475	A	Arthropod	*Drosophila ananassae*
GCA_002907405	A	Arthropod	*Drosophila ananassae*
GCA_002907425	A	Arthropod	*Drosophila ananassae*
GCA_003671365	A	Arthropod	*Drosophila ananassae*
GCA_003671375	A	Arthropod	*Drosophila ananassae*
GCA_003671405	A	Arthropod	*Drosophila ananassae*
GCA_902636775	A	Arthropod	*Drosophila ananassae*
GCA_902636875	A	Arthropod	*Drosophila ananassae*
GCA_902636885	A	Arthropod	*Drosophila ananassae*
GCA_902636895	A	Arthropod	*Drosophila ananassae*
GCA_902636905	A	Arthropod	*Drosophila ananassae*
GCA_902636915	A	Arthropod	*Drosophila ananassae*
GCA_902636925	A	Arthropod	*Drosophila ananassae*
GCA_902636935	A	Arthropod	*Drosophila ananassae*
GCA_902636945	A	Arthropod	*Drosophila ananassae*
GCA_902636955	A	Arthropod	*Drosophila ananassae*
GCA_902636965	A	Arthropod	*Drosophila ananassae*
GCA_902646065	A	Arthropod	*Drosophila ananassae*
GCA_902646075	A	Arthropod	*Drosophila ananassae*
GCA_902646085	A	Arthropod	*Drosophila ananassae*
GCA_902646105	A	Arthropod	*Drosophila ananassae*
GCA_902646135	A	Arthropod	*Drosophila ananassae*
GCA_902646165	A	Arthropod	*Drosophila ananassae*
GCA_902646175	A	Arthropod	*Drosophila ananassae*
GCA_902646195	A	Arthropod	*Drosophila ananassae*
GCA_902646215	A	Arthropod	*Drosophila ananassae*
GCA_902646235	A	Arthropod	*Drosophila ananassae*
GCA_014129655	A	Arthropod	*Drosophila arawakana*
GCA_017916175	A	Arthropod	*Drosophila auraria*
GCA_028982185	A	Arthropod	*Drosophila baimaii*
GCA_014129605	A	Arthropod	*Drosophila baimaii*
GCA_028982105	A	Arthropod	*Drosophila bicornuta*
GCA_014129645	A	Arthropod	*Drosophila bicornuta*

GCA_014129685	A	Arthropod	*Drosophila bifasciata*
GCA_028982005	A	Arthropod	*Drosophila bocki*
GCA_014129615	A	Arthropod	*Drosophila borealis*
GCA_028981925	A	Arthropod	*Drosophila burlai*
GCA_028982165	A	Arthropod	*Drosophila chauvacae*
GCA_001758565	A	Arthropod	*Drosophila incompta*
GCA_001758585	A	Arthropod	*Drosophila incompta*
GCA_021378375	A	Arthropod	*Drosophila innubila*
GCA_028981785	A	Arthropod	*Drosophila leontia*
GCA_902643455	A	Arthropod	*Drosophila melanogaster*
GCA_902643495	A	Arthropod	*Drosophila melanogaster*
GCA_000008025	A	Arthropod	*Drosophila melanogaster*
GCA_000475015	A	Arthropod	*Drosophila melanogaster*
GCA_002907445	A	Arthropod	*Drosophila melanogaster*
GCA_002907525	A	Arthropod	*Drosophila melanogaster*
GCA_007971685	A	Arthropod	*Drosophila melanogaster*
GCA_007972595	A	Arthropod	*Drosophila melanogaster*
GCA_007972745	A	Arthropod	*Drosophila melanogaster*
GCA_014354335	A	Arthropod	*Drosophila melanogaster*
GCA_014354345	A	Arthropod	*Drosophila melanogaster*
GCA_016584325	A	Arthropod	*Drosophila melanogaster*
GCA_016584355	A	Arthropod	*Drosophila melanogaster*
GCA_016584375	A	Arthropod	*Drosophila melanogaster*
GCA_016584405	A	Arthropod	*Drosophila melanogaster*
GCA_016584425	A	Arthropod	*Drosophila melanogaster*
GCA_017916155	A	Arthropod	*Drosophila melanogaster*
GCA_021347805	A	Arthropod	*Drosophila melanogaster*
GCA_021347845	A	Arthropod	*Drosophila melanogaster*
GCA_022343865	A	Arthropod	*Drosophila melanogaster*
GCA_902636975	A	Arthropod	*Drosophila melanogaster*
GCA_902636985	A	Arthropod	*Drosophila melanogaster*
GCA_902636995	A	Arthropod	*Drosophila melanogaster*
GCA_902637005	A	Arthropod	*Drosophila melanogaster*
GCA_902637015	A	Arthropod	*Drosophila melanogaster*
GCA_902637025	A	Arthropod	*Drosophila melanogaster*
GCA_902637035	A	Arthropod	*Drosophila melanogaster*
GCA_902637045	A	Arthropod	*Drosophila melanogaster*
GCA_902637055	A	Arthropod	*Drosophila melanogaster*
GCA_902637065	A	Arthropod	*Drosophila melanogaster*
GCA_902637075	A	Arthropod	*Drosophila melanogaster*
GCA_902637085	A	Arthropod	*Drosophila melanogaster*
GCA_902637095	A	Arthropod	*Drosophila melanogaster*
GCA_902637105	A	Arthropod	*Drosophila melanogaster*
GCA_902637115	A	Arthropod	*Drosophila melanogaster*
GCA_902637125	A	Arthropod	*Drosophila melanogaster*
GCA_902637135	A	Arthropod	*Drosophila melanogaster*
GCA_902637145	A	Arthropod	*Drosophila melanogaster*
GCA_902637155	A	Arthropod	*Drosophila melanogaster*
GCA_902637165	A	Arthropod	*Drosophila melanogaster*
GCA_902637175	A	Arthropod	*Drosophila melanogaster*
GCA_902637185	A	Arthropod	*Drosophila melanogaster*
GCA_902637195	A	Arthropod	*Drosophila melanogaster*
GCA_902637205	A	Arthropod	*Drosophila melanogaster*
GCA_902637215	A	Arthropod	*Drosophila melanogaster*
GCA_902637225	A	Arthropod	*Drosophila melanogaster*
GCA_902637235	A	Arthropod	*Drosophila melanogaster*
GCA_902637245	A	Arthropod	*Drosophila melanogaster*
GCA_902637255	A	Arthropod	*Drosophila melanogaster*
GCA_902637265	A	Arthropod	*Drosophila melanogaster*

GCA_902637275	A	Arthropod	*Drosophila melanogaster*
GCA_902637285	A	Arthropod	*Drosophila melanogaster*
GCA_902637295	A	Arthropod	*Drosophila melanogaster*
GCA_902637305	A	Arthropod	*Drosophila melanogaster*
GCA_902637315	A	Arthropod	*Drosophila melanogaster*
GCA_902637325	A	Arthropod	*Drosophila melanogaster*
GCA_902637335	A	Arthropod	*Drosophila melanogaster*
GCA_902637345	A	Arthropod	*Drosophila melanogaster*
GCA_902637355	A	Arthropod	*Drosophila melanogaster*
GCA_902637365	A	Arthropod	*Drosophila melanogaster*
GCA_902637375	A	Arthropod	*Drosophila melanogaster*
GCA_902637385	A	Arthropod	*Drosophila melanogaster*
GCA_902637395	A	Arthropod	*Drosophila melanogaster*
GCA_902637405	A	Arthropod	*Drosophila melanogaster*
GCA_902637415	A	Arthropod	*Drosophila melanogaster*
GCA_902637425	A	Arthropod	*Drosophila melanogaster*
GCA_902637435	A	Arthropod	*Drosophila melanogaster*
GCA_902637445	A	Arthropod	*Drosophila melanogaster*
GCA_902637455	A	Arthropod	*Drosophila melanogaster*
GCA_902637465	A	Arthropod	*Drosophila melanogaster*
GCA_902637475	A	Arthropod	*Drosophila melanogaster*
GCA_902637485	A	Arthropod	*Drosophila melanogaster*
GCA_902637495	A	Arthropod	*Drosophila melanogaster*
GCA_902637505	A	Arthropod	*Drosophila melanogaster*
GCA_902637515	A	Arthropod	*Drosophila melanogaster*
GCA_902637525	A	Arthropod	*Drosophila melanogaster*
GCA_902637535	A	Arthropod	*Drosophila melanogaster*
GCA_902637545	A	Arthropod	*Drosophila melanogaster*
GCA_902637555	A	Arthropod	*Drosophila melanogaster*
GCA_902637565	A	Arthropod	*Drosophila melanogaster*
GCA_902637575	A	Arthropod	*Drosophila melanogaster*
GCA_902637585	A	Arthropod	*Drosophila melanogaster*
GCA_902637595	A	Arthropod	*Drosophila melanogaster*
GCA_902637605	A	Arthropod	*Drosophila melanogaster*
GCA_902637615	A	Arthropod	*Drosophila melanogaster*
GCA_902637625	A	Arthropod	*Drosophila melanogaster*
GCA_902637635	A	Arthropod	*Drosophila melanogaster*
GCA_902637645	A	Arthropod	*Drosophila melanogaster*
GCA_902637655	A	Arthropod	*Drosophila melanogaster*
GCA_902637665	A	Arthropod	*Drosophila melanogaster*
GCA_902637675	A	Arthropod	*Drosophila melanogaster*
GCA_902637685	A	Arthropod	*Drosophila melanogaster*
GCA_902637695	A	Arthropod	*Drosophila melanogaster*
GCA_902637705	A	Arthropod	*Drosophila melanogaster*
GCA_902637715	A	Arthropod	*Drosophila melanogaster*
GCA_902637725	A	Arthropod	*Drosophila melanogaster*
GCA_902637735	A	Arthropod	*Drosophila melanogaster*
GCA_902637745	A	Arthropod	*Drosophila melanogaster*
GCA_902637755	A	Arthropod	*Drosophila melanogaster*
GCA_902637765	A	Arthropod	*Drosophila melanogaster*
GCA_902637775	A	Arthropod	*Drosophila melanogaster*
GCA_902637785	A	Arthropod	*Drosophila melanogaster*
GCA_902637795	A	Arthropod	*Drosophila melanogaster*
GCA_902637805	A	Arthropod	*Drosophila melanogaster*
GCA_902637815	A	Arthropod	*Drosophila melanogaster*
GCA_902637825	A	Arthropod	*Drosophila melanogaster*
GCA_902637835	A	Arthropod	*Drosophila melanogaster*
GCA_902637845	A	Arthropod	*Drosophila melanogaster*
GCA_902637855	A	Arthropod	*Drosophila melanogaster*

GCA_902637865	A	Arthropod	*Drosophila melanogaster*
GCA_902637875	A	Arthropod	*Drosophila melanogaster*
GCA_902637885	A	Arthropod	*Drosophila melanogaster*
GCA_902637895	A	Arthropod	*Drosophila melanogaster*
GCA_902637905	A	Arthropod	*Drosophila melanogaster*
GCA_902637915	A	Arthropod	*Drosophila melanogaster*
GCA_902637925	A	Arthropod	*Drosophila melanogaster*
GCA_902637935	A	Arthropod	*Drosophila melanogaster*
GCA_902637945	A	Arthropod	*Drosophila melanogaster*
GCA_902637955	A	Arthropod	*Drosophila melanogaster*
GCA_902637965	A	Arthropod	*Drosophila melanogaster*
GCA_902637975	A	Arthropod	*Drosophila melanogaster*
GCA_902637985	A	Arthropod	*Drosophila melanogaster*
GCA_902637995	A	Arthropod	*Drosophila melanogaster*
GCA_902638005	A	Arthropod	*Drosophila melanogaster*
GCA_902638015	A	Arthropod	*Drosophila melanogaster*
GCA_902638025	A	Arthropod	*Drosophila melanogaster*
GCA_902638035	A	Arthropod	*Drosophila melanogaster*
GCA_902638045	A	Arthropod	*Drosophila melanogaster*
GCA_902638055	A	Arthropod	*Drosophila melanogaster*
GCA_902638065	A	Arthropod	*Drosophila melanogaster*
GCA_902638075	A	Arthropod	*Drosophila melanogaster*
GCA_902638085	A	Arthropod	*Drosophila melanogaster*
GCA_902638095	A	Arthropod	*Drosophila melanogaster*
GCA_902638105	A	Arthropod	*Drosophila melanogaster*
GCA_902638115	A	Arthropod	*Drosophila melanogaster*
GCA_902638125	A	Arthropod	*Drosophila melanogaster*
GCA_902638135	A	Arthropod	*Drosophila melanogaster*
GCA_902638145	A	Arthropod	*Drosophila melanogaster*
GCA_902638155	A	Arthropod	*Drosophila melanogaster*
GCA_902638165	A	Arthropod	*Drosophila melanogaster*
GCA_902638175	A	Arthropod	*Drosophila melanogaster*
GCA_902638185	A	Arthropod	*Drosophila melanogaster*
GCA_902638195	A	Arthropod	*Drosophila melanogaster*
GCA_902638205	A	Arthropod	*Drosophila melanogaster*
GCA_902638215	A	Arthropod	*Drosophila melanogaster*
GCA_902638225	A	Arthropod	*Drosophila melanogaster*
GCA_902638235	A	Arthropod	*Drosophila melanogaster*
GCA_902638245	A	Arthropod	*Drosophila melanogaster*
GCA_902638255	A	Arthropod	*Drosophila melanogaster*
GCA_902638265	A	Arthropod	*Drosophila melanogaster*
GCA_902638275	A	Arthropod	*Drosophila melanogaster*
GCA_902638285	A	Arthropod	*Drosophila melanogaster*
GCA_902638295	A	Arthropod	*Drosophila melanogaster*
GCA_902638305	A	Arthropod	*Drosophila melanogaster*
GCA_902638315	A	Arthropod	*Drosophila melanogaster*
GCA_902638325	A	Arthropod	*Drosophila melanogaster*
GCA_902638335	A	Arthropod	*Drosophila melanogaster*
GCA_902638345	A	Arthropod	*Drosophila melanogaster*
GCA_902638355	A	Arthropod	*Drosophila melanogaster*
GCA_902638365	A	Arthropod	*Drosophila melanogaster*
GCA_902638375	A	Arthropod	*Drosophila melanogaster*
GCA_902638385	A	Arthropod	*Drosophila melanogaster*
GCA_902638395	A	Arthropod	*Drosophila melanogaster*
GCA_902638405	A	Arthropod	*Drosophila melanogaster*
GCA_902638415	A	Arthropod	*Drosophila melanogaster*
GCA_902638425	A	Arthropod	*Drosophila melanogaster*
GCA_902638435	A	Arthropod	*Drosophila melanogaster*
GCA_902638445	A	Arthropod	*Drosophila melanogaster*

GCA_902638455	A	Arthropod	*Drosophila melanogaster*
GCA_902638465	A	Arthropod	*Drosophila melanogaster*
GCA_902638475	A	Arthropod	*Drosophila melanogaster*
GCA_902638485	A	Arthropod	*Drosophila melanogaster*
GCA_902638495	A	Arthropod	*Drosophila melanogaster*
GCA_902638505	A	Arthropod	*Drosophila melanogaster*
GCA_902638515	A	Arthropod	*Drosophila melanogaster*
GCA_902638525	A	Arthropod	*Drosophila melanogaster*
GCA_902638535	A	Arthropod	*Drosophila melanogaster*
GCA_902638545	A	Arthropod	*Drosophila melanogaster*
GCA_902638555	A	Arthropod	*Drosophila melanogaster*
GCA_902638565	A	Arthropod	*Drosophila melanogaster*
GCA_902638575	A	Arthropod	*Drosophila melanogaster*
GCA_902638585	A	Arthropod	*Drosophila melanogaster*
GCA_902638595	A	Arthropod	*Drosophila melanogaster*
GCA_902638605	A	Arthropod	*Drosophila melanogaster*
GCA_902638615	A	Arthropod	*Drosophila melanogaster*
GCA_902638625	A	Arthropod	*Drosophila melanogaster*
GCA_902638635	A	Arthropod	*Drosophila melanogaster*
GCA_902638645	A	Arthropod	*Drosophila melanogaster*
GCA_902638655	A	Arthropod	*Drosophila melanogaster*
GCA_902638665	A	Arthropod	*Drosophila melanogaster*
GCA_902638675	A	Arthropod	*Drosophila melanogaster*
GCA_902638685	A	Arthropod	*Drosophila melanogaster*
GCA_902638695	A	Arthropod	*Drosophila melanogaster*
GCA_902638705	A	Arthropod	*Drosophila melanogaster*
GCA_902638715	A	Arthropod	*Drosophila melanogaster*
GCA_902638725	A	Arthropod	*Drosophila melanogaster*
GCA_902638735	A	Arthropod	*Drosophila melanogaster*
GCA_902638745	A	Arthropod	*Drosophila melanogaster*
GCA_902638755	A	Arthropod	*Drosophila melanogaster*
GCA_902638765	A	Arthropod	*Drosophila melanogaster*
GCA_902638775	A	Arthropod	*Drosophila melanogaster*
GCA_902638785	A	Arthropod	*Drosophila melanogaster*
GCA_902638795	A	Arthropod	*Drosophila melanogaster*
GCA_902638805	A	Arthropod	*Drosophila melanogaster*
GCA_902638815	A	Arthropod	*Drosophila melanogaster*
GCA_902638825	A	Arthropod	*Drosophila melanogaster*
GCA_902638835	A	Arthropod	*Drosophila melanogaster*
GCA_902638845	A	Arthropod	*Drosophila melanogaster*
GCA_902638855	A	Arthropod	*Drosophila melanogaster*
GCA_902638865	A	Arthropod	*Drosophila melanogaster*
GCA_902638875	A	Arthropod	*Drosophila melanogaster*
GCA_902638885	A	Arthropod	*Drosophila melanogaster*
GCA_902638895	A	Arthropod	*Drosophila melanogaster*
GCA_902638905	A	Arthropod	*Drosophila melanogaster*
GCA_902638915	A	Arthropod	*Drosophila melanogaster*
GCA_902638925	A	Arthropod	*Drosophila melanogaster*
GCA_902638935	A	Arthropod	*Drosophila melanogaster*
GCA_902638945	A	Arthropod	*Drosophila melanogaster*
GCA_902638955	A	Arthropod	*Drosophila melanogaster*
GCA_902638965	A	Arthropod	*Drosophila melanogaster*
GCA_902638975	A	Arthropod	*Drosophila melanogaster*
GCA_902638985	A	Arthropod	*Drosophila melanogaster*
GCA_902638995	A	Arthropod	*Drosophila melanogaster*
GCA_902639005	A	Arthropod	*Drosophila melanogaster*
GCA_902639015	A	Arthropod	*Drosophila melanogaster*
GCA_902639025	A	Arthropod	*Drosophila melanogaster*
GCA_902639035	A	Arthropod	*Drosophila melanogaster*

GCA_902639045	A	Arthropod	*Drosophila melanogaster*
GCA_902639055	A	Arthropod	*Drosophila melanogaster*
GCA_902639065	A	Arthropod	*Drosophila melanogaster*
GCA_902639075	A	Arthropod	*Drosophila melanogaster*
GCA_902639085	A	Arthropod	*Drosophila melanogaster*
GCA_902639095	A	Arthropod	*Drosophila melanogaster*
GCA_902639105	A	Arthropod	*Drosophila melanogaster*
GCA_902639115	A	Arthropod	*Drosophila melanogaster*
GCA_902639145	A	Arthropod	*Drosophila melanogaster*
GCA_902639155	A	Arthropod	*Drosophila melanogaster*
GCA_902639165	A	Arthropod	*Drosophila melanogaster*
GCA_902639175	A	Arthropod	*Drosophila melanogaster*
GCA_902639185	A	Arthropod	*Drosophila melanogaster*
GCA_902639195	A	Arthropod	*Drosophila melanogaster*
GCA_902639205	A	Arthropod	*Drosophila melanogaster*
GCA_902639215	A	Arthropod	*Drosophila melanogaster*
GCA_902639225	A	Arthropod	*Drosophila melanogaster*
GCA_902639235	A	Arthropod	*Drosophila melanogaster*
GCA_902639245	A	Arthropod	*Drosophila melanogaster*
GCA_902639255	A	Arthropod	*Drosophila melanogaster*
GCA_902639265	A	Arthropod	*Drosophila melanogaster*
GCA_902639275	A	Arthropod	*Drosophila melanogaster*
GCA_902639285	A	Arthropod	*Drosophila melanogaster*
GCA_902639295	A	Arthropod	*Drosophila melanogaster*
GCA_902639305	A	Arthropod	*Drosophila melanogaster*
GCA_902639315	A	Arthropod	*Drosophila melanogaster*
GCA_902639325	A	Arthropod	*Drosophila melanogaster*
GCA_902639335	A	Arthropod	*Drosophila melanogaster*
GCA_902639345	A	Arthropod	*Drosophila melanogaster*
GCA_902639355	A	Arthropod	*Drosophila melanogaster*
GCA_902639365	A	Arthropod	*Drosophila melanogaster*
GCA_902639375	A	Arthropod	*Drosophila melanogaster*
GCA_902639385	A	Arthropod	*Drosophila melanogaster*
GCA_902639395	A	Arthropod	*Drosophila melanogaster*
GCA_902639405	A	Arthropod	*Drosophila melanogaster*
GCA_902639415	A	Arthropod	*Drosophila melanogaster*
GCA_902639425	A	Arthropod	*Drosophila melanogaster*
GCA_902639435	A	Arthropod	*Drosophila melanogaster*
GCA_902639445	A	Arthropod	*Drosophila melanogaster*
GCA_902639455	A	Arthropod	*Drosophila melanogaster*
GCA_902639465	A	Arthropod	*Drosophila melanogaster*
GCA_902639475	A	Arthropod	*Drosophila melanogaster*
GCA_902639485	A	Arthropod	*Drosophila melanogaster*
GCA_902639495	A	Arthropod	*Drosophila melanogaster*
GCA_902639505	A	Arthropod	*Drosophila melanogaster*
GCA_902639515	A	Arthropod	*Drosophila melanogaster*
GCA_902639525	A	Arthropod	*Drosophila melanogaster*
GCA_902639535	A	Arthropod	*Drosophila melanogaster*
GCA_902639545	A	Arthropod	*Drosophila melanogaster*
GCA_902639555	A	Arthropod	*Drosophila melanogaster*
GCA_902639565	A	Arthropod	*Drosophila melanogaster*
GCA_902639575	A	Arthropod	*Drosophila melanogaster*
GCA_902639585	A	Arthropod	*Drosophila melanogaster*
GCA_902639595	A	Arthropod	*Drosophila melanogaster*
GCA_902639605	A	Arthropod	*Drosophila melanogaster*
GCA_902639615	A	Arthropod	*Drosophila melanogaster*
GCA_902639625	A	Arthropod	*Drosophila melanogaster*
GCA_902639635	A	Arthropod	*Drosophila melanogaster*
GCA_902639645	A	Arthropod	*Drosophila melanogaster*

GCA_902639655	A	Arthropod	*Drosophila melanogaster*
GCA_902639665	A	Arthropod	*Drosophila melanogaster*
GCA_902639675	A	Arthropod	*Drosophila melanogaster*
GCA_902639685	A	Arthropod	*Drosophila melanogaster*
GCA_902639695	A	Arthropod	*Drosophila melanogaster*
GCA_902639705	A	Arthropod	*Drosophila melanogaster*
GCA_902639715	A	Arthropod	*Drosophila melanogaster*
GCA_902639725	A	Arthropod	*Drosophila melanogaster*
GCA_902639735	A	Arthropod	*Drosophila melanogaster*
GCA_902639745	A	Arthropod	*Drosophila melanogaster*
GCA_902639755	A	Arthropod	*Drosophila melanogaster*
GCA_902639765	A	Arthropod	*Drosophila melanogaster*
GCA_902639775	A	Arthropod	*Drosophila melanogaster*
GCA_902639785	A	Arthropod	*Drosophila melanogaster*
GCA_902639795	A	Arthropod	*Drosophila melanogaster*
GCA_902639805	A	Arthropod	*Drosophila melanogaster*
GCA_902639815	A	Arthropod	*Drosophila melanogaster*
GCA_902639825	A	Arthropod	*Drosophila melanogaster*
GCA_902639835	A	Arthropod	*Drosophila melanogaster*
GCA_902639845	A	Arthropod	*Drosophila melanogaster*
GCA_902639855	A	Arthropod	*Drosophila melanogaster*
GCA_902639865	A	Arthropod	*Drosophila melanogaster*
GCA_902639875	A	Arthropod	*Drosophila melanogaster*
GCA_902639885	A	Arthropod	*Drosophila melanogaster*
GCA_902639895	A	Arthropod	*Drosophila melanogaster*
GCA_902639905	A	Arthropod	*Drosophila melanogaster*
GCA_902639915	A	Arthropod	*Drosophila melanogaster*
GCA_902639925	A	Arthropod	*Drosophila melanogaster*
GCA_902639935	A	Arthropod	*Drosophila melanogaster*
GCA_902639945	A	Arthropod	*Drosophila melanogaster*
GCA_902639955	A	Arthropod	*Drosophila melanogaster*
GCA_902639965	A	Arthropod	*Drosophila melanogaster*
GCA_902639975	A	Arthropod	*Drosophila melanogaster*
GCA_902639985	A	Arthropod	*Drosophila melanogaster*
GCA_902639995	A	Arthropod	*Drosophila melanogaster*
GCA_902640005	A	Arthropod	*Drosophila melanogaster*
GCA_902640015	A	Arthropod	*Drosophila melanogaster*
GCA_902640025	A	Arthropod	*Drosophila melanogaster*
GCA_902640035	A	Arthropod	*Drosophila melanogaster*
GCA_902640045	A	Arthropod	*Drosophila melanogaster*
GCA_902640055	A	Arthropod	*Drosophila melanogaster*
GCA_902640065	A	Arthropod	*Drosophila melanogaster*
GCA_902640075	A	Arthropod	*Drosophila melanogaster*
GCA_902640085	A	Arthropod	*Drosophila melanogaster*
GCA_902640095	A	Arthropod	*Drosophila melanogaster*
GCA_902640635	A	Arthropod	*Drosophila melanogaster*
GCA_902640645	A	Arthropod	*Drosophila melanogaster*
GCA_902640655	A	Arthropod	*Drosophila melanogaster*
GCA_902640665	A	Arthropod	*Drosophila melanogaster*
GCA_902640675	A	Arthropod	*Drosophila melanogaster*
GCA_902640685	A	Arthropod	*Drosophila melanogaster*
GCA_902640695	A	Arthropod	*Drosophila melanogaster*
GCA_902640705	A	Arthropod	*Drosophila melanogaster*
GCA_902640715	A	Arthropod	*Drosophila melanogaster*
GCA_902640725	A	Arthropod	*Drosophila melanogaster*
GCA_902640735	A	Arthropod	*Drosophila melanogaster*
GCA_902640745	A	Arthropod	*Drosophila melanogaster*
GCA_902640755	A	Arthropod	*Drosophila melanogaster*
GCA_902640765	A	Arthropod	*Drosophila melanogaster*

GCA_902640775	A	Arthropod	*Drosophila melanogaster*
GCA_902640785	A	Arthropod	*Drosophila melanogaster*
GCA_902640795	A	Arthropod	*Drosophila melanogaster*
GCA_902640805	A	Arthropod	*Drosophila melanogaster*
GCA_902640815	A	Arthropod	*Drosophila melanogaster*
GCA_902640825	A	Arthropod	*Drosophila melanogaster*
GCA_902640835	A	Arthropod	*Drosophila melanogaster*
GCA_902640845	A	Arthropod	*Drosophila melanogaster*
GCA_902640855	A	Arthropod	*Drosophila melanogaster*
GCA_902640865	A	Arthropod	*Drosophila melanogaster*
GCA_902640875	A	Arthropod	*Drosophila melanogaster*
GCA_902640885	A	Arthropod	*Drosophila melanogaster*
GCA_902640895	A	Arthropod	*Drosophila melanogaster*
GCA_902640905	A	Arthropod	*Drosophila melanogaster*
GCA_902640915	A	Arthropod	*Drosophila melanogaster*
GCA_902640925	A	Arthropod	*Drosophila melanogaster*
GCA_902640935	A	Arthropod	*Drosophila melanogaster*
GCA_902640945	A	Arthropod	*Drosophila melanogaster*
GCA_902640955	A	Arthropod	*Drosophila melanogaster*
GCA_902640965	A	Arthropod	*Drosophila melanogaster*
GCA_902640975	A	Arthropod	*Drosophila melanogaster*
GCA_902640985	A	Arthropod	*Drosophila melanogaster*
GCA_902640995	A	Arthropod	*Drosophila melanogaster*
GCA_902641005	A	Arthropod	*Drosophila melanogaster*
GCA_902641015	A	Arthropod	*Drosophila melanogaster*
GCA_902641025	A	Arthropod	*Drosophila melanogaster*
GCA_902641035	A	Arthropod	*Drosophila melanogaster*
GCA_902641045	A	Arthropod	*Drosophila melanogaster*
GCA_902641055	A	Arthropod	*Drosophila melanogaster*
GCA_902641065	A	Arthropod	*Drosophila melanogaster*
GCA_902641075	A	Arthropod	*Drosophila melanogaster*
GCA_902641085	A	Arthropod	*Drosophila melanogaster*
GCA_902641095	A	Arthropod	*Drosophila melanogaster*
GCA_902641105	A	Arthropod	*Drosophila melanogaster*
GCA_902641115	A	Arthropod	*Drosophila melanogaster*
GCA_902641125	A	Arthropod	*Drosophila melanogaster*
GCA_902641135	A	Arthropod	*Drosophila melanogaster*
GCA_902641145	A	Arthropod	*Drosophila melanogaster*
GCA_902641155	A	Arthropod	*Drosophila melanogaster*
GCA_902641165	A	Arthropod	*Drosophila melanogaster*
GCA_902641175	A	Arthropod	*Drosophila melanogaster*
GCA_902641185	A	Arthropod	*Drosophila melanogaster*
GCA_902641195	A	Arthropod	*Drosophila melanogaster*
GCA_902641205	A	Arthropod	*Drosophila melanogaster*
GCA_902641215	A	Arthropod	*Drosophila melanogaster*
GCA_902641225	A	Arthropod	*Drosophila melanogaster*
GCA_902641235	A	Arthropod	*Drosophila melanogaster*
GCA_902641245	A	Arthropod	*Drosophila melanogaster*
GCA_902641255	A	Arthropod	*Drosophila melanogaster*
GCA_902641265	A	Arthropod	*Drosophila melanogaster*
GCA_902641275	A	Arthropod	*Drosophila melanogaster*
GCA_902641285	A	Arthropod	*Drosophila melanogaster*
GCA_902641295	A	Arthropod	*Drosophila melanogaster*
GCA_902641305	A	Arthropod	*Drosophila melanogaster*
GCA_902641315	A	Arthropod	*Drosophila melanogaster*
GCA_902641325	A	Arthropod	*Drosophila melanogaster*
GCA_902641335	A	Arthropod	*Drosophila melanogaster*
GCA_902641345	A	Arthropod	*Drosophila melanogaster*
GCA_902641355	A	Arthropod	*Drosophila melanogaster*

GCA_902641365	A	Arthropod	*Drosophila melanogaster*
GCA_902641375	A	Arthropod	*Drosophila melanogaster*
GCA_902641385	A	Arthropod	*Drosophila melanogaster*
GCA_902641395	A	Arthropod	*Drosophila melanogaster*
GCA_902641405	A	Arthropod	*Drosophila melanogaster*
GCA_902641415	A	Arthropod	*Drosophila melanogaster*
GCA_902641425	A	Arthropod	*Drosophila melanogaster*
GCA_902641435	A	Arthropod	*Drosophila melanogaster*
GCA_902641445	A	Arthropod	*Drosophila melanogaster*
GCA_902641455	A	Arthropod	*Drosophila melanogaster*
GCA_902641465	A	Arthropod	*Drosophila melanogaster*
GCA_902641475	A	Arthropod	*Drosophila melanogaster*
GCA_902641485	A	Arthropod	*Drosophila melanogaster*
GCA_902641495	A	Arthropod	*Drosophila melanogaster*
GCA_902641505	A	Arthropod	*Drosophila melanogaster*
GCA_902641515	A	Arthropod	*Drosophila melanogaster*
GCA_902641525	A	Arthropod	*Drosophila melanogaster*
GCA_902641535	A	Arthropod	*Drosophila melanogaster*
GCA_902641545	A	Arthropod	*Drosophila melanogaster*
GCA_902641555	A	Arthropod	*Drosophila melanogaster*
GCA_902641565	A	Arthropod	*Drosophila melanogaster*
GCA_902641575	A	Arthropod	*Drosophila melanogaster*
GCA_902641585	A	Arthropod	*Drosophila melanogaster*
GCA_902641595	A	Arthropod	*Drosophila melanogaster*
GCA_902641605	A	Arthropod	*Drosophila melanogaster*
GCA_902641615	A	Arthropod	*Drosophila melanogaster*
GCA_902641625	A	Arthropod	*Drosophila melanogaster*
GCA_902641635	A	Arthropod	*Drosophila melanogaster*
GCA_902641645	A	Arthropod	*Drosophila melanogaster*
GCA_902641655	A	Arthropod	*Drosophila melanogaster*
GCA_902641665	A	Arthropod	*Drosophila melanogaster*
GCA_902641675	A	Arthropod	*Drosophila melanogaster*
GCA_902641685	A	Arthropod	*Drosophila melanogaster*
GCA_902641695	A	Arthropod	*Drosophila melanogaster*
GCA_902641705	A	Arthropod	*Drosophila melanogaster*
GCA_902641715	A	Arthropod	*Drosophila melanogaster*
GCA_902641725	A	Arthropod	*Drosophila melanogaster*
GCA_902641735	A	Arthropod	*Drosophila melanogaster*
GCA_902641745	A	Arthropod	*Drosophila melanogaster*
GCA_902641755	A	Arthropod	*Drosophila melanogaster*
GCA_902641765	A	Arthropod	*Drosophila melanogaster*
GCA_902641775	A	Arthropod	*Drosophila melanogaster*
GCA_902641785	A	Arthropod	*Drosophila melanogaster*
GCA_902641795	A	Arthropod	*Drosophila melanogaster*
GCA_902641805	A	Arthropod	*Drosophila melanogaster*
GCA_902641815	A	Arthropod	*Drosophila melanogaster*
GCA_902641825	A	Arthropod	*Drosophila melanogaster*
GCA_902641835	A	Arthropod	*Drosophila melanogaster*
GCA_902641845	A	Arthropod	*Drosophila melanogaster*
GCA_902641855	A	Arthropod	*Drosophila melanogaster*
GCA_902641865	A	Arthropod	*Drosophila melanogaster*
GCA_902641875	A	Arthropod	*Drosophila melanogaster*
GCA_902641885	A	Arthropod	*Drosophila melanogaster*
GCA_902641895	A	Arthropod	*Drosophila melanogaster*
GCA_902641905	A	Arthropod	*Drosophila melanogaster*
GCA_902641915	A	Arthropod	*Drosophila melanogaster*
GCA_902641925	A	Arthropod	*Drosophila melanogaster*
GCA_902641935	A	Arthropod	*Drosophila melanogaster*
GCA_902641945	A	Arthropod	*Drosophila melanogaster*

GCA_902641955	A	Arthropod	*Drosophila melanogaster*
GCA_902641965	A	Arthropod	*Drosophila melanogaster*
GCA_902641975	A	Arthropod	*Drosophila melanogaster*
GCA_902641985	A	Arthropod	*Drosophila melanogaster*
GCA_902641995	A	Arthropod	*Drosophila melanogaster*
GCA_902642005	A	Arthropod	*Drosophila melanogaster*
GCA_902642015	A	Arthropod	*Drosophila melanogaster*
GCA_902642025	A	Arthropod	*Drosophila melanogaster*
GCA_902642035	A	Arthropod	*Drosophila melanogaster*
GCA_902642045	A	Arthropod	*Drosophila melanogaster*
GCA_902642055	A	Arthropod	*Drosophila melanogaster*
GCA_902642065	A	Arthropod	*Drosophila melanogaster*
GCA_902642075	A	Arthropod	*Drosophila melanogaster*
GCA_902642085	A	Arthropod	*Drosophila melanogaster*
GCA_902642095	A	Arthropod	*Drosophila melanogaster*
GCA_902642105	A	Arthropod	*Drosophila melanogaster*
GCA_902642115	A	Arthropod	*Drosophila melanogaster*
GCA_902642125	A	Arthropod	*Drosophila melanogaster*
GCA_902642135	A	Arthropod	*Drosophila melanogaster*
GCA_902642145	A	Arthropod	*Drosophila melanogaster*
GCA_902642155	A	Arthropod	*Drosophila melanogaster*
GCA_902642165	A	Arthropod	*Drosophila melanogaster*
GCA_902642175	A	Arthropod	*Drosophila melanogaster*
GCA_902642185	A	Arthropod	*Drosophila melanogaster*
GCA_902642195	A	Arthropod	*Drosophila melanogaster*
GCA_902642205	A	Arthropod	*Drosophila melanogaster*
GCA_902642215	A	Arthropod	*Drosophila melanogaster*
GCA_902642225	A	Arthropod	*Drosophila melanogaster*
GCA_902642235	A	Arthropod	*Drosophila melanogaster*
GCA_902642245	A	Arthropod	*Drosophila melanogaster*
GCA_902642255	A	Arthropod	*Drosophila melanogaster*
GCA_902642265	A	Arthropod	*Drosophila melanogaster*
GCA_902642275	A	Arthropod	*Drosophila melanogaster*
GCA_902642285	A	Arthropod	*Drosophila melanogaster*
GCA_902642295	A	Arthropod	*Drosophila melanogaster*
GCA_902642305	A	Arthropod	*Drosophila melanogaster*
GCA_902642315	A	Arthropod	*Drosophila melanogaster*
GCA_902642325	A	Arthropod	*Drosophila melanogaster*
GCA_902642335	A	Arthropod	*Drosophila melanogaster*
GCA_902642345	A	Arthropod	*Drosophila melanogaster*
GCA_902642355	A	Arthropod	*Drosophila melanogaster*
GCA_902642365	A	Arthropod	*Drosophila melanogaster*
GCA_902642375	A	Arthropod	*Drosophila melanogaster*
GCA_902642385	A	Arthropod	*Drosophila melanogaster*
GCA_902642395	A	Arthropod	*Drosophila melanogaster*
GCA_902642405	A	Arthropod	*Drosophila melanogaster*
GCA_902642415	A	Arthropod	*Drosophila melanogaster*
GCA_902642425	A	Arthropod	*Drosophila melanogaster*
GCA_902642435	A	Arthropod	*Drosophila melanogaster*
GCA_902642445	A	Arthropod	*Drosophila melanogaster*
GCA_902642455	A	Arthropod	*Drosophila melanogaster*
GCA_902642465	A	Arthropod	*Drosophila melanogaster*
GCA_902642475	A	Arthropod	*Drosophila melanogaster*
GCA_902642485	A	Arthropod	*Drosophila melanogaster*
GCA_902642495	A	Arthropod	*Drosophila melanogaster*
GCA_902642505	A	Arthropod	*Drosophila melanogaster*
GCA_902642515	A	Arthropod	*Drosophila melanogaster*
GCA_902642525	A	Arthropod	*Drosophila melanogaster*
GCA_902642535	A	Arthropod	*Drosophila melanogaster*

GCA_902642545	A	Arthropod	*Drosophila melanogaster*
GCA_902642555	A	Arthropod	*Drosophila melanogaster*
GCA_902642565	A	Arthropod	*Drosophila melanogaster*
GCA_902642575	A	Arthropod	*Drosophila melanogaster*
GCA_902642585	A	Arthropod	*Drosophila melanogaster*
GCA_902642595	A	Arthropod	*Drosophila melanogaster*
GCA_902642605	A	Arthropod	*Drosophila melanogaster*
GCA_902642615	A	Arthropod	*Drosophila melanogaster*
GCA_902642625	A	Arthropod	*Drosophila melanogaster*
GCA_902642635	A	Arthropod	*Drosophila melanogaster*
GCA_902642645	A	Arthropod	*Drosophila melanogaster*
GCA_902642655	A	Arthropod	*Drosophila melanogaster*
GCA_902642665	A	Arthropod	*Drosophila melanogaster*
GCA_902642675	A	Arthropod	*Drosophila melanogaster*
GCA_902642685	A	Arthropod	*Drosophila melanogaster*
GCA_902642695	A	Arthropod	*Drosophila melanogaster*
GCA_902642705	A	Arthropod	*Drosophila melanogaster*
GCA_902642715	A	Arthropod	*Drosophila melanogaster*
GCA_902642725	A	Arthropod	*Drosophila melanogaster*
GCA_902642735	A	Arthropod	*Drosophila melanogaster*
GCA_902642745	A	Arthropod	*Drosophila melanogaster*
GCA_902642755	A	Arthropod	*Drosophila melanogaster*
GCA_902642765	A	Arthropod	*Drosophila melanogaster*
GCA_902642775	A	Arthropod	*Drosophila melanogaster*
GCA_902642785	A	Arthropod	*Drosophila melanogaster*
GCA_902642795	A	Arthropod	*Drosophila melanogaster*
GCA_902642805	A	Arthropod	*Drosophila melanogaster*
GCA_902642815	A	Arthropod	*Drosophila melanogaster*
GCA_902642825	A	Arthropod	*Drosophila melanogaster*
GCA_902642835	A	Arthropod	*Drosophila melanogaster*
GCA_902642845	A	Arthropod	*Drosophila melanogaster*
GCA_902642855	A	Arthropod	*Drosophila melanogaster*
GCA_902642865	A	Arthropod	*Drosophila melanogaster*
GCA_902642875	A	Arthropod	*Drosophila melanogaster*
GCA_902642885	A	Arthropod	*Drosophila melanogaster*
GCA_902642895	A	Arthropod	*Drosophila melanogaster*
GCA_902642905	A	Arthropod	*Drosophila melanogaster*
GCA_902642915	A	Arthropod	*Drosophila melanogaster*
GCA_902642925	A	Arthropod	*Drosophila melanogaster*
GCA_902642935	A	Arthropod	*Drosophila melanogaster*
GCA_902642945	A	Arthropod	*Drosophila melanogaster*
GCA_902642955	A	Arthropod	*Drosophila melanogaster*
GCA_902642965	A	Arthropod	*Drosophila melanogaster*
GCA_902642975	A	Arthropod	*Drosophila melanogaster*
GCA_902642985	A	Arthropod	*Drosophila melanogaster*
GCA_902642995	A	Arthropod	*Drosophila melanogaster*
GCA_902643005	A	Arthropod	*Drosophila melanogaster*
GCA_902643015	A	Arthropod	*Drosophila melanogaster*
GCA_902643025	A	Arthropod	*Drosophila melanogaster*
GCA_902643035	A	Arthropod	*Drosophila melanogaster*
GCA_902643045	A	Arthropod	*Drosophila melanogaster*
GCA_902643055	A	Arthropod	*Drosophila melanogaster*
GCA_902643065	A	Arthropod	*Drosophila melanogaster*
GCA_902643075	A	Arthropod	*Drosophila melanogaster*
GCA_902643085	A	Arthropod	*Drosophila melanogaster*
GCA_902643095	A	Arthropod	*Drosophila melanogaster*
GCA_902643105	A	Arthropod	*Drosophila melanogaster*
GCA_902643115	A	Arthropod	*Drosophila melanogaster*
GCA_902643125	A	Arthropod	*Drosophila melanogaster*

GCA_902643135	A	Arthropod	*Drosophila melanogaster*
GCA_902643145	A	Arthropod	*Drosophila melanogaster*
GCA_902643155	A	Arthropod	*Drosophila melanogaster*
GCA_902643165	A	Arthropod	*Drosophila melanogaster*
GCA_902643175	A	Arthropod	*Drosophila melanogaster*
GCA_902643185	A	Arthropod	*Drosophila melanogaster*
GCA_902643195	A	Arthropod	*Drosophila melanogaster*
GCA_902643205	A	Arthropod	*Drosophila melanogaster*
GCA_902643215	A	Arthropod	*Drosophila melanogaster*
GCA_902643225	A	Arthropod	*Drosophila melanogaster*
GCA_902643235	A	Arthropod	*Drosophila melanogaster*
GCA_902643245	A	Arthropod	*Drosophila melanogaster*
GCA_902643255	A	Arthropod	*Drosophila melanogaster*
GCA_902643265	A	Arthropod	*Drosophila melanogaster*
GCA_902643275	A	Arthropod	*Drosophila melanogaster*
GCA_902643285	A	Arthropod	*Drosophila melanogaster*
GCA_902643295	A	Arthropod	*Drosophila melanogaster*
GCA_902643305	A	Arthropod	*Drosophila melanogaster*
GCA_902643315	A	Arthropod	*Drosophila melanogaster*
GCA_902643325	A	Arthropod	*Drosophila melanogaster*
GCA_902643335	A	Arthropod	*Drosophila melanogaster*
GCA_902643345	A	Arthropod	*Drosophila melanogaster*
GCA_902643355	A	Arthropod	*Drosophila melanogaster*
GCA_902643365	A	Arthropod	*Drosophila melanogaster*
GCA_902643375	A	Arthropod	*Drosophila melanogaster*
GCA_902643385	A	Arthropod	*Drosophila melanogaster*
GCA_902643395	A	Arthropod	*Drosophila melanogaster*
GCA_902643405	A	Arthropod	*Drosophila melanogaster*
GCA_902643415	A	Arthropod	*Drosophila melanogaster*
GCA_902643425	A	Arthropod	*Drosophila melanogaster*
GCA_902643435	A	Arthropod	*Drosophila melanogaster*
GCA_902643445	A	Arthropod	*Drosophila melanogaster*
GCA_902643465	A	Arthropod	*Drosophila melanogaster*
GCA_902643475	A	Arthropod	*Drosophila melanogaster*
GCA_902643485	A	Arthropod	*Drosophila melanogaster*
GCA_902643505	A	Arthropod	*Drosophila melanogaster*
GCA_902643515	A	Arthropod	*Drosophila melanogaster*
GCA_902643525	A	Arthropod	*Drosophila melanogaster*
GCA_902643535	A	Arthropod	*Drosophila melanogaster*
GCA_902643545	A	Arthropod	*Drosophila melanogaster*
GCA_902643555	A	Arthropod	*Drosophila melanogaster*
GCA_902643565	A	Arthropod	*Drosophila melanogaster*
GCA_902643575	A	Arthropod	*Drosophila melanogaster*
GCA_902643585	A	Arthropod	*Drosophila melanogaster*
GCA_902643595	A	Arthropod	*Drosophila melanogaster*
GCA_902643605	A	Arthropod	*Drosophila melanogaster*
GCA_902643615	A	Arthropod	*Drosophila melanogaster*
GCA_902643625	A	Arthropod	*Drosophila melanogaster*
GCA_902643635	A	Arthropod	*Drosophila melanogaster*
GCA_902643645	A	Arthropod	*Drosophila melanogaster*
GCA_902643655	A	Arthropod	*Drosophila melanogaster*
GCA_902643665	A	Arthropod	*Drosophila melanogaster*
GCA_902643755	A	Arthropod	*Drosophila melanogaster*
GCA_902643765	A	Arthropod	*Drosophila melanogaster*
GCA_902643865	A	Arthropod	*Drosophila melanogaster*
GCA_902646145	A	Arthropod	*Drosophila melanogaster*
GCA_902646155	A	Arthropod	*Drosophila melanogaster*
GCA_902646205	A	Arthropod	*Drosophila melanogaster*
GCA_902646225	A	Arthropod	*Drosophila melanogaster*

GCA_902646965	A	Arthropod	*Drosophila melanogaster*
GCA_902647025	A	Arthropod	*Drosophila melanogaster*
GCA_902647035	A	Arthropod	*Drosophila melanogaster*
GCA_902647045	A	Arthropod	*Drosophila melanogaster*
GCA_902647055	A	Arthropod	*Drosophila melanogaster*
GCA_902647065	A	Arthropod	*Drosophila melanogaster*
GCA_902647075	A	Arthropod	*Drosophila melanogaster*
GCA_902647085	A	Arthropod	*Drosophila melanogaster*
GCA_902647095	A	Arthropod	*Drosophila melanogaster*
GCA_902647105	A	Arthropod	*Drosophila melanogaster*
GCA_902647115	A	Arthropod	*Drosophila melanogaster*
GCA_902647125	A	Arthropod	*Drosophila melanogaster*
GCA_902647135	A	Arthropod	*Drosophila melanogaster*
GCA_902647145	A	Arthropod	*Drosophila melanogaster*
GCA_902647155	A	Arthropod	*Drosophila melanogaster*
GCA_902647165	A	Arthropod	*Drosophila melanogaster*
GCA_902647175	A	Arthropod	*Drosophila melanogaster*
GCA_902647185	A	Arthropod	*Drosophila melanogaster*
GCA_902647195	A	Arthropod	*Drosophila melanogaster*
GCA_902647235	A	Arthropod	*Drosophila melanogaster*
GCA_902647245	A	Arthropod	*Drosophila melanogaster*
GCA_902647255	A	Arthropod	*Drosophila melanogaster*
GCA_947533255	A	Arthropod	*Drosophila melanogaster*
GCA_947538885	A	Arthropod	*Drosophila melanogaster*
GCA_014129535	A	Arthropod	*Drosophila neotestacea*
GCA_014107455	A	Arthropod	*Drosophila nikananu*
GCA_028981865	A	Arthropod	*Drosophila nikananu*
GCA_014129565	A	Arthropod	*Drosophila orientacea*
GCA_026015925	A	Arthropod	*Drosophila pseudotakahashii*
GCA_026274225	A	Arthropod	*Drosophila pseudotakahashii*
GCA_000742435	A	Arthropod	*Drosophila recens*
GCA_902643695	A	Arthropod	*Drosophila recens*
GCA_005862095	A	Arthropod	*Drosophila santomea*
GCA_902643735	A	Arthropod	*Drosophila santomea*
GCA_014354315	A	Arthropod	*Drosophila sechellia*
GCA_028981765	A	Arthropod	*Drosophila seguyi*
GCA_902646345	A	Arthropod	*Drosophila simulans*
GCA_000953315	A	Arthropod	*Drosophila simulans*
GCA_011090435	A	Arthropod	*Drosophila simulans*
GCA_902643675	A	Arthropod	*Drosophila simulans*
GCA_902643685	A	Arthropod	*Drosophila simulans*
GCA_902643705	A	Arthropod	*Drosophila simulans*
GCA_902643715	A	Arthropod	*Drosophila simulans*
GCA_902643725	A	Arthropod	*Drosophila simulans*
GCA_902643745	A	Arthropod	*Drosophila simulans*
GCA_902643775	A	Arthropod	*Drosophila simulans*
GCA_902643785	A	Arthropod	*Drosophila simulans*
GCA_902643795	A	Arthropod	*Drosophila simulans*
GCA_902643805	A	Arthropod	*Drosophila simulans*
GCA_902643815	A	Arthropod	*Drosophila simulans*
GCA_902643825	A	Arthropod	*Drosophila simulans*
GCA_902643835	A	Arthropod	*Drosophila simulans*
GCA_902643845	A	Arthropod	*Drosophila simulans*
GCA_902643855	A	Arthropod	*Drosophila simulans*
GCA_902643875	A	Arthropod	*Drosophila simulans*
GCA_902643885	A	Arthropod	*Drosophila simulans*
GCA_902643895	A	Arthropod	*Drosophila simulans*
GCA_902643905	A	Arthropod	*Drosophila simulans*
GCA_902643915	A	Arthropod	*Drosophila simulans*

GCA_902643925	A	Arthropod	*Drosophila simulans*
GCA_902643935	A	Arthropod	*Drosophila simulans*
GCA_902643945	A	Arthropod	*Drosophila simulans*
GCA_902643955	A	Arthropod	*Drosophila simulans*
GCA_902643965	A	Arthropod	*Drosophila simulans*
GCA_902643975	A	Arthropod	*Drosophila simulans*
GCA_902643985	A	Arthropod	*Drosophila simulans*
GCA_902643995	A	Arthropod	*Drosophila simulans*
GCA_902644005	A	Arthropod	*Drosophila simulans*
GCA_902644015	A	Arthropod	*Drosophila simulans*
GCA_902644025	A	Arthropod	*Drosophila simulans*
GCA_902644035	A	Arthropod	*Drosophila simulans*
GCA_902644045	A	Arthropod	*Drosophila simulans*
GCA_902644055	A	Arthropod	*Drosophila simulans*
GCA_902644065	A	Arthropod	*Drosophila simulans*
GCA_902644075	A	Arthropod	*Drosophila simulans*
GCA_902644085	A	Arthropod	*Drosophila simulans*
GCA_902644095	A	Arthropod	*Drosophila simulans*
GCA_902644105	A	Arthropod	*Drosophila simulans*
GCA_902644115	A	Arthropod	*Drosophila simulans*
GCA_902644125	A	Arthropod	*Drosophila simulans*
GCA_902644135	A	Arthropod	*Drosophila simulans*
GCA_902644145	A	Arthropod	*Drosophila simulans*
GCA_902644155	A	Arthropod	*Drosophila simulans*
GCA_902644165	A	Arthropod	*Drosophila simulans*
GCA_902644175	A	Arthropod	*Drosophila simulans*
GCA_902644185	A	Arthropod	*Drosophila simulans*
GCA_902644195	A	Arthropod	*Drosophila simulans*
GCA_902644205	A	Arthropod	*Drosophila simulans*
GCA_902644215	A	Arthropod	*Drosophila simulans*
GCA_902644225	A	Arthropod	*Drosophila simulans*
GCA_902644235	A	Arthropod	*Drosophila simulans*
GCA_902644245	A	Arthropod	*Drosophila simulans*
GCA_902644255	A	Arthropod	*Drosophila simulans*
GCA_902644265	A	Arthropod	*Drosophila simulans*
GCA_902644275	A	Arthropod	*Drosophila simulans*
GCA_902644285	A	Arthropod	*Drosophila simulans*
GCA_902644295	A	Arthropod	*Drosophila simulans*
GCA_902644305	A	Arthropod	*Drosophila simulans*
GCA_902644315	A	Arthropod	*Drosophila simulans*
GCA_902644325	A	Arthropod	*Drosophila simulans*
GCA_902644335	A	Arthropod	*Drosophila simulans*
GCA_902644345	A	Arthropod	*Drosophila simulans*
GCA_902644355	A	Arthropod	*Drosophila simulans*
GCA_902644365	A	Arthropod	*Drosophila simulans*
GCA_902644375	A	Arthropod	*Drosophila simulans*
GCA_902644385	A	Arthropod	*Drosophila simulans*
GCA_902644395	A	Arthropod	*Drosophila simulans*
GCA_902644405	A	Arthropod	*Drosophila simulans*
GCA_902644415	A	Arthropod	*Drosophila simulans*
GCA_902644425	A	Arthropod	*Drosophila simulans*
GCA_902644435	A	Arthropod	*Drosophila simulans*
GCA_902644445	A	Arthropod	*Drosophila simulans*
GCA_902644455	A	Arthropod	*Drosophila simulans*
GCA_902644465	A	Arthropod	*Drosophila simulans*
GCA_902644475	A	Arthropod	*Drosophila simulans*
GCA_902644485	A	Arthropod	*Drosophila simulans*
GCA_902644495	A	Arthropod	*Drosophila simulans*
GCA_902644505	A	Arthropod	*Drosophila simulans*

GCA_902644515	A	Arthropod	*Drosophila simulans*
GCA_902644525	A	Arthropod	*Drosophila simulans*
GCA_902644535	A	Arthropod	*Drosophila simulans*
GCA_902644545	A	Arthropod	*Drosophila simulans*
GCA_902644555	A	Arthropod	*Drosophila simulans*
GCA_902644565	A	Arthropod	*Drosophila simulans*
GCA_902644575	A	Arthropod	*Drosophila simulans*
GCA_902644585	A	Arthropod	*Drosophila simulans*
GCA_902644595	A	Arthropod	*Drosophila simulans*
GCA_902644605	A	Arthropod	*Drosophila simulans*
GCA_902644615	A	Arthropod	*Drosophila simulans*
GCA_902644625	A	Arthropod	*Drosophila simulans*
GCA_902644635	A	Arthropod	*Drosophila simulans*
GCA_902644645	A	Arthropod	*Drosophila simulans*
GCA_902644655	A	Arthropod	*Drosophila simulans*
GCA_902644665	A	Arthropod	*Drosophila simulans*
GCA_902644675	A	Arthropod	*Drosophila simulans*
GCA_902644685	A	Arthropod	*Drosophila simulans*
GCA_902644695	A	Arthropod	*Drosophila simulans*
GCA_902644705	A	Arthropod	*Drosophila simulans*
GCA_902644715	A	Arthropod	*Drosophila simulans*
GCA_902644725	A	Arthropod	*Drosophila simulans*
GCA_902644735	A	Arthropod	*Drosophila simulans*
GCA_902644745	A	Arthropod	*Drosophila simulans*
GCA_902644755	A	Arthropod	*Drosophila simulans*
GCA_902644765	A	Arthropod	*Drosophila simulans*
GCA_902644775	A	Arthropod	*Drosophila simulans*
GCA_902644785	A	Arthropod	*Drosophila simulans*
GCA_902644795	A	Arthropod	*Drosophila simulans*
GCA_902644805	A	Arthropod	*Drosophila simulans*
GCA_902644815	A	Arthropod	*Drosophila simulans*
GCA_902644825	A	Arthropod	*Drosophila simulans*
GCA_902644835	A	Arthropod	*Drosophila simulans*
GCA_902644845	A	Arthropod	*Drosophila simulans*
GCA_902644855	A	Arthropod	*Drosophila simulans*
GCA_902644865	A	Arthropod	*Drosophila simulans*
GCA_902644875	A	Arthropod	*Drosophila simulans*
GCA_902644885	A	Arthropod	*Drosophila simulans*
GCA_902644895	A	Arthropod	*Drosophila simulans*
GCA_902644905	A	Arthropod	*Drosophila simulans*
GCA_902644915	A	Arthropod	*Drosophila simulans*
GCA_902644925	A	Arthropod	*Drosophila simulans*
GCA_902644935	A	Arthropod	*Drosophila simulans*
GCA_902644945	A	Arthropod	*Drosophila simulans*
GCA_902644955	A	Arthropod	*Drosophila simulans*
GCA_902644965	A	Arthropod	*Drosophila simulans*
GCA_902644975	A	Arthropod	*Drosophila simulans*
GCA_902644985	A	Arthropod	*Drosophila simulans*
GCA_902644995	A	Arthropod	*Drosophila simulans*
GCA_902645005	A	Arthropod	*Drosophila simulans*
GCA_902645015	A	Arthropod	*Drosophila simulans*
GCA_902645025	A	Arthropod	*Drosophila simulans*
GCA_902645035	A	Arthropod	*Drosophila simulans*
GCA_902645045	A	Arthropod	*Drosophila simulans*
GCA_902645055	A	Arthropod	*Drosophila simulans*
GCA_902645065	A	Arthropod	*Drosophila simulans*
GCA_902645075	A	Arthropod	*Drosophila simulans*
GCA_902645085	A	Arthropod	*Drosophila simulans*
GCA_902645095	A	Arthropod	*Drosophila simulans*

GCA_902645105	A	Arthropod	*Drosophila simulans*
GCA_902645115	A	Arthropod	*Drosophila simulans*
GCA_902645125	A	Arthropod	*Drosophila simulans*
GCA_902645135	A	Arthropod	*Drosophila simulans*
GCA_902645145	A	Arthropod	*Drosophila simulans*
GCA_902645155	A	Arthropod	*Drosophila simulans*
GCA_902645165	A	Arthropod	*Drosophila simulans*
GCA_902645175	A	Arthropod	*Drosophila simulans*
GCA_902645185	A	Arthropod	*Drosophila simulans*
GCA_902645195	A	Arthropod	*Drosophila simulans*
GCA_902645205	A	Arthropod	*Drosophila simulans*
GCA_902645215	A	Arthropod	*Drosophila simulans*
GCA_902645225	A	Arthropod	*Drosophila simulans*
GCA_902645235	A	Arthropod	*Drosophila simulans*
GCA_902645245	A	Arthropod	*Drosophila simulans*
GCA_902645255	A	Arthropod	*Drosophila simulans*
GCA_902645265	A	Arthropod	*Drosophila simulans*
GCA_902645275	A	Arthropod	*Drosophila simulans*
GCA_902645285	A	Arthropod	*Drosophila simulans*
GCA_902645295	A	Arthropod	*Drosophila simulans*
GCA_902645305	A	Arthropod	*Drosophila simulans*
GCA_902645315	A	Arthropod	*Drosophila simulans*
GCA_902645325	A	Arthropod	*Drosophila simulans*
GCA_902645335	A	Arthropod	*Drosophila simulans*
GCA_902645345	A	Arthropod	*Drosophila simulans*
GCA_902645355	A	Arthropod	*Drosophila simulans*
GCA_902645365	A	Arthropod	*Drosophila simulans*
GCA_902645375	A	Arthropod	*Drosophila simulans*
GCA_902645385	A	Arthropod	*Drosophila simulans*
GCA_902645395	A	Arthropod	*Drosophila simulans*
GCA_902645405	A	Arthropod	*Drosophila simulans*
GCA_902645415	A	Arthropod	*Drosophila simulans*
GCA_902645425	A	Arthropod	*Drosophila simulans*
GCA_902645435	A	Arthropod	*Drosophila simulans*
GCA_902645445	A	Arthropod	*Drosophila simulans*
GCA_902645455	A	Arthropod	*Drosophila simulans*
GCA_902645465	A	Arthropod	*Drosophila simulans*
GCA_902645475	A	Arthropod	*Drosophila simulans*
GCA_902645485	A	Arthropod	*Drosophila simulans*
GCA_902645495	A	Arthropod	*Drosophila simulans*
GCA_902645505	A	Arthropod	*Drosophila simulans*
GCA_902645515	A	Arthropod	*Drosophila simulans*
GCA_902645525	A	Arthropod	*Drosophila simulans*
GCA_902645535	A	Arthropod	*Drosophila simulans*
GCA_902645545	A	Arthropod	*Drosophila simulans*
GCA_902645555	A	Arthropod	*Drosophila simulans*
GCA_902645565	A	Arthropod	*Drosophila simulans*
GCA_902645575	A	Arthropod	*Drosophila simulans*
GCA_902645585	A	Arthropod	*Drosophila simulans*
GCA_902645595	A	Arthropod	*Drosophila simulans*
GCA_902645605	A	Arthropod	*Drosophila simulans*
GCA_902645615	A	Arthropod	*Drosophila simulans*
GCA_902645625	A	Arthropod	*Drosophila simulans*
GCA_902645635	A	Arthropod	*Drosophila simulans*
GCA_902645645	A	Arthropod	*Drosophila simulans*
GCA_902645655	A	Arthropod	*Drosophila simulans*
GCA_902645665	A	Arthropod	*Drosophila simulans*
GCA_902645675	A	Arthropod	*Drosophila simulans*
GCA_902645685	A	Arthropod	*Drosophila simulans*

GCA_902645695	A	Arthropod	*Drosophila simulans*
GCA_902645705	A	Arthropod	*Drosophila simulans*
GCA_902645715	A	Arthropod	*Drosophila simulans*
GCA_902645725	A	Arthropod	*Drosophila simulans*
GCA_902645735	A	Arthropod	*Drosophila simulans*
GCA_902645745	A	Arthropod	*Drosophila simulans*
GCA_902645755	A	Arthropod	*Drosophila simulans*
GCA_902645765	A	Arthropod	*Drosophila simulans*
GCA_902645775	A	Arthropod	*Drosophila simulans*
GCA_902645785	A	Arthropod	*Drosophila simulans*
GCA_902645795	A	Arthropod	*Drosophila simulans*
GCA_902645805	A	Arthropod	*Drosophila simulans*
GCA_902645815	A	Arthropod	*Drosophila simulans*
GCA_902645825	A	Arthropod	*Drosophila simulans*
GCA_902645835	A	Arthropod	*Drosophila simulans*
GCA_902645845	A	Arthropod	*Drosophila simulans*
GCA_902645855	A	Arthropod	*Drosophila simulans*
GCA_902645865	A	Arthropod	*Drosophila simulans*
GCA_902645875	A	Arthropod	*Drosophila simulans*
GCA_902645885	A	Arthropod	*Drosophila simulans*
GCA_902645895	A	Arthropod	*Drosophila simulans*
GCA_902645905	A	Arthropod	*Drosophila simulans*
GCA_902645915	A	Arthropod	*Drosophila simulans*
GCA_902645925	A	Arthropod	*Drosophila simulans*
GCA_902645935	A	Arthropod	*Drosophila simulans*
GCA_902645945	A	Arthropod	*Drosophila simulans*
GCA_902645955	A	Arthropod	*Drosophila simulans*
GCA_902645965	A	Arthropod	*Drosophila simulans*
GCA_902645975	A	Arthropod	*Drosophila simulans*
GCA_902645985	A	Arthropod	*Drosophila simulans*
GCA_902645995	A	Arthropod	*Drosophila simulans*
GCA_902646005	A	Arthropod	*Drosophila simulans*
GCA_902646015	A	Arthropod	*Drosophila simulans*
GCA_902646025	A	Arthropod	*Drosophila simulans*
GCA_902646035	A	Arthropod	*Drosophila simulans*
GCA_902646045	A	Arthropod	*Drosophila simulans*
GCA_902646055	A	Arthropod	*Drosophila simulans*
GCA_902646245	A	Arthropod	*Drosophila simulans*
GCA_902646265	A	Arthropod	*Drosophila simulans*
GCA_902646275	A	Arthropod	*Drosophila simulans*
GCA_902646295	A	Arthropod	*Drosophila simulans*
GCA_902646305	A	Arthropod	*Drosophila simulans*
GCA_902646315	A	Arthropod	*Drosophila simulans*
GCA_902646355	A	Arthropod	*Drosophila simulans*
GCA_902646365	A	Arthropod	*Drosophila simulans*
GCA_902646395	A	Arthropod	*Drosophila simulans*
GCA_902646435	A	Arthropod	*Drosophila simulans*
GCA_902647445	A	Arthropod	*Drosophila simulans*
GCA_902647455	A	Arthropod	*Drosophila simulans*
GCA_902647465	A	Arthropod	*Drosophila simulans*
GCA_902647475	A	Arthropod	*Drosophila simulans*
GCA_902647485	A	Arthropod	*Drosophila simulans*
GCA_902647495	A	Arthropod	*Drosophila simulans*
GCA_902647505	A	Arthropod	*Drosophila simulans*
GCA_902647515	A	Arthropod	*Drosophila simulans*
GCA_902647525	A	Arthropod	*Drosophila simulans*
GCA_902647535	A	Arthropod	*Drosophila simulans*
GCA_902647545	A	Arthropod	*Drosophila simulans*
GCA_902647555	A	Arthropod	*Drosophila simulans*

GCA_902647565	A	Arthropod	*Drosophila simulans*
GCA_902647575	A	Arthropod	*Drosophila simulans*
GCA_902647585	A	Arthropod	*Drosophila simulans*
GCA_902647595	A	Arthropod	*Drosophila simulans*
GCA_902647605	A	Arthropod	*Drosophila simulans*
GCA_902647615	A	Arthropod	*Drosophila simulans*
GCA_902647625	A	Arthropod	*Drosophila simulans*
GCA_902647635	A	Arthropod	*Drosophila simulans*
GCA_902647645	A	Arthropod	*Drosophila simulans*
GCA_902647655	A	Arthropod	*Drosophila simulans*
GCA_902647665	A	Arthropod	*Drosophila simulans*
GCA_902647675	A	Arthropod	*Drosophila simulans*
GCA_902647685	A	Arthropod	*Drosophila simulans*
GCA_902647695	A	Arthropod	*Drosophila simulans*
GCA_902647705	A	Arthropod	*Drosophila simulans*
GCA_902647715	A	Arthropod	*Drosophila simulans*
GCA_902647725	A	Arthropod	*Drosophila simulans*
GCA_902647735	A	Arthropod	*Drosophila simulans*
GCA_902647745	A	Arthropod	*Drosophila simulans*
GCA_902647755	A	Arthropod	*Drosophila simulans*
GCA_902647765	A	Arthropod	*Drosophila simulans*
GCA_902647775	A	Arthropod	*Drosophila simulans*
GCA_902647785	A	Arthropod	*Drosophila simulans*
GCA_902647795	A	Arthropod	*Drosophila simulans*
GCA_902647805	A	Arthropod	*Drosophila simulans*
GCA_902647815	A	Arthropod	*Drosophila simulans*
GCA_902647825	A	Arthropod	*Drosophila simulans*
GCA_902647835	A	Arthropod	*Drosophila simulans*
GCA_902647845	A	Arthropod	*Drosophila simulans*
GCA_902647855	A	Arthropod	*Drosophila simulans*
GCA_902647865	A	Arthropod	*Drosophila simulans*
GCA_902647875	A	Arthropod	*Drosophila simulans*
GCA_902647885	A	Arthropod	*Drosophila simulans*
GCA_902647895	A	Arthropod	*Drosophila simulans*
GCA_902647905	A	Arthropod	*Drosophila simulans*
GCA_902647915	A	Arthropod	*Drosophila simulans*
GCA_902647925	A	Arthropod	*Drosophila simulans*
GCA_902647935	A	Arthropod	*Drosophila simulans*
GCA_902647945	A	Arthropod	*Drosophila simulans*
GCA_902647955	A	Arthropod	*Drosophila simulans*
GCA_902647965	A	Arthropod	*Drosophila simulans*
GCA_902647975	A	Arthropod	*Drosophila simulans*
GCA_902647985	A	Arthropod	*Drosophila simulans*
GCA_902647995	A	Arthropod	*Drosophila simulans*
GCA_902648005	A	Arthropod	*Drosophila simulans*
GCA_902648015	A	Arthropod	*Drosophila simulans*
GCA_902648025	A	Arthropod	*Drosophila simulans*
GCA_902648035	A	Arthropod	*Drosophila simulans*
GCA_902648045	A	Arthropod	*Drosophila simulans*
GCA_902648065	A	Arthropod	*Drosophila simulans*
GCA_902648075	A	Arthropod	*Drosophila simulans*
GCA_902648085	A	Arthropod	*Drosophila simulans*
GCA_902648105	A	Arthropod	*Drosophila simulans*
GCA_902648125	A	Arthropod	*Drosophila simulans*
GCA_902648135	A	Arthropod	*Drosophila simulans*
GCA_000022285	A	Arthropod	*Drosophila simulans Riverside*
GCA_018467115	A	Arthropod	*Drosophila simulans STC*
GCA_018467135	A	Arthropod	*Drosophila simulans STC*
GCA_018689955	A	Arthropod	*Drosophila simulans STC*

GCA_018690095	A	Arthropod	*Drosophila simulans STC*
GCA_000376605	A	Arthropod	*Drosophila simulans wHa*
GCA_014107475	A	Arthropod	*Drosophila sturtevanti*
GCA_002300525	A	Arthropod	*Drosophila subpulchrella*
GCA_000333795	A	Arthropod	*Drosophila suzukii*
GCA_902646325	A	Arthropod	*Drosophila suzukii*
GCA_902646415	A	Arthropod	*Drosophila suzukii*
GCA_005862135	A	Arthropod	*Drosophila teissieri*
GCA_014129515	A	Arthropod	*Drosophila triauraria*
GCA_028981745	A	Arthropod	*Drosophila tristis*
GCA_014129525	A	Arthropod	*Drosophila tropicalis*
GCA_028982115	A	Arthropod	*Drosophila tsacasi*
GCA_005862115	A	Arthropod	*Drosophila yakuba*
GCA_902646285	A	Arthropod	*Drosophila yakuba*
GCA_902646385	A	Arthropod	*Drosophila yakuba*
GCA_902636355	A	Arthropod	*Dufourea novaeangliae*
GCA_902636375	A	Arthropod	*Dufourea novaeangliae*
GCA_902636445	A	Arthropod	*Dufourea novaeangliae*
GCA_902636465	A	Arthropod	*Dufourea novaeangliae*
GCA_947251695	A	Arthropod	*Ectemnius continuus*
GCA_947250805	A	Arthropod	*Endotricha flammealis*
GCA_947251775	A	Arthropod	*Epagoge grotiana*
GCA_947251745	A	Arthropod	*Epagoge grotiana*
GCA_947251475	A	Arthropod	*Epirrhoe alternata*
GCA_947251435	A	Arthropod	*Epistrophe grossularia*
GCA_947251925	A	Arthropod	*Epistrophe grossularia*
GCA_947251595	A	Arthropod	*Eupithecia tripunctaria*
GCA_029238795	A	Arthropod	*Eurosta solidaginis*
GCA_000689175	A	Arthropod	*Glossina morsitans*
GCA_947250765	A	Arthropod	*Gymnosoma rotundatum*
GCA_009732755	A	Arthropod	*Haematobia irritans*
GCA_030267405	A	Arthropod	*Homona magnanima*
GCA_030267425	A	Arthropod	*Homona magnanima*
GCA_947251915	A	Arthropod	*Hylaeus communis*
GCA_947251635	A	Arthropod	*Icerya purchasi*
GCA_016031645	A	Arthropod	*Kradibia gibbosae*
GCA_902636435	A	Arthropod	*Lasioglossum albipes*
GCA_902636315	A	Arthropod	*Lasioglossum albipes*
GCA_902636335	A	Arthropod	*Lasioglossum albipes*
GCA_902636405	A	Arthropod	*Lasioglossum albipes*
GCA_902636415	A	Arthropod	*Lasioglossum albipes*
GCA_902636455	A	Arthropod	*Lasioglossum albipes*
GCA_947251945	A	Arthropod	*Lasioglossum lativentre*
GCA_947179405	A	Arthropod	*Lasioglossum malachurum*
GCA_947179495	A	Arthropod	*Lasioglossum malachurum*
GCA_947250585	A	Arthropod	*Lasioglossum morio*
GCA_947250495	A	Arthropod	*Lasioglossum morio*
GCA_947251705	A	Arthropod	*Lasioglossum morio*
GCA_947251555	A	Arthropod	*Macropis europaea*
GCA_947251675	A	Arthropod	*Merzomyia westermanni*
GCA_000174095	A	Arthropod	*Muscidifurax uniraptor*
GCA_001983635	A	Arthropod	*Muscidifurax uniraptor*
GCA_009012935	A	Arthropod	*Nasonia oneida*
GCA_001983615	A	Arthropod	*Nasonia vitripennis*
GCA_947250795	A	Arthropod	*Nomada fabriciana*
GCA_001675785	A	Arthropod	*Nomada ferruginata*
GCA_001675695	A	Arthropod	*Nomada flava*
GCA_001675715	A	Arthropod	*Nomada leucophthalma*
GCA_001675775	A	Arthropod	*Nomada panzeri*

GCA_936270435	A	Arthropod	*Oedothorax gibbosus*
GCA_936270145	A	Arthropod	*Oedothorax gibbosus*
GCA_947250505	A	Arthropod	*Phalera bucephala*
GCA_947251565	A	Arthropod	*Pheosia gnoma*
GCA_947251755	A	Arthropod	*Philonthus cognatus*
GCA_918697765	A	Arthropod	*Phyllotreta cruciferae*
GCA_019097885	A	Arthropod	*Pissodes strobi*
GCA_947179415	A	Arthropod	*Platycheirus albimanus*
GCA_947251425	A	Arthropod	*Protocalliphora azurea*
GCA_918342435	A	Arthropod	*Psylliodes chrysocephala*
GCA_018454475	A	Arthropod	*Rhagoletis cerasi*
GCA_017604245	A	Arthropod	*Rhagoletis cingulata*
GCA_947250775	A	Arthropod	*Rhinocyllus conicus*
GCA_947251485	A	Arthropod	*Scambus nigricans*
GCA_947251515	A	Arthropod	*Sicus ferrugineus*
GCA_947250735	A	Arthropod	*Sphaerophoria taeniata*
GCA_947179545	A	Arthropod	*Sphecodes ephippius*
GCA_947250685	A	Arthropod	*Sphecodes monilicornis*
GCA_947251965	A	Arthropod	*Sphecodes monilicornis*
GCA_902647005	A	Arthropod	*Sphyracephala brevicornis*
GCA_947250715	A	Arthropod	*Sympetrum striolatum*
GCA_947251725	A	Arthropod	*Tiphia femorata*
GCA_947251665	A	Arthropod	*Trypoxylon clavicerum*
GCA_934668665	A	Arthropod	*unclear host - human gut microbiome*
GCA_947250665	A	Arthropod	*Volucella inflata*
GCA_947179175	A	Arthropod	*Volucella inflata*
GCA_017869285	A	Arthropod	*Wiebesia pumilae*
GCA_947251575	A	Arthropod	*Yponomeuta plumbellus*
GCA_019665805	B	Arthropod	*Aedes aegypti*
GCA_025666255	B	Arthropod	*Aedes aegypti*
GCA_000242415	B	Arthropod	*Aedes albopictus*
GCA_002374845	B	Arthropod	*Aedes albopictus*
GCA_002379145	B	Arthropod	*Aedes albopictus*
GCA_004171285	B	Arthropod	*Aedes albopictus*
GCA_004795415	B	Arthropod	*Aedes albopictus*
GCA_902648095	B	Arthropod	*Aedes albopictus*
GCA_947250755	B	Arthropod	*Agriphila straminella*
GCA_947179465	B	Arthropod	*Agriphila tristella*
GCA_018491735	B	Arthropod	*Anopheles demeilloni*
GCA_018491625	B	Arthropod	*Anopheles moucheti*
GCA_947250485	B	Arthropod	*Aporia crataegi*
GCA_947250475	B	Arthropod	*Apotomis betuletana*
GCA_947251835	B	Arthropod	*Apotomis turbidana*
GCA_947250705	B	Arthropod	*Archips podanus*
GCA_947179385	B	Arthropod	*Aricia agestis*
GCA_001027565	B	Arthropod	*Armadillidium vulgare*
GCA_947251845	B	Arthropod	*Athalia cordata*
GCA_003999585	B	Arthropod	*Bemisia tabaci*
GCA_900097055	B	Arthropod	*Bemisia tabaci*
GCA_947250595	B	Arthropod	*Campaea margaritaria*
GCA_947251825	B	Arthropod	*Camptogramma bilineatum*
GCA_947179375	B	Arthropod	*Carcina quercana*
GCA_947250515	B	Arthropod	*Catoptria pinella*
GCA_947251805	B	Arthropod	*Celastrina argiolus*
GCA_947179555	B	Arthropod	*Chorthippus brunneus*
GCA_947251815	B	Arthropod	*Chorthippus parallelus*
GCA_008245065	B	Arthropod	*Chrysomya megacephala*
GCA_947250525	B	Arthropod	*Colias croceus*
GCA_020995475	B	Arthropod	*Corcyra cephalonica*

GCA_000723225	B	Arthropod	*Culex molestus*
GCA_000208785	B	Arthropod	*Culex pipiens molestus*
GCA_000156735	B	Arthropod	*Culex quinquefasciatus JHB*
GCA_000073005	B	Arthropod	*Culex quinquefasciatus Pel*
GCA_003344345	B	Arthropod	*Cylisticus convexus*
GCA_001648015	B	Arthropod	*Dactylopius coccus*
GCA_013458815	B	Arthropod	*Diaphorina citri*
GCA_000331595	B	Arthropod	*Diaphorina citri*
GCA_013096355	B	Arthropod	*Diaphorina citri*
GCA_013096535	B	Arthropod	*Diaphorina citri*
GCA_013096725	B	Arthropod	*Diaphorina citri*
GCA_017883655	B	Arthropod	*Diaphorina citri*
GCA_017883735	B	Arthropod	*Diaphorina citri*
GCA_017883805	B	Arthropod	*Diaphorina citri*
GCA_017883845	B	Arthropod	*Diaphorina citri*
GCA_017883905	B	Arthropod	*Diaphorina citri*
GCA_019355235	B	Arthropod	*Diaphorina citri*
GCA_019355355	B	Arthropod	*Diaphorina citri*
GCA_019355375	B	Arthropod	*Diaphorina citri*
GCA_902636705	B	Arthropod	*Diaphorina citri*
GCA_902636735	B	Arthropod	*Diaphorina citri*
GCA_902636745	B	Arthropod	*Diaphorina citri*
GCA_902636755	B	Arthropod	*Diaphorina citri*
GCA_902636765	B	Arthropod	*Diaphorina citri*
GCA_902636795	B	Arthropod	*Diaphorina citri*
GCA_902636805	B	Arthropod	*Diaphorina citri*
GCA_902636835	B	Arthropod	*Diaphorina citri*
GCA_902636865	B	Arthropod	*Diaphorina citri*
GCA_902646935	B	Arthropod	*Diaphorina citri*
GCA_947179505	B	Arthropod	*Dolichovespula media*
GCA_004685025	B	Arthropod	*Drosophila mauritiana*
GCA_004795955	B	Arthropod	*Drosophila mauritiana*
GCA_004795975	B	Arthropod	*Drosophila mauritiana*
GCA_902646095	B	Arthropod	*Drosophila mauritiana*
GCA_902646115	B	Arthropod	*Drosophila mauritiana*
GCA_902646125	B	Arthropod	*Drosophila mauritiana*
GCA_902646185	B	Arthropod	*Drosophila mauritiana*
GCA_000376585	B	Arthropod	*Drosophila simulans*
GCA_018690035	B	Arthropod	*Drosophila simulans Madagaskar*
GCA_947251605	B	Arthropod	*Emmelina monodactyla*
GCA_947251735	B	Arthropod	*Endotricha flammealis*
GCA_947250575	B	Arthropod	*Episyrphus balteatus*
GCA_018555315	B	Arthropod	*Erebia cassioides*
GCA_947179575	B	Arthropod	*Erebia ligea*
GCA_947250545	B	Arthropod	*Erynnis tages*
GCA_947250645	B	Arthropod	*Eucosma cana*
GCA_947250695	B	Arthropod	*Eupeodes latifasciatus*
GCA_947250535	B	Arthropod	*Euphydryas aurinia*
GCA_029856955	B	Arthropod	*Frankliniella intonsa*
GCA_018224395	B	Arthropod	*Homalodisca vitripennis*
GCA_022865145	B	Arthropod	*Homalodisca vitripennis*
GCA_022867525	B	Arthropod	*Homalodisca vitripennis*
GCA_902646375	B	Arthropod	*Homalodisca vitripennis*
GCA_902646425	B	Arthropod	*Homalodisca vitripennis*
GCA_030295095	B	Arthropod	*Homona magnanima*
GCA_030295115	B	Arthropod	*Homona magnanima*
GCA_947251975	B	Arthropod	*Hylaea fasciaria*
GCA_000333775	B	Arthropod	*Hypolimnas bolina*
GCA_947179535	B	Arthropod	*Idaea aversata*

GCA_947251585	B	Arthropod	*Ischnura elegans*
GCA_007115015	B	Arthropod	*Laodelphax striatellus*
GCA_001637495	B	Arthropod	*Laodelphax striatellus*
GCA_947179455	B	Arthropod	*Leptidea sinapis*
GCA_006334525	B	Arthropod	*Leptopilina clavipes*
GCA_947251875	B	Arthropod	*Lycaena phlaeas*
GCA_947251465	B	Arthropod	*Melanostoma mellinum*
GCA_000204545	B	Arthropod	*Nasonia vitripennis*
GCA_007115045	B	Arthropod	*Nilaparvata lugens*
GCA_947250465	B	Arthropod	*Nymphalis c-album*
GCA_947250815	B	Arthropod	*Nymphalis c-album*
GCA_947179425	B	Arthropod	*Ochlodes sylvanus*
GCA_001266585	B	Arthropod	*Operophtera brumata*
GCA_025617515	B	Arthropod	*Oryzaephilus surinamensis*
GCA_023559125	B	Arthropod	*Ostrinia furnacalis*
GCA_023559145	B	Arthropod	*Ostrinia scapulalis*
GCA_947251495	B	Arthropod	*Pammene fasciana*
GCA_947179145	B	Arthropod	*Pandemis cinnamomeana*
GCA_947250555	B	Arthropod	*Pandemis corylana*
GCA_947250635	B	Arthropod	*Parapoynx stratiotata*
GCA_902636855	B	Arthropod	*Pararge aegeria*
GCA_947250725	B	Arthropod	*Pararge aegeria*
GCA_947251535	B	Arthropod	*Phalera bucephala*
GCA_947251625	B	Arthropod	*Phalera bucephala*
GCA_947251655	B	Arthropod	*Pheosia tremula*
GCA_947179345	B	Arthropod	*Philonthus cognatus*
GCA_002318985	B	Arthropod	*Plutella australiana*
GCA_902636825	B	Arthropod	*Polygonia c-album*
GCA_947250745	B	Arthropod	*Polyommatus icarus*
GCA_947251865	B	Arthropod	*Protocalliphora azurea*
GCA_947250605	B	Arthropod	*Pyrgus malvae*
GCA_018454445	B	Arthropod	*Rhagoletis cerasi*
GCA_947250615	B	Arthropod	*Rhopobota naevana*
GCA_947179355	B	Arthropod	*Sphaerophoria taeniata*
GCA_018141665	B	Arthropod	*Spodoptera picta*
GCA_024205405	B	Arthropod	*Tetranychus truncatus*
GCA_902646255	B	Arthropod	*Tetranychus urticae*
GCA_947251505	B	Arthropod	*Thymelicus sylvestris*
GCA_020405475	B	Arthropod	*Tribolium confusum*
GCA_001439985	B	Arthropod	*Trichogramma pretiosum*
GCA_947250655	B	Arthropod	*Watsonalla binaria*
GCA_947250675	B	Arthropod	*Xestia c-nigrum*
GCA_013365455	C	Filarioidea	*Dirofilaria immitis*
wDrepC	C	Filarioidea	*Dirofilaria repens*
GCA_029169405	C	Filarioidea	*Onchocerca gibsoni*
GCA_902646575	C	Filarioidea	*Onchocerca gutturosa*
GCA_000306885	C	Filarioidea	*Onchocerca ochengi*
GCA_902646545	C	Filarioidea	*Onchocerca ochengi*
GCA_902646555	C	Filarioidea	*Onchocerca ochengi*
GCA_902646585	C	Filarioidea	*Onchocerca ochengi*
GCA_902646605	C	Filarioidea	*Onchocerca ochengi*
GCA_902646625	C	Filarioidea	*Onchocerca ochengi*
GCA_902646645	C	Filarioidea	*Onchocerca ochengi*
GCA_902648145	C	Filarioidea	*Onchocerca ochengi*
GCA_902648165	C	Filarioidea	*Onchocerca volvulus*
GCA_902646785	C	Filarioidea	*Onchocerca volvulus*
GCA_902646815	C	Filarioidea	*Onchocerca volvulus*
GCA_902646835	C	Filarioidea	*Onchocerca volvulus*
GCA_000530755	C	Filarioidea	*Onchocerca volvulus*

GCA_902646725	C	Filarioidea	*Onchocerca volvulus*
GCA_902646735	C	Filarioidea	*Onchocerca volvulus*
GCA_902646745	C	Filarioidea	*Onchocerca volvulus*
GCA_902646755	C	Filarioidea	*Onchocerca volvulus*
GCA_902646765	C	Filarioidea	*Onchocerca volvulus*
GCA_902646775	C	Filarioidea	*Onchocerca volvulus*
GCA_902646795	C	Filarioidea	*Onchocerca volvulus*
GCA_902646805	C	Filarioidea	*Onchocerca volvulus*
GCA_902648055	C	Filarioidea	*Onchocerca volvulus*
GCA_902648195	C	Filarioidea	*Onchocerca volvulus*
GCA_902648215	C	Filarioidea	*Onchocerca volvulus*
GCA_004795935	D	Filarioidea	*Brugia malayi*
GCA_000008385	D	Filarioidea	*Brugia malayi*
GCA_902646335	D	Filarioidea	*Brugia malayi*
GCA_902646405	D	Filarioidea	*Brugia malayi*
GCA_902646445	D	Filarioidea	*Brugia malayi*
GCA_902646455	D	Filarioidea	*Brugia malayi*
GCA_902646465	D	Filarioidea	*Brugia malayi*
GCA_902646475	D	Filarioidea	*Brugia malayi*
GCA_902646485	D	Filarioidea	*Brugia malayi*
GCA_902646495	D	Filarioidea	*Brugia malayi*
GCA_902646505	D	Filarioidea	*Brugia malayi*
GCA_902646515	D	Filarioidea	*Brugia malayi*
GCA_902646525	D	Filarioidea	*Brugia malayi*
GCA_902646565	D	Filarioidea	*Brugia malayi*
GCA_902646595	D	Filarioidea	*Brugia malayi*
GCA_902646615	D	Filarioidea	*Brugia malayi*
GCA_902646635	D	Filarioidea	*Brugia malayi*
GCA_902646655	D	Filarioidea	*Brugia malayi*
GCA_902646665	D	Filarioidea	*Brugia malayi*
GCA_902646675	D	Filarioidea	*Brugia malayi*
GCA_902646685	D	Filarioidea	*Brugia malayi*
GCA_902646695	D	Filarioidea	*Brugia malayi*
GCA_902646705	D	Filarioidea	*Brugia malayi*
GCA_902646715	D	Filarioidea	*Brugia malayi*
GCA_012030695	D	Filarioidea	*Brugia pahangi*
GCA_902646535	D	Filarioidea	*Brugia pahangi*
GCA_902648115	D	Filarioidea	*Brugia pahangi*
GCA_013366805	D	Filarioidea	*Litomosoides brasiliensis*
GCA_013365435	D	Filarioidea	*Litomosoides sigmodontis*
GCA_959347385	D	Filarioidea	*Litomosoides sigmodontis*
GCA_002204235	D	Filarioidea	*Wuchereria bancrofti*
wAmi	E	Arthropod	*Acerentomon microrhinus*
GCA_001931755	E	Arthropod	*Folsomia candida*
wNcom	E	Arthropod	*Neria commutata*
GCA_000829315	F	Arthropod	*Cimex lectularius*
GCA_902636505	F	Arthropod	*Cimex lectularius*
GCA_902636555	F	Arthropod	*Cimex lectularius*
GCA_902636575	F	Arthropod	*Cimex lectularius*
GCA_902636625	F	Arthropod	*Cimex lectularius*
GCA_902636695	F	Arthropod	*Cimex lectularius*
GCA_902636715	F	Arthropod	*Cimex lectularius*
GCA_902636785	F	Arthropod	*Cimex lectularius*
GCA_902636815	F	Arthropod	*Cimex lectularius*
GCA_902636845	F	Arthropod	*Cimex lectularius*
GCA_028571325	F	Arthropod	*Ctenocephalides canis*
wDrepF	F	Filarioidea	*Dirofilaria repens*
GCA_013366855	F	Filarioidea	*Madathamugadia hiepei*
GCA_020278625	F	Filarioidea	*Mansonella ozzardi*

GCA_020278605	F	Filarioidea	*Mansonella perstans*
GCA_023661065	F	Arthropod	*Melophagus ovinus*
GCA_029784615	F	Arthropod	*Menacanthus eurysternus*
wOc	F	Arthropod	*Osmia caerulescens*
GCA_902636395	F	Arthropod	*Osmia caerulescens*
GCA_029784575	F	Arthropod	*Penenirmus auritus*
GCA_012277295	I	Arthropod	*Ctenocephalides felis*
GCA_028571345	I	Arthropod	*Ctenocephalides orientis*
GCA_013365475	J	Arthropod	*Cruorifilaria tuberocauda*
GCA_013365495	J	Filarioidea	*Dipetalonema caudispina*
GCA_025781775	J	Filarioidea	*Dipetalonema gracile*
wBgigDune	K	Arthropod	*Bryobia gigas*
wBgigC3	K	Arthropod	*Bryobia gigas*
GCA_022836975	L	Tylenchoidea	*pool of soil nematodes*
GCA_001752665	L	Tylenchoidea	*Pratylenchus penetrans*
GCA_014534705	M	Arthropod	*Pentalonia nigronervosa*
GCA_937896465	M	Arthropod	*Pentalonia nigronervosa*
GCA_030373985	P	Arthropod	*Syringophilopsis turdi*
GCA_013317055	S	Arthropod	*Atemnus politus*
GCA_014771645	T	Arthropod	*Cimex hemipterus*
GCA_028571365	V	Arthropod	*Ctenocephalides felis*
GCA_012277315	V	Arthropod	*Ctenocephalides felis*
GCA_025021925	W	Sphaerularioidea	*Howardula sp.*
wCcry	?	Arthropod	*Cantharis cryptica*
GCA_028803215	?	Arthropod	*Flea on Himalayan marmot*
GCA_019061405	?	Arthropod	*Fragariocoptes setiger*
wMinc	?	Arthropod	*Megalothorax incertus*
GCA_029715105	?	Arthropod	*Menacanthus eurysternus*
GCA_029784595	?	Arthropod	*Meromenopon meropis*
wNca	?	Arthropod	*Neobisium carcinoides*
GCA_028803175	?	Arthropod	*Flea on Himalayan marmot*
GCA_030441635	?	Arthropod	*Tyrophagus putrescentiae*
mAli	OG	Arthropod	*Agriotes lineatus*
mBlo	OG	Arthropod	*Brontispa longissima*
mPat	OG	Arthropod	*Phosphuga atrata*
Ace	OG	Arthropod	*Ticks*
Ama	OG	Arthropod	*Ticks*
Ech	OG	Arthropod	*Ticks*
Eru	OG	Arthropod	*Ticks*

Table S3: Dereplicated *Wolbachia* genomes

Assembly name (INSDC GCA accession or given name)	Score	Primary Cluster	Secondary cluster	Supergroup	Host species
GCA_001752665	92.71	1	0	L	*Pratylenchus penetrans*
GCA_022836975	78.87	2	0	L	pool of soil nematodes
GCA_030441635	94.31	3	0	?	*Tyrophagus putrescentiae*
GCA_019061405	97.60	4	1	?	*Fragariocoptes setiger*
GCA_013365495	97.06	5	1	J	*Dipetalonema caudispina*
GCA_025781775	70.19	5	2	J	*Dipetalonema gracile*
GCA_013365475	98.49	6	0	J	*Cruorifilaria tuberocauda*
GCA_014534705	102.09	7	1	M	*Pentalonia nigronervosa*
wAmi	99.00	8	0	E	*Acerentomon microrhinus*
wNcom	101.13	9	0	E	*Neria commutata*
GCA_001931755	91.30	10	0	E	*Folsomia candida*
GCA_028803175	95.98	11	1	?	pool of marmot fleas
wBgigDune	99.74	12	1	K	*Bryobia gigas*
wMinc	99.59	13	0	?	*Megalothorax incertus*
GCA_902648165	83.59	14	1	C	*Onchocerca volvulus*
GCA_902646785	82.78	14	2	C	*Onchocerca volvulus*
GCA_000306885	98.94	14	3	C	*Onchocerca ochengi*
GCA_902646815	68.20	14	4	C	*Onchocerca volvulus*
GCA_902646835	72.24	14	5	C	*Onchocerca volvulus*
GCA_029169405	92.48	14	6	C	*Onchocerca gibsoni*
GCA_902646575	91.91	14	7	C	*Onchocerca gutturosa*
GCA_013365455	98.66	15	0	C	*Dirofilaria immitis*
wDrepC	99.11	16	0	C	*Dirofilaria repens*
GCA_004795935	101.59	17	1	D	*Brugia malayi*
GCA_012030695	101.59	17	2	D	*Brugia pahangi*
GCA_002204235	99.97	17	3	D	*Wuchereria bancrofti*
GCA_013365435	99.21	18	1	D	*Litomosoides sigmodontis*
GCA_013366805	94.21	19	0	D	*Litomosoides brasiliensis*
wCcry	98.73	20	1	?	*Cantharis cryptica*
wNca	97.43	20	2	?	*Neobisium carcinoides*
GCA_947251725	98.48	21	1	A	*Tiphia femorata*
GCA_947251795	98.03	21	2	A	*Anoplius nigerrimus*
GCA_918342435	94.97	21	3	A	*Psylliodes chrysocephala*
GCA_026768255	93.93	21	4	A	*Acromyrmex echinatior*
GCA_902646905	60.67	21	5	A	*Camponotus obliquus*
GCA_023052945	98.48	21	6	A	*Camponotus pennsylvanicus*
GCA_001648025	96.30	21	7	A	*Dactylopius coccus*
GCA_947250715	98.43	21	8	A	*Sympetrum striolatum*
GCA_019097885	89.77	21	9	A	*Pissodes strobi*
GCA_947250735	100.57	21	10	A	*Sphaerophoria taeniata*
GCA_947250775	98.27	21	11	A	*Rhinocyllus conicus*
GCA_947179565	98.49	21	12	A	*Andrena hattorfiana*
GCA_947251515	101.35	21	13	A	*Sicus ferrugineus*
GCA_010820705	81.22	21	14	A	*Apterostigma dentigerum*
GCA_014107475	102.05	21	15	A	*Drosophila sturtevanti*
GCA_014107455	103.03	21	16	A	*Drosophila nikananu*
GCA_024804185	102.24	21	17	A	*Aedes albopictus*
GCA_002379175	65.14	21	18	A	*Aedes albopictus*
GCA_014333535	101.15	21	19	A	*Anoplolepis gracilipes*
GCA_947251615	98.99	21	20	A	*Apoderus coryli*
GCA_947251645	98.97	21	21	A	*Bibio marci*
GCA_902636385	100.53	21	22	A	*Diabrotica virgifera*
GCA_014129535	99.16	21	23	A	*Drosophila neotestacea*
GCA_947179435	100.53	21	24	A	*Cheilosia soror*
GCA_947251895	98.07	21	25	A	*Bombylius major*
GCA_001675695	98.99	21	26	A	*Nomada flava*
GCA_947251665	100.16	21	27	A	*Trypoxylon clavicerum*
GCA_028982185	100.48	21	28	A	*Drosophila baimaii*
GCA_947251695	99.95	21	29	A	*Ectemnius continuus*
GCA_001983615	97.80	21	30	A	*Nasonia vitripennis*
GCA_947251755	99.51	21	31	A	*Philonthus cognatus*
GCA_028981785	100.48	21	32	A	*Drosophila leontia*

GCA_014129685	98.31	21	33	A	*Drosophila bifasciata*
GCA_000689175	81.79	21	34	A	*Glossina morsitans*
GCA_026015925	99.95	21	35	A	*Drosophila pseudotakahashii*
GCA_947250015	98.25	21	36	A	*Acrocera orbiculus*
GCA_934668665	93.67	21	37	A	unclear host - human gut microbiome
GCA_947250765	98.98	21	38	A	*Gymnosoma rotundatum*
GCA_918697765	95.71	21	39	A	*Phyllotreta cruciferae*
GCA_947250685	98.97	21	40	A	*Sphecodes monilicornis*
GCA_947251915	96.64	21	41	A	*Hylaeus communis*
GCA_008033215	102.37	21	42	A	*Drosophila ananassae*
GCA_000167475	5.87	21	43	A	*Drosophila ananassae*
GCA_017604245	102.38	21	44	A	*Rhagoletis cingulata*
GCA_902646925	75.95	21	45	A	*Diachasma alloeum*
GCA_902643455	65.39	21	46	A	*Drosophila melanogaster*
GCA_902643495	60.86	21	47	A	*Drosophila melanogaster*
GCA_947250785	102.34	21	48	A	*Ancistrocerus nigricornis*
GCA_947179405	102.36	21	49	A	*Lasioglossum malachurum*
GCA_947251965	102.32	21	50	A	*Sphecodes monilicornis*
GCA_902646345	54.15	21	51	A	*Drosophila simulans*
GCA_028982105	101.40	21	52	A	*Drosophila bicornuta*
GCA_947251775	102.42	21	53	A	*Epagoge grotiana*
GCA_947251595	102.38	21	54	A	*Eupithecia tripunctaria*
GCA_009732755	102.36	21	55	A	*Haematobia irritans*
GCA_902713635	95.63	21	56	A	*Cardiocondyla obscurior*
GCA_947251485	100.85	21	57	A	*Scambus nigricans*
GCA_947251435	98.97	21	58	A	*Epistrophe grossularia*
GCA_018454475	98.46	21	59	A	*Rhagoletis cerasi*
GCA_902636435	66.61	21	60	A	*Lasioglossum albipes*
GCA_947250585	96.23	21	61	A	*Lasioglossum morio*
GCA_947250805	101.27	21	62	A	*Endotricha flammealis*
GCA_006542295	102.84	21	63	A	*Carposina sasakii*
GCA_029238795	73.33	21	64	A	*Eurosta solidaginis*
GCA_009012935	97.76	21	65	A	*Nasonia oneida*
GCA_902648465	58.70	21	66	A	Apoidea sp.
GCA_947251635	99.68	21	67	A	*Icerya purchasi*
GCA_947250665	95.67	21	68	A	*Volucella inflata*
GCA_947251545	98.99	21	69	A	*Anomoia purmunda*
GCA_936270435	92.99	22	0	?	*Oedothorax gibbosus*
GCA_936270145	101.40	23	0	?	*Oedothorax gibbosus*
GCA_001027565	101.38	24	1	B	*Armadillidium vulgare*
GCA_003344345	67.38	24	2	B	*Cylisticus convexus*
GCA_947179555	100.38	25	1	B	*Chorthippus brunneus*
GCA_947250645	93.59	25	2	B	*Eucosma cana*
GCA_007115045	94.10	25	3	B	*Nilaparvata lugens*
GCA_018224395	95.83	25	4	B	*Homalodisca vitripennis*
GCA_003999585	85.30	25	5	B	*Bemisia tabaci*
GCA_900097055	77.41	25	6	B	*Bemisia tabaci*
GCA_007115015	100.71	25	7	B	*Laodelphax striatellus*
GCA_013458815	100.72	25	8	B	*Diaphorina citri*
GCA_000376585	100.66	25	9	B	*Drosophila simulans*
GCA_018491625	102.03	25	10	B	*Anopheles moucheti*
GCA_019665805	101.90	25	11	B	*Aedes aegypti*
GCA_947179575	99.58	25	12	B	*Erebia ligea*
GCA_018141665	103.07	25	13	B	*Spodoptera picta*
GCA_018491735	101.58	25	14	B	*Anopheles demeilloni*
GCA_902646255	99.11	25	15	B	*Tetranychus urticae*
GCA_947251495	102.06	25	16	B	*Pammene fasciana*
GCA_000723225	102.87	25	17	B	*Culex molestus*
GCA_008245065	97.09	25	18	B	*Chrysomya megacephala*
GCA_947250555	98.66	25	19	B	*Pandemis corylana*
GCA_025617515	97.38	25	20	B	*Oryzaephilus surinamensis*
GCA_947250515	100.23	25	21	B	*Catoptria pinella*
GCA_947250595	103.10	25	22	B	*Campaea margaritaria*
GCA_947250655	103.12	25	23	B	*Watsonalla binaria*
GCA_947251875	103.07	25	24	B	*Lycaena phlaeas*
GCA_018454445	98.90	25	25	B	*Rhagoletis cerasi*
GCA_000333775	101.07	25	26	B	*Hypolimnas bolina*
GCA_947251865	102.11	25	27	B	*Protocalliphora azurea*

GCA_024205405	100.18	25	28	B	*Tetranychus truncatus*
GCA_006334525	97.41	25	29	B	*Leptopilina clavipes*
GCA_947250615	98.21	25	30	B	*Rhopobota naevana*
GCA_947250575	97.65	25	31	B	*Episyrphus balteatus*
GCA_947179355	97.47	25	32	B	*Sphaerophoria taeniata*
GCA_947251465	94.87	25	33	B	*Melanostoma mellinum*
GCA_001648015	39.93	25	34	B	*Dactylopius coccus*
GCA_947251585	100.26	25	35	B	*Ischnura elegans*
GCA_947251735	100.67	25	36	B	*Endotricha flammealis*
GCA_947250635	102.45	25	37	B	*Parapoynx stratiotata*
GCA_947179345	97.46	25	38	B	*Philonthus cognatus*
GCA_947251825	94.32	25	39	B	*Camptogramma bilineatum*
GCA_947251845	97.64	25	40	B	*Athalia cordata*
GCA_947250535	97.70	25	41	B	*Euphydryas aurinia*
GCA_029856955	88.77	25	42	B	*Frankliniella intonsa*
GCA_020405475	94.42	25	43	B	*Tribolium confusum*
GCA_001439985	102.04	25	44	B	*Trichogramma pretiosum*
GCA_014771645	100.17	26	0	T	*Cimex hemipterus*
GCA_030373985	101.11	27	1	P	*Syringophilopsis turdi*
GCA_020278605	94.83	28	1	F	*Mansonella perstans*
GCA_020278625	93.83	28	2	F	*Mansonella ozzardi*
GCA_023661085	95.39	29	1	F	*Osmia caerulescens*
GCA_023661065	96.89	29	2	F	*Melophagus ovinus*
GCA_029784575	90.48	29	3	F	*Penenirmus auritus*
GCA_029784615	100.00	29	4	F	*Menacanthus eurysternus*
GCA_000829315	100.65	30	1	F	*Cimex lectularius*
GCA_013366855	80.54	30	2	F	*Madathamugadia hiepei*
GCA_028571325	100.66	30	3	F	*Ctenocephalides canis*
GCA_028571365	99.42	31	1	V	*Ctenocephalides felis*
GCA_013317055	17.73	32	0	S	*Atemnus politus*
GCA_028571345	96.85	33	0	I	*Ctenocephalides orientis*
GCA_012277295	94.97	34	0	I	*Ctenocephalides felis*
GCA_029715105	88.89	35	0	?	*Menacanthus eurysternus*
GCA_029784595	96.87	36	0	?	*Meromenopon meropis*
GCA_025021925	92.36	37	0	W	*Howardula sp.*

Table S4: Contigs removed from filarial nematode genomes as likely contaminants.

**Species**	**Removed contigs/scaffolds**
Brugia timori	UZAG01000844.1, UZAG01001276.1, UZAG01002041.1, UZAG01004090.1, UZAG01008060.1, UZAG01009429.1, UZAG01009651.1, UZAG01014557.1, UZAG01016088.1, UZAG01017795.1, UZAG01021318.1, UZAG01022991.1
Cruorifilaria (Cr.) tuberocauda	JABVXU010000032.1 ,JABVXU010000040.1, JABVXU010000130.1, JABVXU010000808.1
Dipetalonema (Dp.) caudispina	JABVXV010000687.1
Dirofilaria (Dr.) repens	SNRZ01000060.1, SNRZ01000108.1, SNRZ01000191.1, SNRZ01000310.1, SNRZ01000353.1
Litomosoides (Li.) brasiliensis	JABVXW010000830.1, JABVXW010000864.1, JABVXW010000878.1, JABVXW010000892.1, JABVXW010000895.1, JABVXW010000908.1, JABVXW010000910.1, JABVXW010000912.1
Madathamugadia (Md.) hiepei	JABVXS010003625.1, JABVXS010006396.1, JABVXS010011293.1, JABVXS010012003.1
Mansonella (Ma.) ozzardi	JARVGP010003176.1
Mansonella (Ma.) perstans	CANNZR030003124.1, CANNZR030003977.1, CANNZR030004819.1
Onchocerca lupi	JAOVVO010000003.1, JAOVVO010000020.1, JAOVVO010000458.1, JAOVVO010000729.1, JAOVVO010000989.1
Onchocerca ochengi	FJNM01008945.1, FJNM01012418.1, FJNM01013073.1 ,FJNM01013466.1, FJNM01013675.1, FJNM01015256.1, FJNM01015404.1, FJNM01016510.1, FJNM01016599.1, FJNM01017051.1, FJNM01017295.1, FJNM01017368.1, FJNM01017659.1, FJNM01018128.1, FJNM01018685.1
Onchocerca volvulus	HG738773.1

Table S5: Numbers and classification of NUWTs detected in each filarial nematode species genome

Species	?	C	D	F	J	Grand Total
Acanthocheilonema viteae	6	29	9	8	16	68
Brugia malayi	30		692	10		732
Brugia pahangi	12		782	9		803
Brugia timori	9		179	1		189
Cercopithifilaria (Ce.) johnstoni	8	14	6	2	11	41
Cruorifilaria (Cr.) tuberocauda	8	20	8	7	245	288
Dipetalonema (Dp.) caudispina	5	16	7	8	60	96
Dirofilaria (Dr.) immitis	10	507	3	70	8	598
Dirofilaria (Dr.) repens	25	551	5	193	8	782
Elaeophora elaphii	11	56	8	1	92	168
Litomosoides (Li.) brasiliensis	9	41	113	2	4	169
Litomosoides (Li.) sigmodontis	4	17	8	15		44
Loa (Lo.) loa	4	4	29			37
Madathamugadia (Md.) hiepei	21	242	61	74	54	452
Mansonella (Ma.) ozzardi	14	6	8	189		217
Mansonella (Ma.) perstans	13	5	7	124	1	150
Onchocerca flexuosa	7	536	1	13	9	566
Onchocerca lupi	10	713	4	11	12	750
Onchocerca ochengi	10	711	2	10	8	741
Onchocerca volvulus	8	787	5	7	11	818
Wuchereria bancrofti	10		153	21	1	185
**Grand Total**	**234**	**4255**	**2090**	**775**	**540**	**7894**

Each cell reports the number of NUWTs (for each *Wolbachia* supergroup and overall).

Table S6: Span of NUWTs detected in each filarial nematode species' genome.

Species	?	C	D	F	J	Grand Total
Acanthocheilonema viteae	1543	10897	2412	4941	5486	25279
Brugia malayi	6523		306793	4059		317375
Brugia pahangi	3176		336865	3326		343367
Brugia timori	2200		61788	494		64482
Cercopithifilaria (Ce.) johnstoni	1882	5249	1338	512	5519	14500
Cruorifilaria (Cr.) tuberocauda	1984	5728	1763	7379	150607	167461
Dipetalonema (Dp.) caudispina	1137	4475	1744	10757	16017	34130
Dirofilaria (Dr.) immitis	2651	153678	584	20868	2439	180220
Dirofilaria (Dr.) repens	7346	186141	1437	67006	1752	263682
Elaeophora elaphii	2979	18545	2312	345	32611	56792
Litomosoides (Li.) brasiliensis	2129	30154	44650	415	1212	78560
Litomosoides (Li.) sigmodontis	849	7260	2124	7738		17971
Loa (Lo.) loa	1002	905	10068			11975
Madathamugadia (Md.) hiepei	5564	163207	37438	22479	31461	260149
Mansonella (Ma.) ozzardi	4123	1843	2094	58728		66788
Mansonella (Ma.) perstans	4039	1745	1836	41242	230	49092
Onchocerca flexuosa	2081	154720	252	6469	2311	165833
Onchocerca lupi	2797	205963	1592	9406	3913	223671
Onchocerca ochengi	3162	203950	990	5603	2055	215760
Onchocerca volvulus	2685	245938	1719	3094	2931	256367
Wuchereria bancrofti	2280		68883	18508	362	90033
**Grand Total**	62132	1400398	888682	293369	258906	2903487

Each cell reports the span in bases of sequence attributed to NUWTs (for each *Wolbachia* supergroup and overall).

Table S7: Average length of NUWTs

Species	?	C	D	F	J	Grand Total
Acanthocheilonema viteae	257.2	375.8	268.0	617.6	342.9	371.8
Brugia malayi	217.4		443.3	405.9		433.6
Brugia pahangi	264.7		430.8	369.6		427.6
Brugia timori	244.4		345.2	494.0		341.2
Cercopithifilaria (Ce.) johnstoni	235.3	374.9	223.0	256.0	501.7	353.7
Cruorifilaria (Cr.) tuberocauda	248.0	286.4	220.4	1054.1	614.7	581.5
Dipetalonema (Dp.) caudispina	227.4	279.7	249.1	1344.6	267.0	355.5
Dirofilaria (Dr.) immitis	265.1	303.1	194.7	298.1	304.9	301.4
Dirofilaria (Dr.) repens	293.8	337.8	287.4	347.2	219.0	337.2
Elaeophora elaphii	270.8	331.2	289.0	345.0	354.5	338.0
Litomosoides (Li.) brasiliensis	236.6	735.5	395.1	207.5	303.0	464.9
Litomosoides (Li.) sigmodontis	212.3	427.1	265.5	515.9		408.4
Loa (Lo.) loa	250.5	226.3	347.2			323.6
Madathamugadia (Md.) hiepei	265.0	674.4	613.7	303.8	582.6	575.6
Mansonella (Ma.) ozzardi	294.5	307.2	261.8	310.7		307.8
Mansonella (Ma.) perstans	310.7	349.0	262.3	332.6	230.0	327.3
Onchocerca flexuosa	297.3	288.7	252.0	497.6	256.8	293.0
Onchocerca lupi	279.7	288.9	398.0	855.1	326.1	298.2
Onchocerca ochengi	316.2	286.8	495.0	560.3	256.9	291.2
Onchocerca volvulus	335.6	312.5	343.8	442.0	266.5	313.4
Wuchereria bancrofti	228.0		450.2	881.3	362.0	486.7
**Grand Total**	**265.5**	**329.1**	**425.2**	**378.5**	**479.5**	**367.8**

Each cell reports the length in bases of NUWTs (for each *Wolbachia* supergroup and overall).

Table S8: NUWTs from different *Wolbachia* supergroups

Originating *Wolbachia* supergroup	Number of NUWTs	Average length of NUWTs (bp)
**?**	234	265.5
**C**	4255	329.1
**D**	2090	425.2
**F**	775	378.5
**J**	540	479.5
**Grand Total**	**7894**	**367.8**

Supplementary Figures

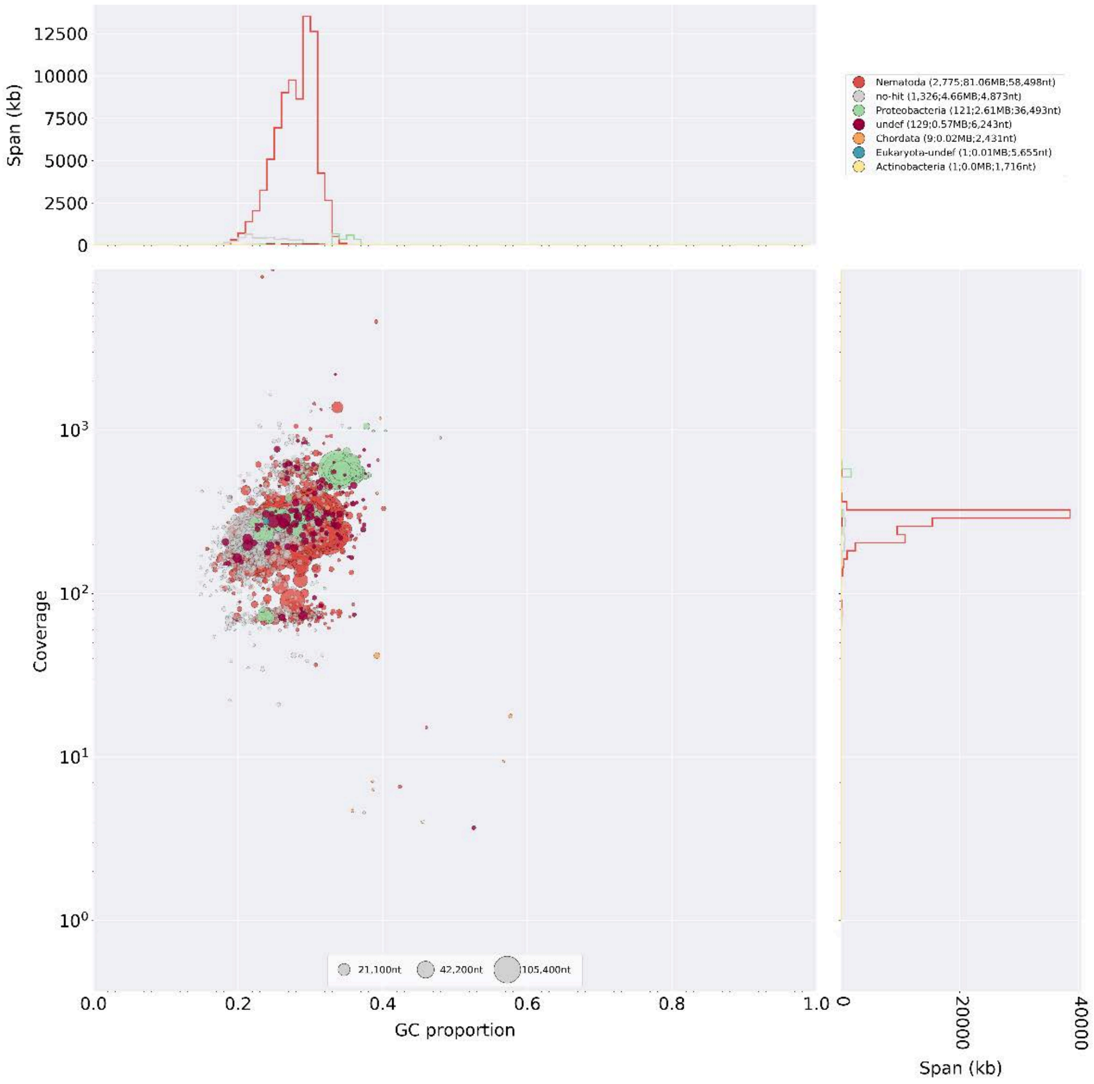

Figure S1: BlobTools plot of initial *Dirofilaria repens* assembly

A BlobTools (v1) plot of the *Dr. repens* assembly showing (red) contigs assigned to Nematoda (i.e. the nematode host) and (green) contigs assigned to Proteobacteria (i.e. *Wolbachia*). The Protobacteria contigs (coloured green) form two clusters, one at ~300 fold coverage and 35% GC, and one at ~200 fold coverage and 28% GC. These correspond to the C supergroup (high coverage) and F supergroup (low coverage) *Wolbachia* coinfecting this sample.

A

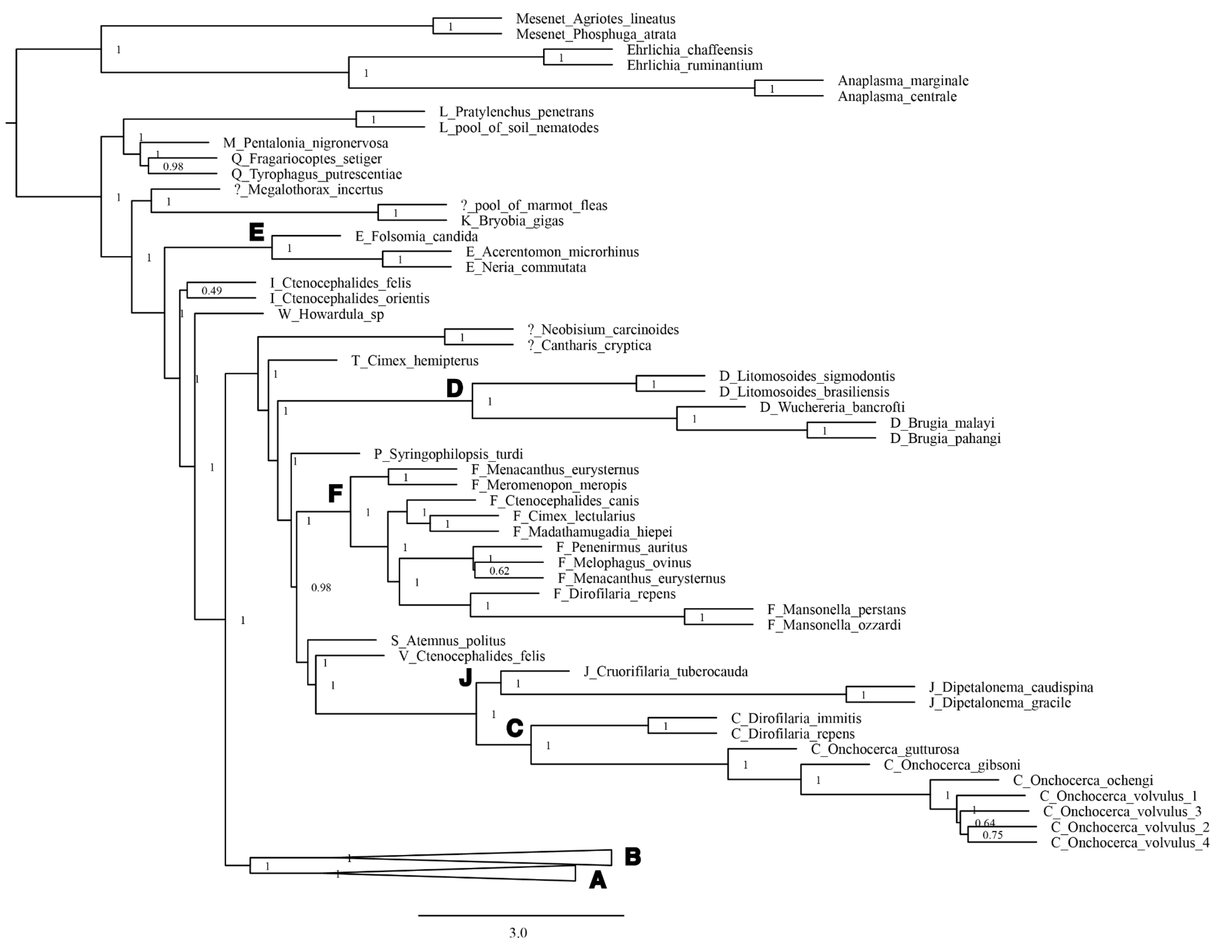
Mesenet_Agriotes_lineatus
Mesenet_Phosphuga_atrata
Ehrlichia_chaffeensis
Ehrlichia_ruminantium
Anaplasma_marginale
Anaplasma_centrale
L_Pratylenchus_penetrans
L_pool_of_soil_nematodes
M_Pentalonia_nigronervosa
Q_Fragariocoptes_setiger
Q_Tyrophagus_putrescentiae
?_Megalothorax_incertus
?_pool_of_marmot_fleas
K_Bryobia_gigas
E
E_Folsomia_candida
E_Acerentomon_microrhinus
E_Neria_commutata
I_Ctenocephalides_felis
I_Ctenocephalides_orientis
W_Howardula_sp
?_Neobisium_carcinoides
?_Cantharis_cryptica
T_Cimex_hemipterus
D
D_Litomosoides_sigmodontis
D_Litomosoides_brasiliensis
D_Wuchereria_bancrofti
D_Brugia_malayi
D_Brugia_pahangi
P_Syringophilopsis_turdi
F
F_Menacanthus_eurysternus
F_Meromenopon_meropis
F_Ctenocephalides_canis
F_Cimex_lectularius
F_Madathamugadia_hiepei
F_Penenirmus_auritus
F_Melophagus_ovinus
F_Menacanthus_eurysternus
F_Dirofilaria_repens
F_Mansonella_perstans
F_Mansonella_ozzardi
S_Atemnus_politus
V_Ctenocephalides_felis
J
J_Cruorifilaria_tuberocauda
J_Dipetalonema_caudispina
J_Dipetalonema_gracile
C
C_Dirofilaria_immitis
C_Dirofilaria_repens
C_Onchocerca_gutturosa
C_Onchocerca_gibsoni
C_Onchocerca_ochengi
C_Onchocerca_volvulus_1
C_Onchocerca_volvulus_3
C_Onchocerca_volvulus_2
C_Onchocerca_volvulus_4
B
A
3.0

B

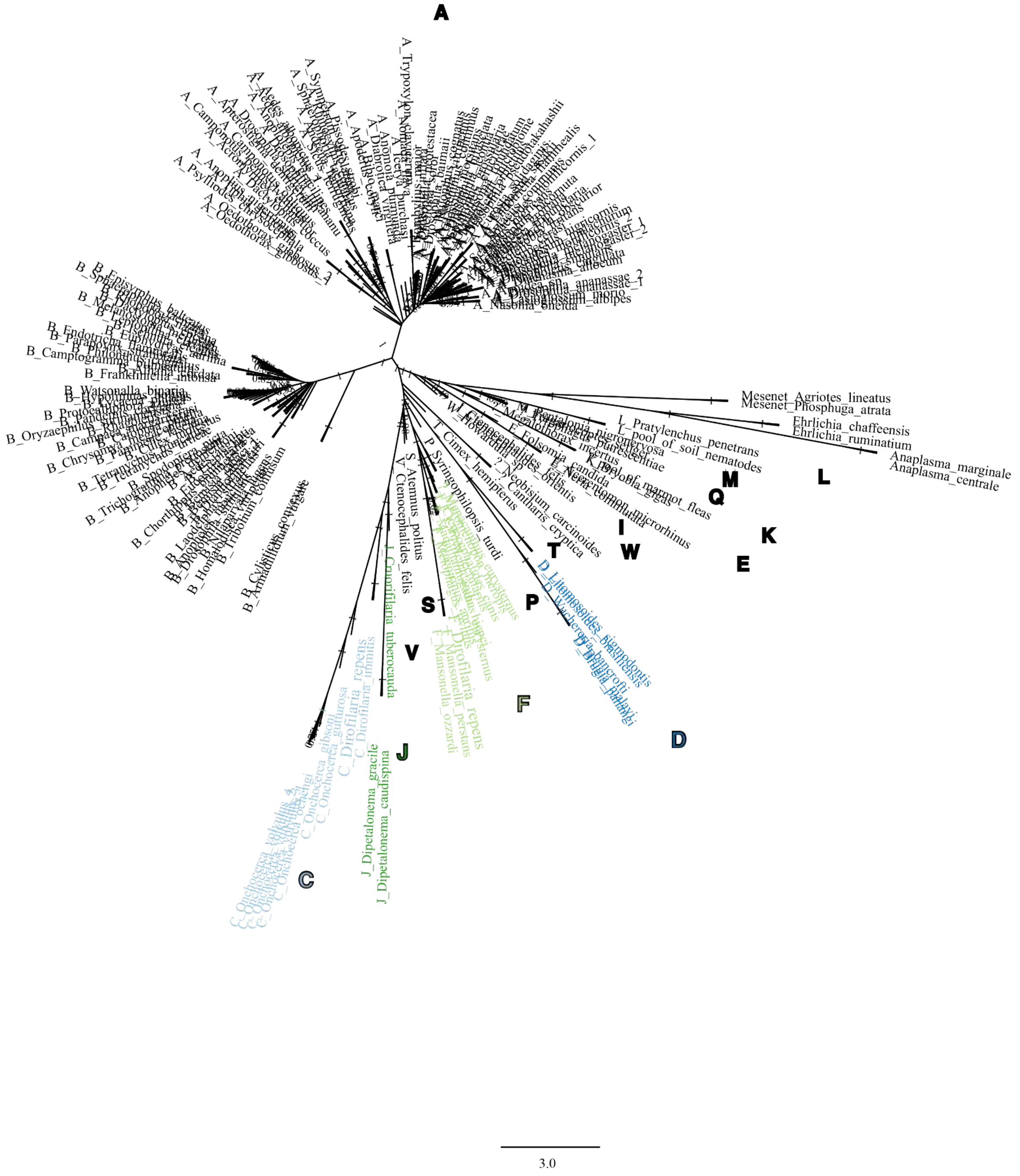
A
B
C
D
E
F
I
J
K
L
M
P
Q
S
T
V
W
Mesenet_Agriotes_lineatus
Mesenet_Phosphuga_atrata
Ehrlichia_chaffeensis
Ehrlichia_ruminatium
Anaplasma_marginale
Anaplasma_centrale
L_Pratylenchus_penetrans
L_pool_of_soil_nematodes
Q_pool_of_marmot_fleas
T_Cimex_hemipterus
P_Syringophilopsis_turdi
S_Atemnus_politus
V_Ctenocephalides_felis
J_Cruorifilaria_tuberocauda
J_Dipetalonema_gracile
J_Dipetalonema_caudispina
C_Dirofilaria_repens
C_Dirofilaria_immitis
F_Dirofilaria_repens
F_Mansonella_perstans
F_Mansonella_ozzardi
D_Litomosoides_sigmodontis
A_Nasonia_oneida
A_Oedothorax_gibbosus_1
A_Oedothorax_gibbosus_2
B_Watsonalla_binaria
B_Frankliniella_intonsa
B_Cylisticus_convexus
B_Armadillidium_vulgare
3.0

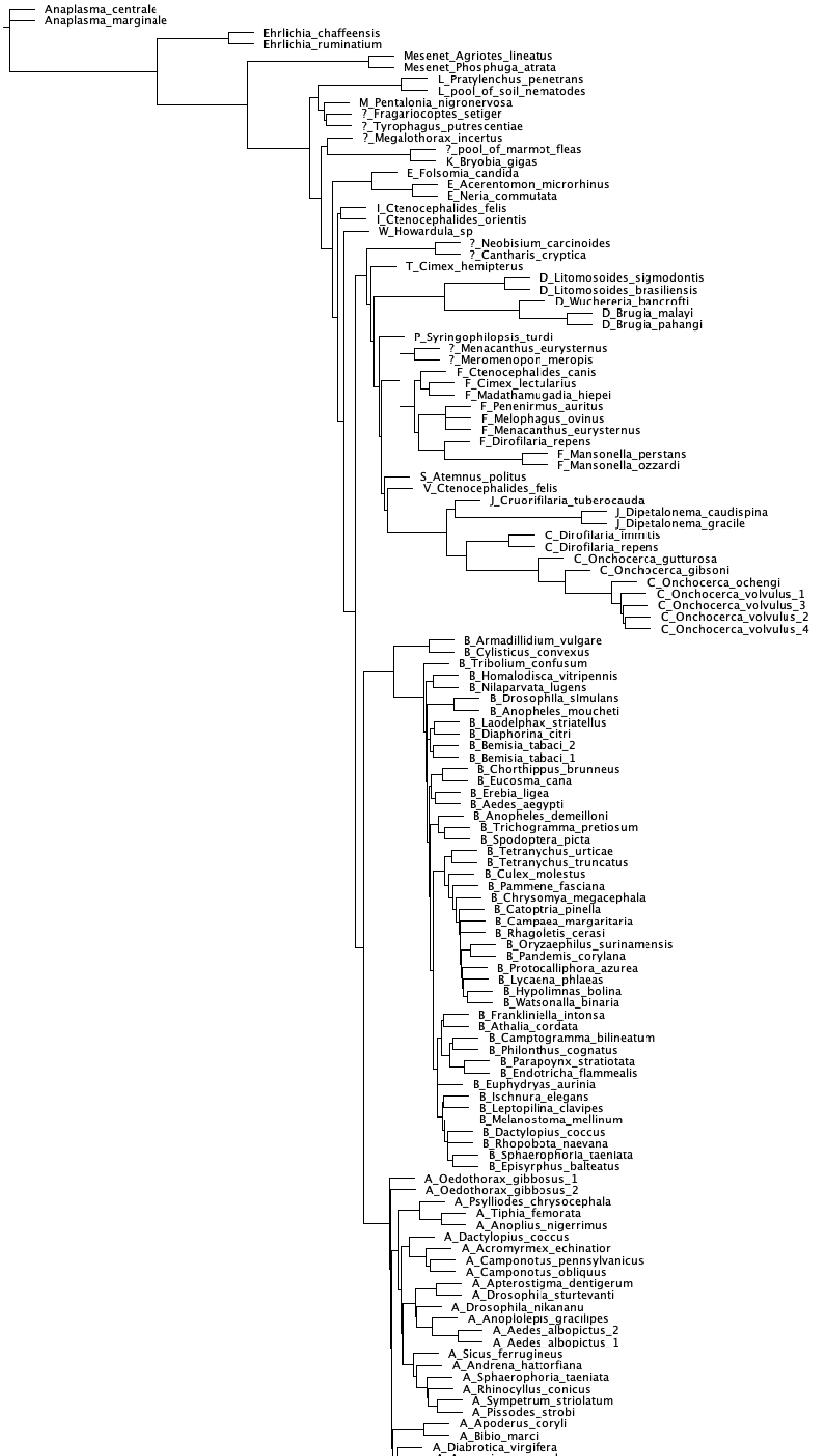
Anaplasma_centrale
Anaplasma_marginale
Ehrlichia_chaffeensis
Ehrlichia_ruminatium
Mesenet_Agriotes_lineatus
Mesenet_Phosphuga_atrata
L_Pratylenchus_penetrans
L_pool_of_soil_nematodes
M_Pentalonia_nigronervosa
?_Fragariocoptes_setiger
?_Tyrophagus_putrescentiae
?_Megalothorax_incertus
?_pool_of_marmot_fleas
K_Bryobia_gigas
E_Folsomia_candida
E_Acerentomon_microrhinus
E_Neria_commutata
I_Ctenocephalides_felis
I_Ctenocephalides_orientis
W_Howardula_sp
?_Neobisium_carcinoides
?_Cantharis_cryptica
T_Cimex_hemipterus
D_Litomosoides_sigmodontis
D_Litomosoides_brasiliensis
D_Wuchereria_bancrofti
D_Brugia_malayi
D_Brugia_pahangi
P_Syringophilopsis_turdi
?_Menacanthus_eurysternus
?_Meromenopon_meropis
F_Ctenocephalides_canis
F_Cimex_lectularius
F_Madathamugadia_hiepei
F_Penenirmus_auritus
F_Melophagus_ovinus
F_Menacanthus_eurysternus
F_Dirofilaria_repens
F_Mansonella_perstans
F_Mansonella_ozzardi
S_Atemnus_politus
V_Ctenocephalides_felis
J_Cruorifilaria_tuberocauda
J_Dipetalonema_caudispina
J_Dipetalonema_gracile
C_Dirofilaria_immitis
C_Dirofilaria_repens
C_Onchocerca_gutturosa
C_Onchocerca_gibsoni
C_Onchocerca_ochengi
C_Onchocerca_volvulus_1
C_Onchocerca_volvulus_3
C_Onchocerca_volvulus_2
C_Onchocerca_volvulus_4
B_Armadillidium_vulgare
B_Cylisticus_convexus
B_Tribolium_confusum
B_Homalodisca_vitripennis
B_Nilaparvata_lugens
B_Drosophila_simulans
B_Anopheles_moucheti
B_Laodelphax_striatellus
B_Diaphorina_citri
B_Bemisia_tabaci_2
B_Bemisia_tabaci_1
B_Chorthippus_brunneus
B_Eucosma_cana
B_Erebia_ligea
B_Aedes_aegypti
B_Anopheles_demeilloni
B_Trichogramma_pretiosum
B_Spodoptera_picta
B_Tetranychus_urticae
B_Tetranychus_truncatus
B_Culex_molestus
B_Pammene_fasciana
B_Chrysomya_megacephala
B_Catoptria_pinella
B_Campaea_margaritaria
B_Rhagoletis_cerasi
B_Oryzaephilus_surinamensis
B_Pandemis_corylana
B_Protocalliphora_azurea
B_Lycaena_phlaeas
B_Hypolimnas_bolina
B_Watsonalla_binaria
B_Frankliniella_intonsa
B_Athalia_cordata
B_Camptogramma_bilineatum
B_Philonthus_cognatus
B_Parapoynx_stratiotata
B_Endotricha_flammealis
B_Euphydryas_aurinia
B_Ischnura_elegans
B_Leptopilina_clavipes
B_Melanostoma_mellinum
B_Dactylopius_coccus
B_Rhopobota_naevana
B_Sphaerophoria_taeniata
B_Episyrphus_balteatus
A_Oedothorax_gibbosus_1
A_Oedothorax_gibbosus_2
A_Psylliodes_chrysocephala
A_Tiphia_femorata
A_Anoplius_nigerrimus
A_Dactylopius_coccus
A_Acromyrmex_echinatior
A_Camponotus_pennsylvanicus
A_Camponotus_obliquus
A_Apterostigma_dentigerum
A_Drosophila_sturtevanti
A_Drosophila_nikananu
A_Anoplolepis_gracilipes
A_Aedes_albopictus_2
A_Aedes_albopictus_1
A_Sicus_ferrugineus
A_Andrena_hattorfiana
A_Sphaerophoria_taeniata
A_Rhinocyllus_conicus
A_Sympetrum_striolatum
A_Pissodes_strobi
A_Apoderus_coryli
A_Bibio_marci
A_Diabrotica_virgifera

Figure S2: Genome phylogeny of *Wolbachia*

A. Summary phylogeny of 1,444 *Wolbachia* genomes. The phylogeny was rooted with genomes from seven *Anaplasma*, *Ehrlichia* and *Mesenet* species. Supergroups are indicated with bold letters.

B. Unrooted phylogeny of 167 selected *Wolbachia* genomes (as in Figure 1B) with sources named. Supergroups are indicated with bold letters.

C. Rooted phylogeny of 167 selected *Wolbachia* genomes (as in Figure 1B) with sources named.

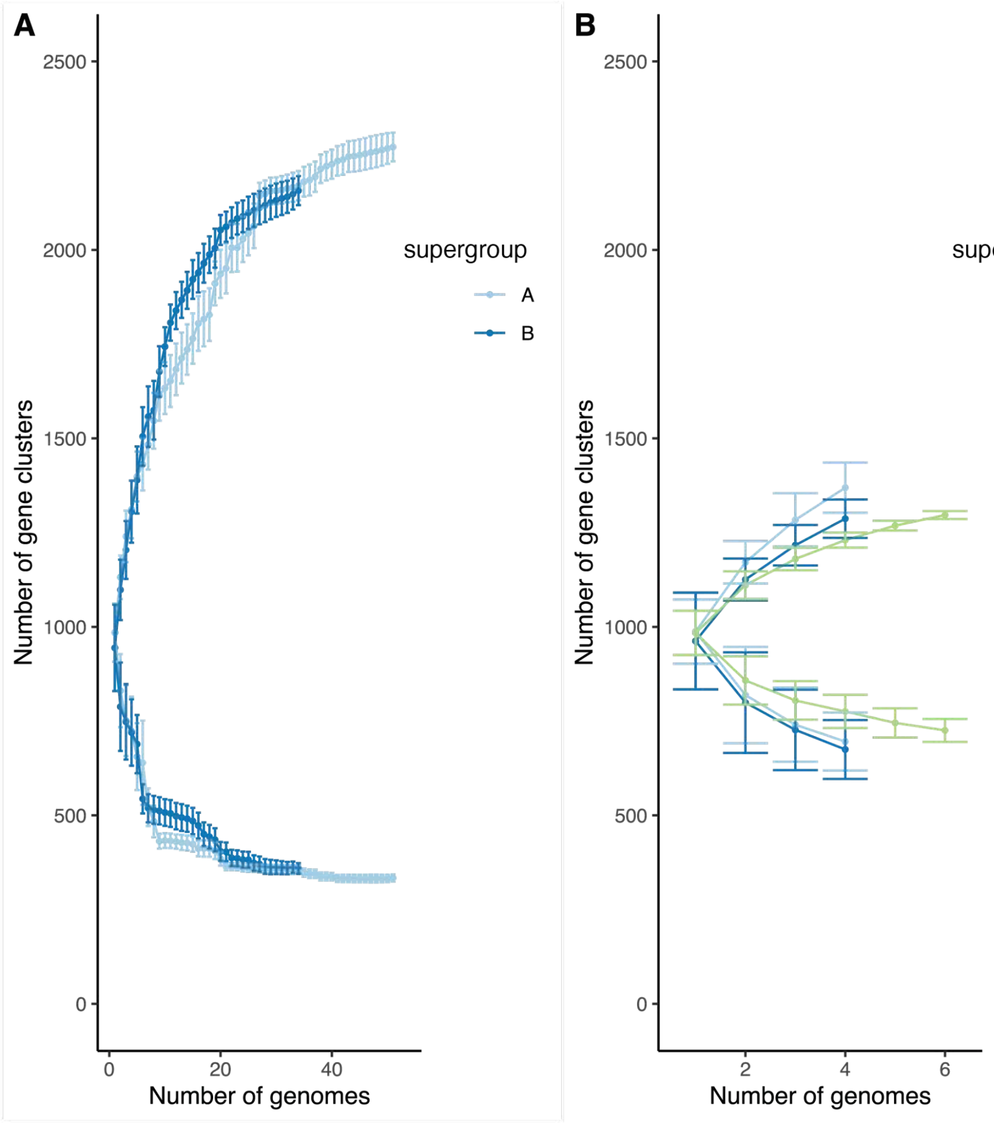

Figure S3: *Wolbachia* proteome clustering

A. Rarefaction curves describing the proteome diversity found across *Wolbachia* and within supergroup A and B. *Wolbachia* proteomes selected with dRep and deemed near-complete were added one by one and the number of clusters with >1 member tallied. The standard deviations are derived from 50,000 repetitions of the clustering with randomised addition order of genomes. The upper curves are for orthogroups with >1 member, while the lower curves indicate the number of singleton sequences.

B. Rarefaction curves describing the proteome diversity found within supergroup C, D and F. *Wolbachia* proteomes selected with dRep and deemed near-complete were added one by one and the number of clusters with >1 member tallied. The standard deviations are derived from 50,000 repetitions of the clustering with randomised addition order of genomes. The upper curves are for orthogroups with >1 member, while the lower curves indicate the number of singleton sequences.

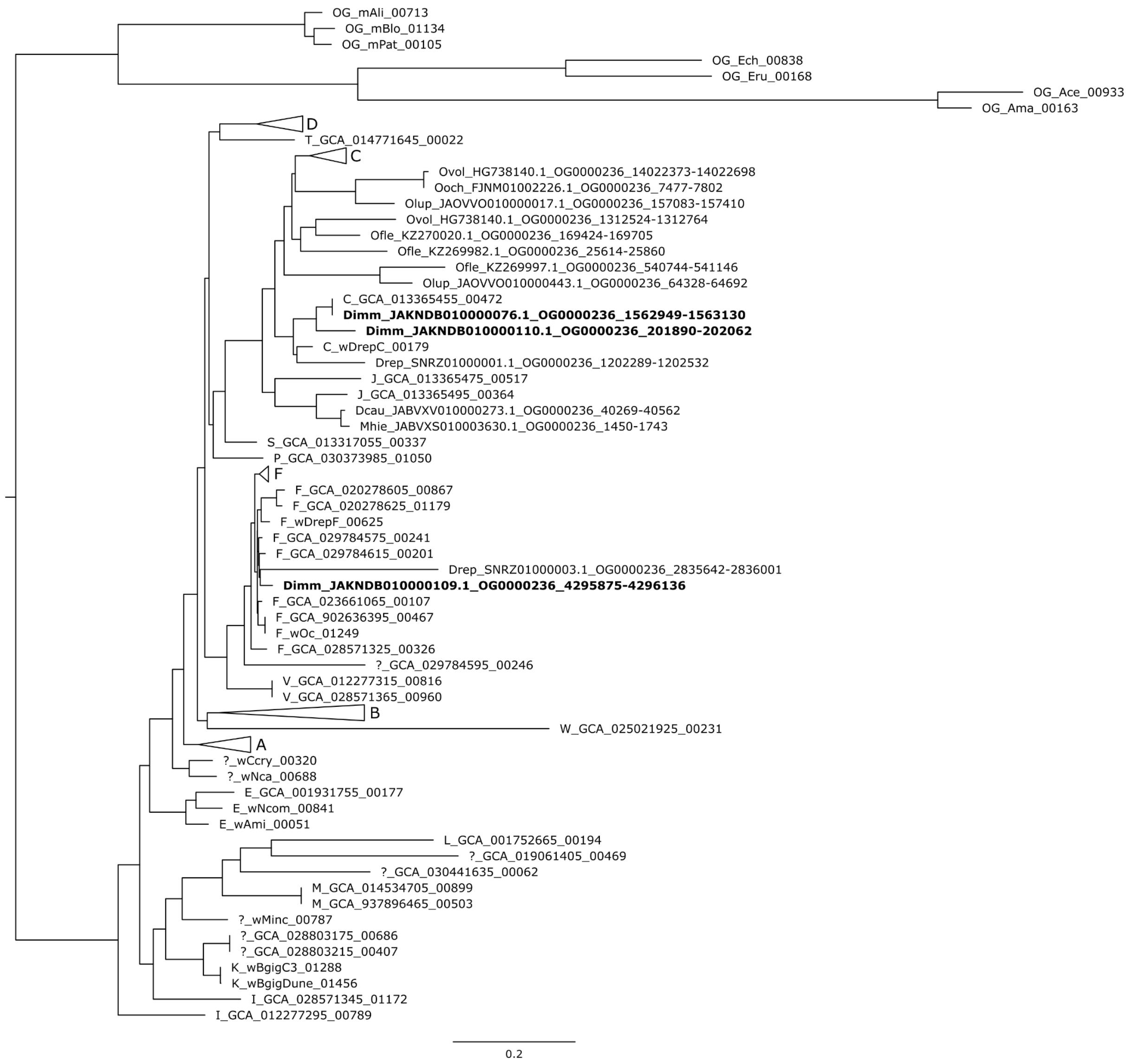

Figure S4: Phylogenetic tree of orthologous family OG0000236 and NUWTs

Phylogenetic tree of dihydrolipoyl dehydrogenase (OG0000236), illustrating the phylogenetic placement of NUWTs deriving from both C and F *Wolbachia* in *Dirofilaria immitis* (highlighted in bold).

Sequences derived from living *Wolbachia* are indicated by the supergroup and host species nomenclature followed by the locus number (e.g. “E_wNcom_00841”) or supergroup and GCA nomenclature followed by the locus number (e.g. “I_GCA012277295_00789”), while NUWTS are indicated by the nematode species abbreviation and their location (e.g. “Dimm_JAKNDB010000109.1_OG0000236_4295875-4296316” is from *D. immitis*, contig JAKNDB010000109.1; it matches OG0000236 and is from bases 4295875-4296316 in the contig).

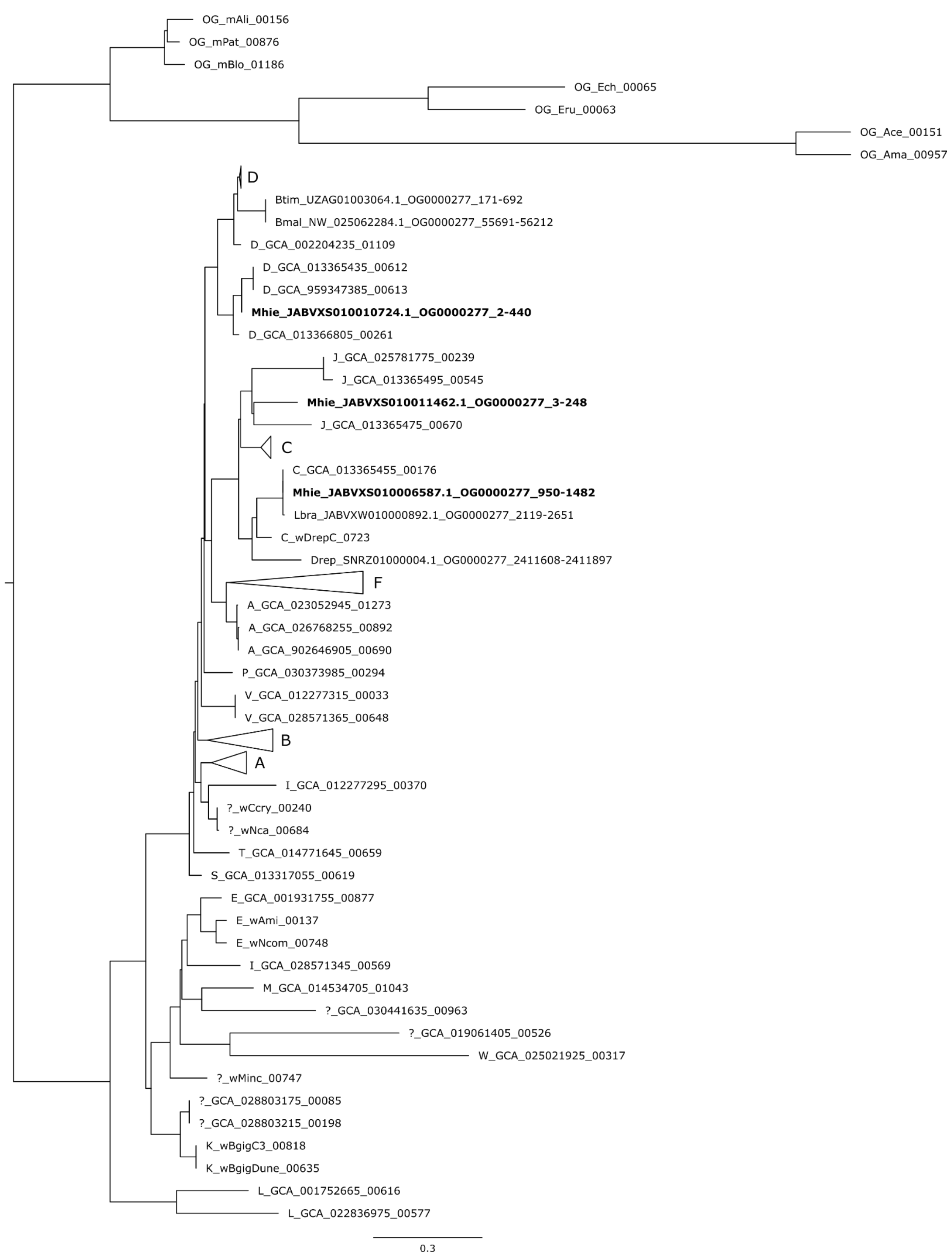

Figure S5: Phylogenetic tree of orthologous family OG0000277 and NUWTS

Phylogenetic tree of peptide deformylase (OG0000277) showing the phylogenetic placement of NUWTs from *Madathamugadia hiepeia*. The NUWTs likely derive from C, D and J *Wolbachia*. Nomenclature as in Figure S4.

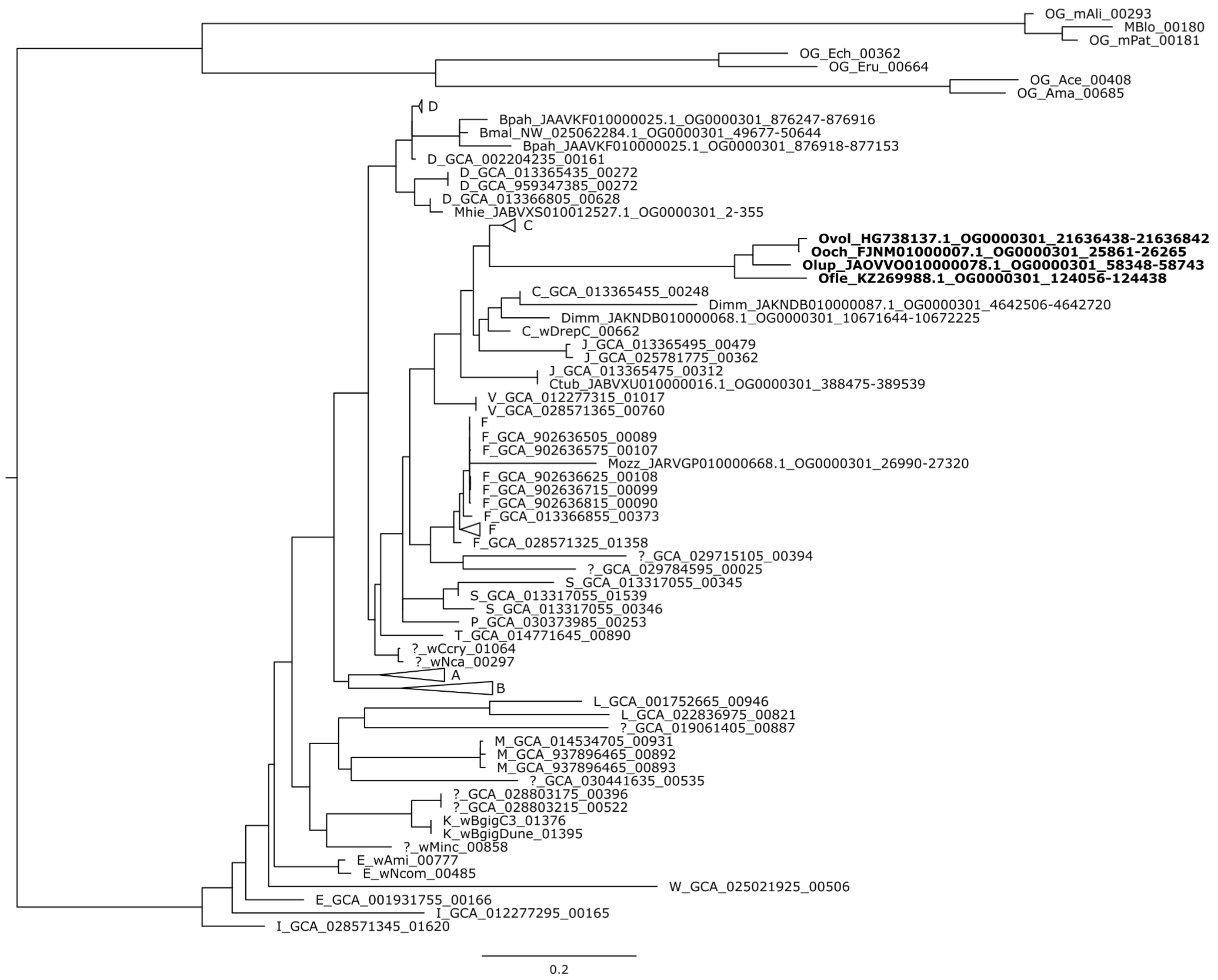

Figure S6: Phylogenetic tree of orthologous family OG0000301 and NUWTs

Phylogenetic tree of DNA-directed RNA polymerase subunit alpha (OG0000301) showing the phylogenetic placement of NUWTs from four *Onchocerca* species. Nomenclature as in Figure S4.

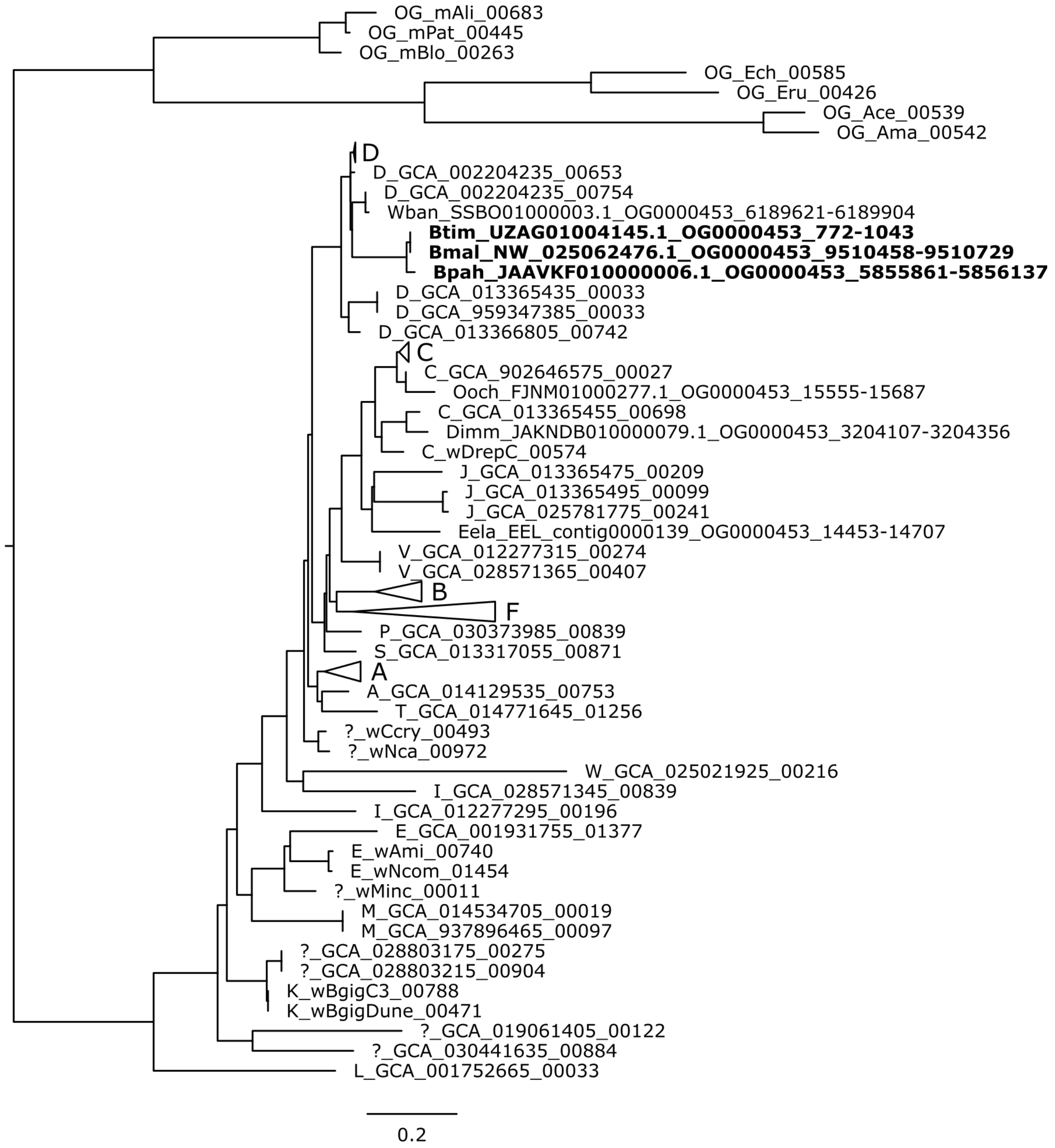

Figure S7: Phylogenetic tree of orthologous family OG0000453 and NUWTs

Phylogenetic tree of signal peptidase I (OG0000453) showing the phylogenetic placement of NUWTs from three *Brugia* species. Nomenclature as in Figure S4.

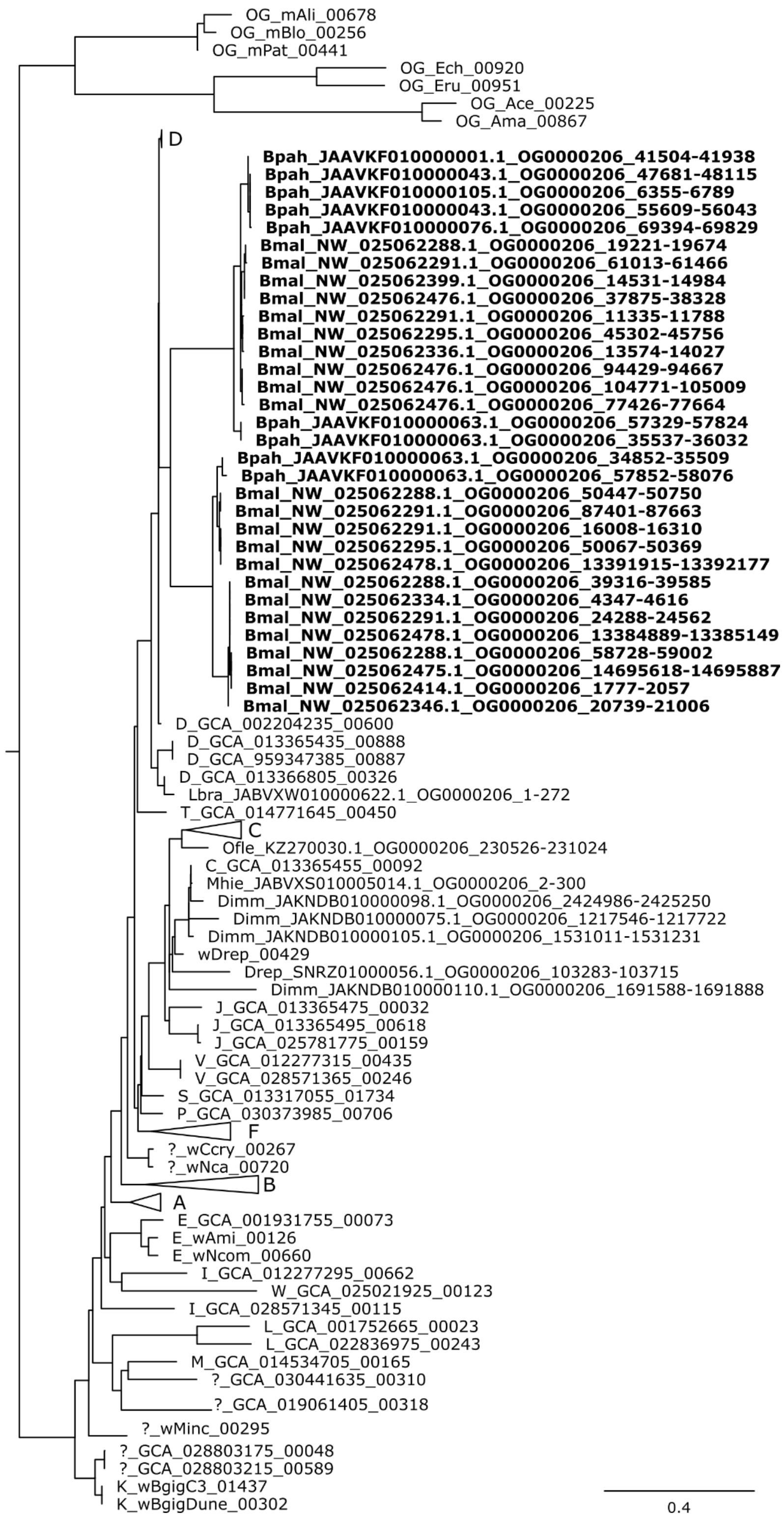

Figure S8: Phylogenetic tree of orthologous family OG0000206 and NUWTs

Phylogenetic tree of ATP-dependent zinc metalloprotease FtsH (OG0000206), showing the phylogenetic placement of multi-copy NUWTs from two *Brugia* species. Nomenclature as in Figure S4.
